# Impact of Alternative Splicing on the Human Proteome

**DOI:** 10.1016/j.celrep.2017.07.025

**Published:** 2017-08-01

**Authors:** Yansheng Liu, Mar Gonzàlez-Porta, Sergio Santos, Alvis Brazma, John C. Marioni, Ruedi Aebersold, Ashok R. Venkitaraman, Vihandha O. Wickramasinghe

**Affiliations:** 1 Department of Biology, Institute of Molecular Systems Biology, ETH Zurich, Zurich, Switzerland; 2 European Molecular Biology Laboratory-European Bioinformatics Institute (EMBL-EBI), Hinxton, UK; 3 The Medical Research Council Cancer Unit, University of Cambridge, Cambridge CB2 0XZ, UK; 4 RNA Biology and Cancer Laboratory, Peter MacCallum Cancer Centre, Melbourne, VIC 3000, Australia

**Keywords:** alternative splicing, proteomics, RNA

## Abstract

Alternative splicing is a critical determinant of genome complexity and, by implication, is assumed to engender proteomic diversity. This notion has not been experimentally tested in a targeted, quantitative manner. Here, we have developed an integrative approach to ask whether perturbations in mRNA splicing patterns alter the composition of the proteome. We integrate RNA sequencing (RNA-seq) (to comprehensively report intron retention, differential transcript usage, and gene expression) with a data-independent acquisition (DIA) method, SWATH-MS (sequential window acquisition of all theoretical spectra-mass spectrometry), to capture an unbiased, quantitative snapshot of the impact of constitutive and alternative splicing events on the proteome. Whereas intron retention is accompanied by decreased protein abundance, alterations in differential transcript usage and gene expression alter protein abundance proportionate to transcript levels. Our findings illustrate how RNA splicing links isoform expression in the human transcriptome with proteomic diversity and provides a foundation for studying perturbations associated with human diseases.

## Highlights

• Integrative approach to study contribution of alternative splicing to proteome
• Changes in isoform usage alter protein abundance proportionate to transcript levels
• Intron retention is accompanied by decreased protein abundance
• Differential gene expression functionally tunes the human proteome

## Graphical Abstract

**Figure:**
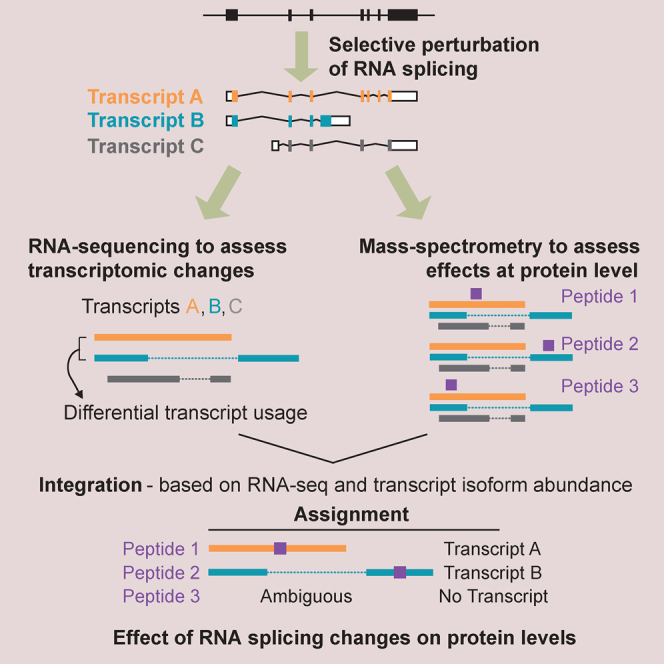

## Teaser

Liu et al. have developed an integrative approach to ask whether perturbations in mRNA splicing patterns alter the composition of the proteome. Their findings illustrate how RNA splicing links isoform expression in the human transcriptome with proteomic diversity and provides a foundation for studying perturbations associated with human diseases.

## Introduction

Next-generation RNA sequencing (RNA-seq) has identified alternative splicing of RNA transcripts as a key mechanism that may underlie the diversification of proteins encoded in the human genome. Such diversification may be essential for biologic complexity, because the number of protein-coding human genes is lower than was widely predicted before the genome sequence was known (Kim et al., 2014, Lander et al., 2001). Transcripts from ∼95% of multi-exon human genes are alternatively spliced (Pan et al., 2008, Wang et al., 2008). However, the extent to which this increased genomic complexity contributes to the generation of proteomic diversity is largely unknown. Initial efforts to assess the contribution of alternative splicing to proteomic composition and diversity have focused exclusively on the identification of proteins derived from alternatively spliced transcripts in a steady-state system (Blakeley et al., 2010, Brosch et al., 2011, Ezkurdia et al., 2012, Lander et al., 2001, Leoni et al., 2011, Tress et al., 2008, Xing et al., 2011, Zhou et al., 2010). More recent studies have incorporated expression data, such as evidence from RNA-seq experiments, in the interrogation of proteomic datasets to reduce mapping noise (Lopez-Casado et al., 2012, Ning and Nesvizhskii, 2010, Sheynkman et al., 2013, Tanner et al., 2007). However, none of these studies have attempted to quantify the contribution of alternative splicing to proteomic diversity in a systematic manner. Here, we seek to address this fundamental biological question by asking whether selective perturbations in RNA splicing patterns manifest as changes in the composition of the proteome. By using this system, we have established in a quantitative manner how changes in splicing of a subset of transcripts determine differential protein expression.

## Results and Discussion

### Experimental Strategy to Study Alternative Splicing at the Proteomic Level

**Figure 1:**
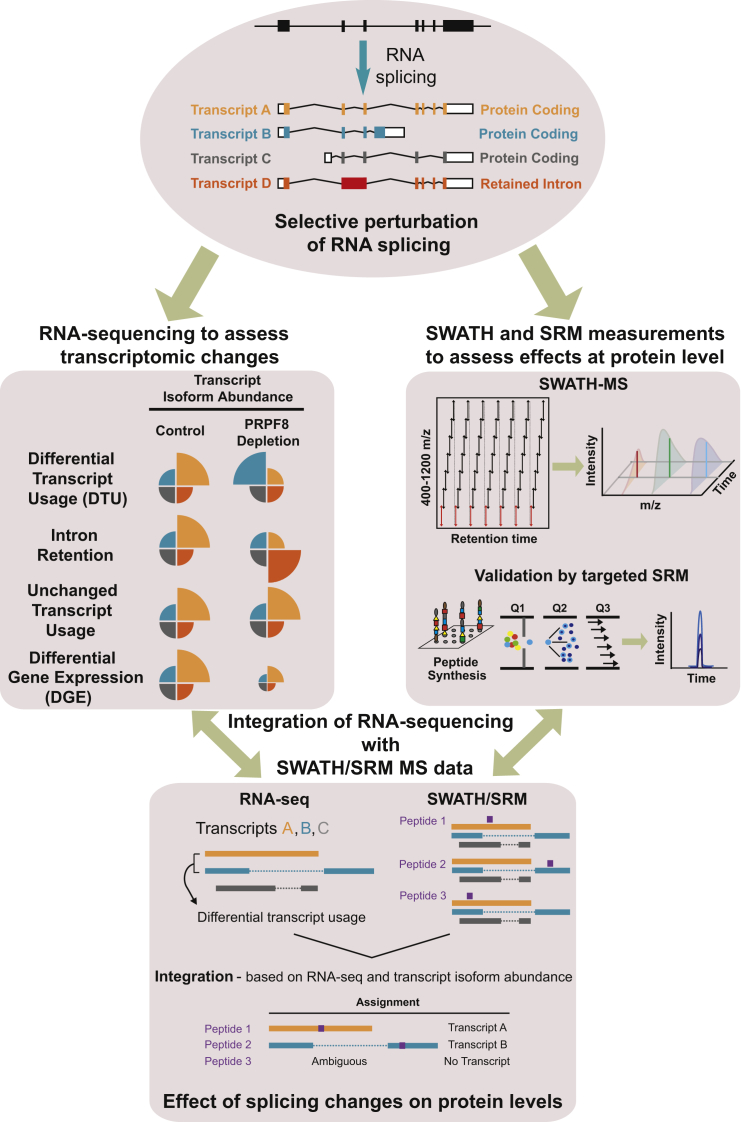
Framework to Study Contribution of Alternative Splicing to Proteomic Composition and Diversity Experimental and analysis workflow. Top: RNA splicing can result in generation of multiple transcripts as indicated in this hypothetical example, including different protein coding transcripts (transcripts A–C), as well as transcripts with retained introns (transcript D). Protein coding exons are represented by solid color bars, 5′ and 3′ untranslated regions are represented by white boxes, introns are represented by black lines, and a retained intron is represented by a dark red bar. We selectively perturbed RNA splicing by depleting the core spliceosome factor PRPF8 and used RNA-seq to assess the transcriptomic changes (left) and mass spectrometry to assess the effects at the protein level (right). PRPF8 depletion can alter the relative transcript abundance of the 4 transcripts, resulting in differential transcript usage (DTU), intron retention, or unchanged transcript usage. We have defined DTU to include cases where there is a change in transcript relative abundance between conditions. Differential gene expression (DGE) may also result, where the relative transcript abundances are unchanged between conditions, but changes in expression at the gene level are observed. We used SWATH-MS (sequential window acquisition of all theoretical spectra) to assess the effects at the protein level, which were validated by targeted SRM (selective reaction monitoring). We integrated the complete proteomic dataset based on knowledge from our RNA-seq experiments in order to guide the peptide assignments (bottom panel). Because peptide 2 maps uniquely to transcript B, it is assigned to transcript B. Peptide 1 maps to multiple transcripts in the same gene (A and C), but after PRPF8 depletion, the expression of only one of these transcripts is changed. The change affects the dominant expressed isoform for this gene (known as a major transcript), hence, peptide 1 is assigned to transcript A. In contrast, peptide 3 maps simultaneously to multiple differentially used transcripts and is therefore considered ambiguous, precluding assignment to any transcript.

We selectively perturbed RNA splicing by depleting the core spliceosome U5 small nuclear ribonucleo protein (snRNP) component PRPF8 and assessed subsequent transcriptomic and proteomic changes by RNA-seq and SWATH-MS (sequential window acquisition of all theoretical spectra-mass spectrometry), respectively (Figure 1). This is a compelling system because a number of studies have demonstrated the regulatory potential of the core spliceosome machinery (Clark et al., 2002, Papasaikas et al., 2015, Park et al., 2004, Pleiss et al., 2007, Saltzman et al., 2011, Wickramasinghe et al., 2015). Furthermore, we have extensively experimentally validated this system for studying splicing at the mRNA level (Wickramasinghe et al., 2015). Thus, using DEXSeq (Anders et al., 2012), we previously identified 3,370 transcripts with altered splicing patterns after PRPF8 depletion (1,284 with differential exon usage, 1,449 with intron retention, 637 with both), which constitute only a subset of all expressed protein-coding genes (13,216 genes; expression threshold = 1 fragments per kilobase million [FPKM]) (Wickramasinghe et al., 2015). To enable the quantification of a large fraction of the proteome with high accuracy, we used a recently developed data-independent acquisition (DIA) method, SWATH mass spectrometry (SWATH-MS), which combines the comprehensive proteome coverage of conventional shotgun proteomics with the high reproducibility and quantitative accuracy of targeted protein profiling based on SRM (selective reaction monitoring) (Gillet et al., 2012, Liu et al., 2013, Röst et al., 2014). Using SWATH-MS and the OpenSWATH software (Röst et al., 2014), we were able to identify and quantify 14,695 peptides (false discovery rate [FDR] 1%) across three biological replicates for each condition that uniquely map to 2,805 protein-encoding Ensembl genes. SWATH-MS yielded high reproducibility between technical (averaged Pearson correlation coefficient R = 0.99) and biological replicates (averaged R = 0.94) (Figure S1A). Collectively, 1,542 proteins display at least one peptide with altered protein expression levels after PRPF8 depletion (Figure S1B). Functional annotation revealed that transcripts with altered splicing patterns and proteins with altered levels are enriched in the same functional categories, namely translation, RNA splicing, mitotic cell cycle, and ubiquitination (Figure S1C). In contrast, proteins with unchanged levels after PRPF8 depletion are not enriched in these categories and are instead enriched for those involved in transcription and ribosome biogenesis (Figure S1D). Thus, significant alternative splicing events captured at the transcriptome level are functionally mirrored at the proteomic level.

### Establishing Methods to Integrate RNA-Seq with SWATH/SRM Mass Spectrometry

To integrate the transcriptomic and proteomic datasets, we focused on identifying differential splicing events at the transcript level, which represents a major computational challenge (Kitchen et al., 2014, Low et al., 2013, Vogel and Marcotte, 2012). Previous analyses, including our own (Figure S1), have identified differential splicing events from an exon-centric perspective through mapping to the genome using DEXSeq (Anders et al., 2012). However, this approach is limited given that differential exon usage provides no information on transcript expression levels, which is expected to influence protein expression. Furthermore, differentially used exons may map to many transcripts from the same gene, making peptide assignment difficult. To overcome these limitations, we explored a transcript-centric approach, which considers transcripts as whole units, to facilitate the integration with the proteomic dataset. We first estimated transcript expression levels with MMSEQ (Turro et al., 2011) and then used its companion tool MMDIFF (Turro et al., 2014) to identify both differentially expressed genes and differentially used transcripts. Genes with differential transcript usage (DTU) are defined as cases where there is a change in the transcript relative abundances between conditions (see pie-charts in Figure 1, left panel). We identified, at high confidence, 388 genes that display DTU and 2,021 genes that display differential gene expression (DGE) following depletion of PRPF8 (Tables 1 and S3). Transcript levels of differently used transcripts were validated by RT-PCR (see Figure 5 and Wickramasinghe et al., 2015 for differently used mitotic transcripts) and genes with differential expression were validated by qRT-PCR (see Figure S5C).

**Table 1:** Alternative Integration Strategies for Differently Used Transcripts and Peptides Detected by SWATH Mass Spectrometry

Transcript Set (DTU All No.)	Peptide Set (No.)	Initial Overlap		After Assignment	Correlation Coefficient (ρ)	Agreement (%)
**DTU All Transcripts and Uniquely Mapping Peptides**						

		transcript	30	30	ρ 0.487	Y, 21 (70)
transcripts (452)	peptides (2,974)	peptides	65	65	p value 0.01688	N, 9 (30)
genes (388)	genes (859)	genes	30	30		

**DTU All Transcripts and All Peptides**						

		transcript	158	118	ρ 0.274	Y, 68 (57.63)
transcripts (452)	peptides (14,695)	peptides	700	530	p value 0.00378	N, 50 (42.37)
genes (388)	genes (2,805)	genes	128	116		

**DTU Major Transcripts and Uniquely Mapping Peptides**						

		transcript	27	27	ρ 0.498	Y, 20 (74.07)
transcripts (291)	peptides (2,974)	peptides	61	61	p value 0.01672	N, 7 (25.93)
genes (263)	genes (859)	genes	27	27		

**DTU Major Transcripts and All Peptides**						

		transcript	97	77	ρ 0.486	Y, 56 (72.73)
transcripts (291)	peptides (14,695)	peptides	481	419	p value 1.97E−05	N, 21 (27.27)
genes (263)	genes (2,805)	genes	84	75		

**Figure 2:**
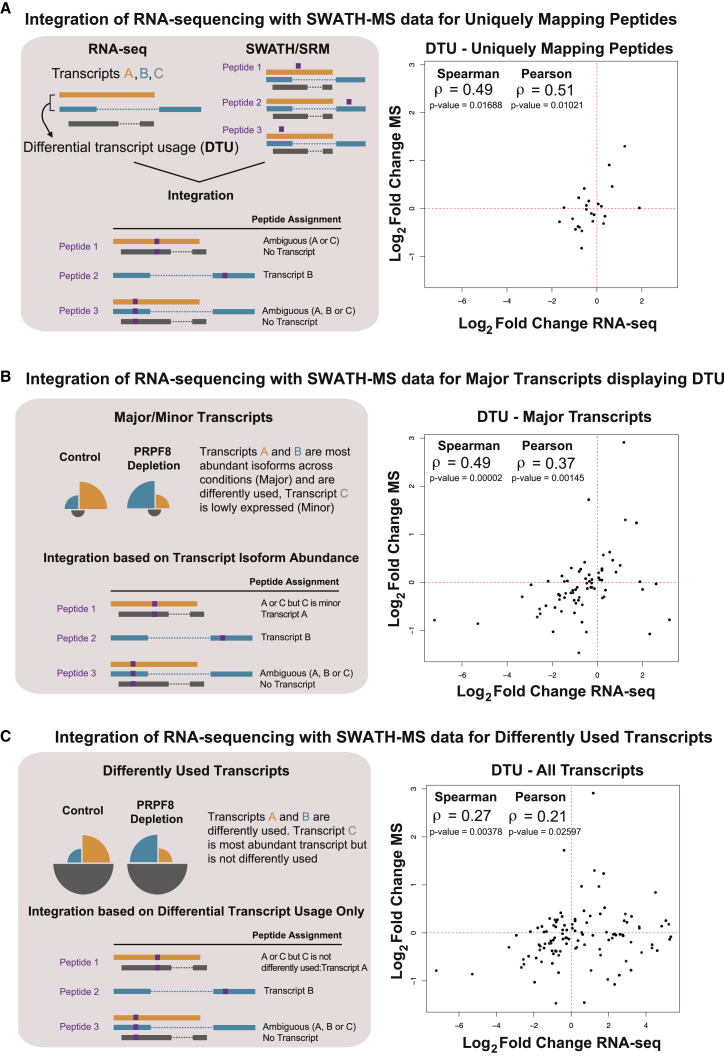
Changes in Isoform Usage Manifest Themselves at the Proteome Level (A) Uniquely mapping peptides and SWATH-MS. Schematic indicating peptide to transcript mapping for uniquely mapping peptides is shown on left. Scatterplot comparing changes in expression of differently used transcripts (DTU) (log_2_ fold change RNA-seq) to changes in expression of the peptides that uniquely map to them (log_2_ fold change SWATH-MS) after PRPF8 depletion is shown on right. Spearman and Pearson correlation coefficients and associated p values are shown in top left corner. (B) Major transcripts and SWATH-MS. Schematic indicating peptide to transcript mapping for major transcripts is shown on left. Similar scatterplot is shown on right for transcripts whose most highly expressed isoform (major transcript) changes in expression after PRPF8 depletion with corresponding peptide evidence. (C) Use of an alternative integration strategy for peptide assignment where information about transcript expression levels was not considered increases dataset size but reduces correlation coefficient. Specifically, if a peptide maps to multiple transcripts in the same gene, but the expression of only one of these transcripts was changed after PRPF8 depletion, then this peptide was assigned to that particular transcript regardless of its expression level. In contrast, peptides that map simultaneously to multiple differentially used transcripts were considered ambiguous and were not used for further analysis. A scatterplot comparing changes in expression of differently used transcripts (DTU) (log_2_ fold change RNA-seq) to changes in expression of their corresponding peptides (log_2_ fold change SWATH-MS) after PRPF8 depletion is shown. Spearman and Pearson correlations coefficient and associated p values are shown in top left corner. See also Figure S2 and Table 1.

We first considered the set of peptides that map uniquely to alternatively spliced transcripts involved in DTU events, defined from the mRNA data. In other words, peptide expression levels can be directly and exclusively associated with the transcripts of interest. Using this approach, we evaluated the impact at the protein level of the changes in splicing detected by RNA-seq experiments, based on the correlation between fold changes in transcript and peptide expression after PRPF8 depletion. RNA-seq fold changes were calculated from the transcript-level expression estimates obtained from MMSEQ. For each transcript, the fold change represents the median transcript expression in PRPF8-depleted versus control small interfering RNA (siRNA)-treated samples across 3 biological replicates. Peptide fold changes for each transcript were calculated by first adding up the intensities of all the peptides that mapped to that transcript in each given biological replicate and then dividing the median sum for PRPF8 depletion versus controls (hence resulting in one fold change per transcript). We observe a Spearman’s correlation coefficient of 0.49 and a Pearson correlation coefficient of 0.51 when comparing fold changes in RNA and protein expression (65 peptides from 30 genes; p value = 0.0169, Spearman; p value = 0.0102, Pearson; correlation test) (Figure 2A; Table 1). Use of an alternative strategy to determine peptide fold changes for each transcript, whereby the fold change for PRPF8 depletion versus controls was determined individually for each peptide to obtain the median fold change of all peptides that mapped to that transcript, yielded similar results (see Table S2). However, uniquely mapping peptides represent a minority of cases (2,974 out of 14,665 peptides detected by SWATH-MS), because many peptides, due to their length yielded from using routine trypsin digestion and the detection range of biological mass spectrometry, are shared between transcript isoforms.

### Integration of Complete SWATH Proteomic Dataset

**Figure 3:**
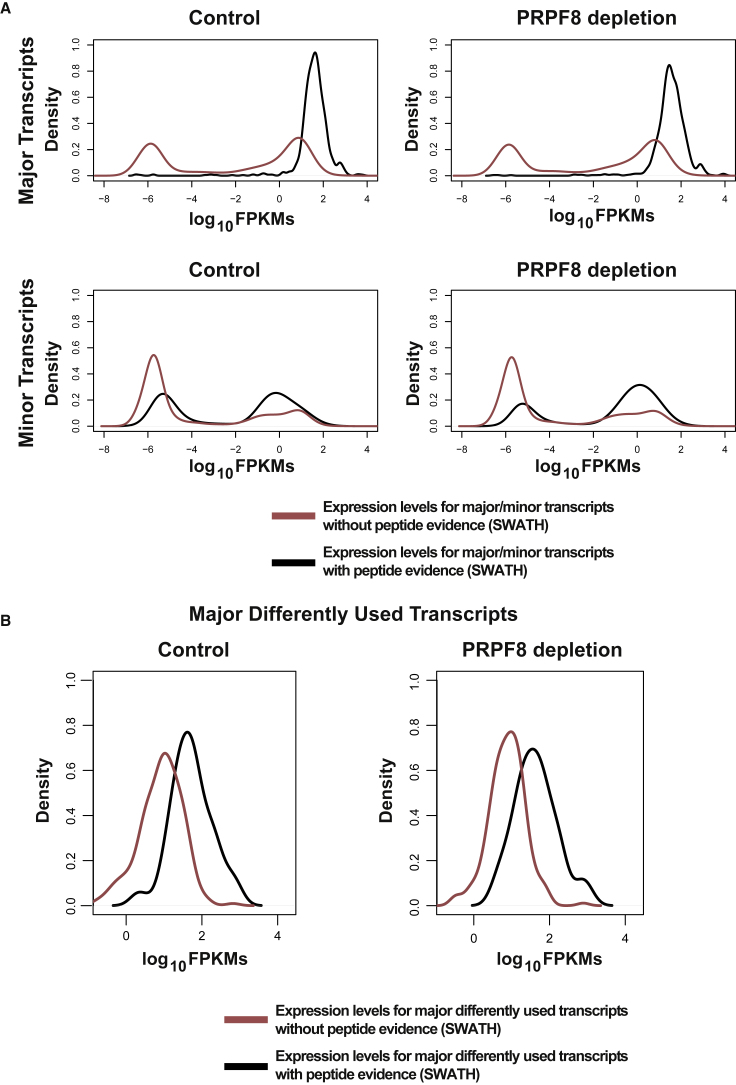
Peptides Encoded by Major Transcripts Are More Frequently Detected by SWATH-MS (A) Expression levels (log_10_ FPKMs [fragments per kilobase of transcript per million mapped reads]) of major and minor transcripts with or without peptide evidence are indicated for Control and PRPF8-depleted samples. Only uniquely mapping peptides were used for this analysis and one biological replicate for each condition is shown. (B) Lowly expressed major transcripts displaying DTU are not detectable as expressed protein product within dynamic range of SWATH mass spectrometry. Expression levels (log_10_ FPKMs) for major transcripts displaying DTU with or without peptide evidence are indicated for Control and PRPF8-depleted samples. One biological replicate for each condition is shown.

To integrate the complete proteomic dataset, we devised a strategy that takes advantage not only of the information from peptides that map uniquely to one transcript isoform, but also from those that map to several transcripts of the same gene (Figure 2B). To do this, we used information from our RNA-seq experiments to guide the peptide assignments. More specifically, for genes with multiple isoforms, many are expressed at an extremely low level in comparison to the most abundant isoforms (Gonzàlez-Porta et al., 2013) (Figure 3A). Such a low level of mRNA expression is unlikely to manifest itself as expressed protein product within the dynamic range of the mass spectrometric method used that is ∼4.4 orders of magnitude (Figure 3A). Consequently, for each gene, we considered only the most highly expressed transcript in each condition (major transcript) for peptide assignment, discarding cases where DTU did not arise in one of these. Using this criterion, we identified 263 genes whose major transcript displayed DTU (Table 1). In some cases, the identity of the major transcript differs between conditions (as discussed below), whereupon we determined separately for each major transcript whether there was evidence of differential usage following depletion of PRPF8. Subsequently, we used the regions that distinguished these two transcripts to uniquely allocate peptides (Figure 2B).

This approach yields peptide fold change information for 419 peptides corresponding to 75 genes that display DTU. Comparing mRNA fold changes with protein expression using this dataset yields a Spearman’s correlation coefficient of 0.49 and a Pearson correlation coefficient of 0.37 (Figure 2B; p value = 1.97 × E−05, Spearman; p value = 0.0015, Pearson; correlation test) (Table 1). Importantly, this correlation coefficient is broadly similar to that obtained with uniquely mapping peptides across all isoforms, but using a significantly larger dataset (419 peptides from 75 genes versus 65 peptides from 30 genes). Nevertheless, most weakly expressed major transcripts displaying DTU are undetectable as expressed protein product within the dynamic range of mass spectrometry (Figure 3B). When we focus on major transcripts and uniquely mapping peptides, we observe a Spearman’s correlation coefficient of 0.50 and a Pearson correlation coefficient of 0.52 (61 peptides from 27 genes; p value = 0.0167, Spearman; p value = 0.0117, Pearson; correlation test) (Figure S2). In contrast, use of an alternative integration strategy that assigns peptides to all differently used transcripts regardless of their expression levels resulted in an increase in the dataset size (530 peptides corresponding to 116 genes that display DTU) but a sharp decrease in Spearman’s correlation coefficient to 0.27 and to 0.21 for Pearson (p value = 0.0378, Spearman; p value = 0.0260, Pearson; correlation test) (Figure 2C; Table 1). This result suggests that the inclusion of minor transcripts with low expression levels increases noise at both the mRNA and protein level, making reliable peptide assignment difficult (Figure 3). Consequently, this indicates that usable information can be obtained from peptides that map to more than one transcript in the same gene only if information on transcript abundance is considered. Taken together, these results suggest that transcript expression levels play a dominant role in regulating protein abundance, which supports the idea that differential splicing events in minor transcripts correspond to subtle changes that do not have a strong impact on the overall proteome, whatever their functional outcome.

### Validation Using Selective Reaction Monitoring Mass Spectrometry

**Figure 4:**
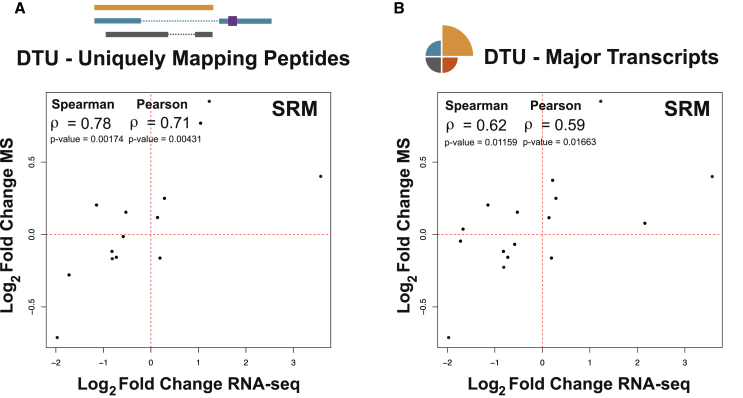
Validation Using Selective Reaction Monitoring Mass Spectrometry (SRM) (A) Uniquely mapping peptides and SRM. Scatterplot comparing changes in expression of differently used transcripts (DTU) (log_2_ fold change RNA-seq) to changes in expression of the peptides that uniquely map to them (log_2_ fold change SRM-MS) after PRPF8 depletion. Peptide expression information was obtained using SRM. (B) Major transcripts and SRM. Similar scatterplot is shown for transcripts whose most highly expressed isoform (major transcript) changes in expression after PRPF8 depletion with corresponding peptide evidence. See also Figure S3C and Table S2.

To validate our findings using a more sensitive mass spectrometric approach, we performed selective reaction monitoring (SRM) on control siRNA-treated and PRPF8-depleted samples (Figures S3A and S3B). To increase the quantitative precision, we spiked heavy isotope-labeled peptide standards for SRM measurement into the sample. SRM has a higher sensitivity but a much lower analyte throughput than SWATH; hence, we were only able to determine peptide fold change information for 53 targeted peptides corresponding to 15 genes whose major transcripts display DTU. Comparing mRNA fold changes with protein expression using this dataset yields a Spearman’s correlation coefficient of 0.62 and a Pearson correlation coefficient of 0.59 (p value = 0.0116, Spearman; p value = 0.01663, Pearson; correlation test) (Figure 4B; Table S1). When considering only peptides that map uniquely to transcripts involved in DTU events, we observe an increased correlation coefficient of 0.78 (0.71 for Pearson) (35 peptides from 13 genes; p value = 0.0017, Spearman; p value = 0.0043, Pearson; correlation test) (Figure 4A; Table S1) and 0.73 (0.70 for Pearson) when focusing on major transcripts and uniquely mapping peptides (33 peptides from 12 genes; p value = 0.0063, Spearman; p value = 0.00794, Pearson; correlation test) (Figure S3C; Table S1). Collectively, our findings demonstrate that changes in isoform usage across the human transcriptome manifest at the proteome level.

### Biological Impact of Functional mRNA Isoforms through Proteome Diversity

**Figure 5:**
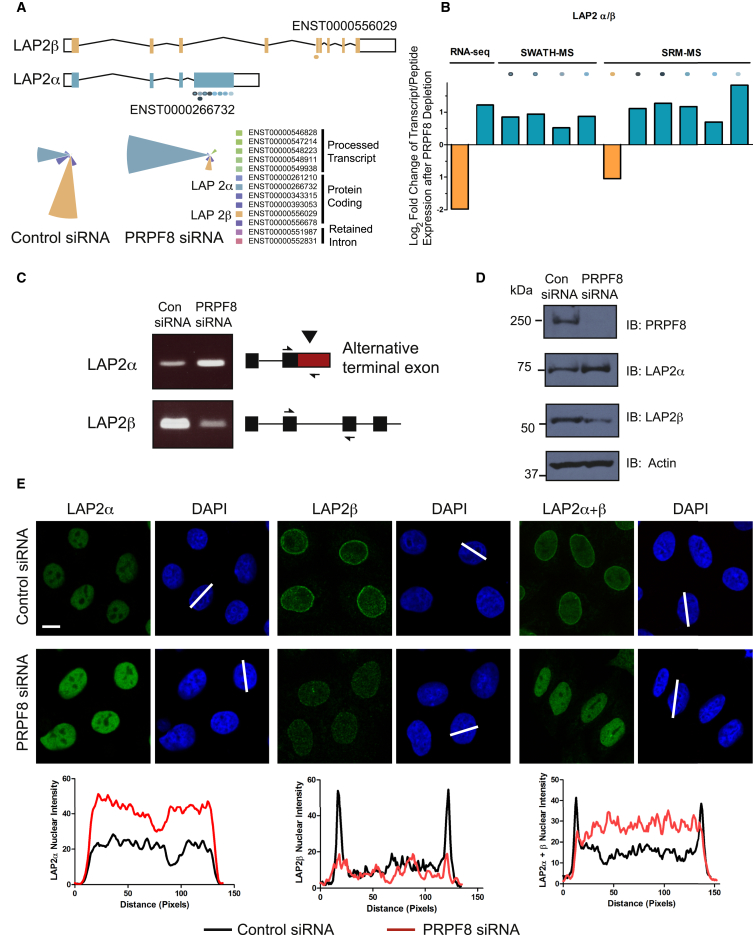
Biological Impact of Functional mRNA Isoforms (A) Transcript representation of LAP2 isoforms and starplots of transcript relative abundance in control siRNA-treated and PRPF8-depleted cells. One biological replicate is shown. (B) LAP2 switch event with corresponding peptide evidence. Column plots show fold change in expression of transcripts (left two columns) and peptides (right columns) after PRPF8 depletion. A negative fold change is represented in yellow (LAP2β), and a positive fold change in turquoise (LAP2α). Each peptide detected by SWATH or SRM is shown individually, and the region of the transcript to which it maps is represented in (A) by different colored ovals. (C and D) Changes in LAP2 isoform expression are confirmed at the RNA (C) and protein (D) levels using probes and antibodies that recognize each specific isoform. (E) LAP2 isoform localization is altered after PRPF8 depletion. Immunofluorescence of LAP2β and LAP2α isoforms is shown in control siRNA-treated and PRPF8-depleted Cal51 cells using antibodies that recognize each specific isoform and both isoforms respectively (scale bar, 5 μm). Scanning analysis of LAP2 isoform intensity is also shown with the scanning axes indicated by white lines. Pairs of nuclei of same scan width as determined by DAPI were used for scanning using ImageJ (NIH). Experiments in (C)–(E) were replicated independently 3 times and one representative experiment is shown. See also Figures S4 and S5.

Alternative splicing has the potential to vastly increase the diversity of proteins encoded by the human genome. To assess whether the changes in alternative splicing that we observe at the protein level may have a functional impact on cellular biology, we focused on extreme examples of alternative splicing, where the identity of major transcripts changes across conditions. This is termed a switch event (Gonzàlez-Porta et al., 2013) and two examples that result in changes in protein isoform expression as determined by SWATH and SRM are shown in Figures 5 and S4 (LAP2 and hnRNPK). We focused on lamin-associated polypeptide (LAP2), also known as thymopoietin, because its various isoforms have been functionally characterized in detail (Dechat et al., 1998, Somech et al., 2005). LAP2 undergoes a switch event after PRPF8 depletion, whereby the dominant isoform changes from LAP2β to LAP2α, whose N-terminal region of 187 amino acids (encoded by exons 1–3) is shared with LAP2β (Figures 5A and S5A). Changes in protein expression of each isoform, consistent with the change observed at the mRNA level, were determined by SWATH-MS and validated by SRM. Thus, one peptide shared by both isoforms did not change in expression after PRPF8 depletion, whereas peptides uniquely mapping to LAP2β decreased and those to LAP2α increased respectively (Figures 5B and S5B). These changes were confirmed at the RNA (Figure 5C) and protein (Figure 5D) levels using probes and antibodies that recognize each specific isoform.

Both isoforms have different cellular locations and functions: LAP2β localizes to the nuclear lamina and represses transcription of p53 and nuclear factor κB (NF-κB) target genes (Dechat et al., 1998, Somech et al., 2005), while LAP2α is localized throughout the nuclear interior and is implicated in the structural organization of the nucleus (Dechat et al., 1998). In unperturbed cells, the majority of LAP2 protein localizes to the nuclear lamina, corresponding to the LAP2β isoform (Figure 5E). Following PRPF8 depletion, this staining pattern is reversed: less LAP2 protein is observed at the nuclear lamina, and more is observed in the nuclear interior, corresponding to increased levels of the LAP2α isoform (Figure 5E). Consistent with a reduction in LAP2β levels, we observe a de-repression of direct p53 and NF-κB transcriptional targets after PRPF8 depletion (Figure S5C), highlighting the potential biological impact of functionally relevant mRNA isoforms by the quantitative modulation of their respective protein isoforms.

### Intron Retention Reduces Protein Levels

**Figure 6:**
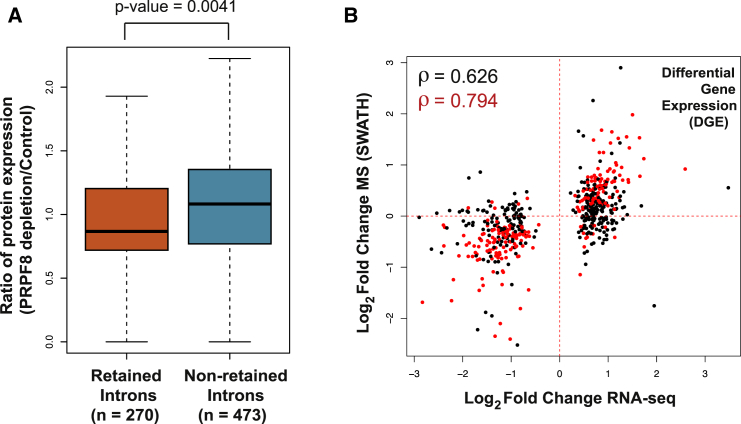
Intron Retention and Differential Gene Expression Functionally Tune the Human Proteome (A) Intron retention reduces protein levels. Boxplot representing the ratio of protein expression (PRPF8 depletion/Control) is shown for retained introns (n = 270) and non-retained introns (n = 473) with peptide evidence. p value is indicated (Wilcoxon test). (B) Alterations in gene expression alter protein abundance proportionally to transcript levels. Scatterplot comparing changes in expression of differentially expressed genes (DGE) (log_2_ fold change RNA-seq) to changes in expression of the peptides that map to them (log_2_ fold change SWATH-MS) after PRPF8 depletion. Spearman’s correlation coefficient is shown in top left corner. Differently expressed genes whose corresponding peptides change significantly in expression (adjusted p value < 0.1, t test, Holm method) are indicated in red and associated correlation coefficient is also shown in red. See also Figure S6 and Table S3.

Intron retention is a specific form of alternative splicing that is increasingly regarded as a regulatory event that can control gene expression (Kalyna et al., 2012, Wong et al., 2013, Yap et al., 2012). We therefore assessed the impact of intron retention on the composition of the proteome. Recent findings have suggested that intron retention affects transcripts from as many as 75% of multi-exon genes (Braunschweig et al., 2014). Transcripts with retained introns may not be translated because they are retained in the nucleus as they are not competent for export or may contain a premature termination codon (PTC) that results in their degradation by the nonsense-mediated decay (NMD) pathway. Consistent with this hypothesis, intron retention leading to NMD has a significant impact on transcript levels (Braunschweig et al., 2014). However, the effect of retained introns on protein expression has not been examined to date using a systematic approach covering the transcriptome. Following PRPF8 depletion, we see an increase in the expression levels of intronic reads throughout the genome (p value < 2.2 × 10^−16^) (Wickramasinghe et al., 2015). We obtained peptide evidence for 270 genes that display retained introns (identified using DEXSeq [Anders et al., 2012], see the Experimental Procedures) following PRPF8 depletion and asked whether protein expression is downregulated in these genes compared to those without intron retention. We find that the expression of their encoded proteins is reduced in comparison to those that do not display retained introns (n = 473, p value = 0.0041, Wilcoxon test) (Figure 6A). Furthermore, the proportion of downregulated proteins is higher in the group of genes with retained introns compared to those without intron retention (161/270 versus 231/473; p value = 0.0048, odds ratio: 1.547).

The relative abundance of protein-coding transcripts for each gene also has a significant effect on protein expression. Indeed, when considering genes with at least one transcript displaying a retained intron biotype, the encoded proteins that are downregulated after PRPF8 depletion have a higher relative abundance of transcripts that are not protein-coding (i.e., display intron retention) for each gene in comparison to those whose proteins are upregulated (p value = 0.0098) (Figure S6A). One example is shown in Figures S4C and S4D, where the dominant protein-coding isoform downregulated after PRPF8 depletion is replaced by an isoform with a retained intron, resulting in a decrease in protein expression (according to 5 peptides detected by SWATH-MS with average PRPF8 depletion/control fold change of −0.575, p value = 0.018). Interestingly, for some genes with intron retention after PRPF8 depletion, their corresponding protein expression levels are unchanged or even increased. This suggests a complex model whereby compensation mechanisms may be at play. However, the mere detection of a transcript with a retained intron bio-type by RNA-seq may not affect the levels of the protein encoded by that gene, unless it is expressed at a robust level. Indeed, for those proteins that are upregulated after PRPF8 depletion, the median protein coding transcript relative abundance is >0.9 (Figure S6). In other words, <10% of the transcripts that make up that gene display intron retention, which may explain why these transcripts have no effect on protein level. Collectively, these results suggest that intron retention functionally tunes the human proteome as well as the transcriptome.

### Alterations in Gene Expression Alter Protein Abundance Proportionally to Transcript Levels

Protein abundance is a direct determinant of cellular function and is heavily influenced by transcript levels. However, the quantitative contribution of mRNA abundance to protein abundance remains controversial (Cheng et al., 2016, Jovanovic et al., 2015, Kristensen et al., 2013, Li et al., 2014, Liu et al., 2016, Robles et al., 2014, Schwanhäusser et al., 2011, Vogel et al., 2010, Vogel and Marcotte, 2012). Given that steady-state mRNA and protein abundance are controlled by a number of post-transcriptional and translational regulatory processes (Vogel and Marcotte, 2012), establishing a correlation between the transcriptome and the proteome is not straightforward. A number of studies have used advances in next-generation sequencing and proteomics to examine the correlation between mRNA and protein abundances under steady-state conditions. Generally, the results have indicated that although there is a strong correlation between mRNA and protein abundance, a substantial proportion of the variation in protein abundance cannot be attributed to mRNA expression alone (Fu et al., 2009, Ghazalpour et al., 2011, Lundberg et al., 2010, Nagaraj et al., 2011, Schwanhäusser et al., 2011). This may be due to technical or experimental noise, along with limitations of the timescale in the experimental design and data modeling approaches (Liu et al., 2016). In contrast, more recent studies using advanced technical measurements in both steady-state and perturbed conditions have suggested that changes in mRNA abundance play a dominant role in determining the majority of dynamic changes in protein levels (Jovanovic et al., 2015, Robles et al., 2014), although this may depend on the respective contributions of mRNA and protein level regulation to the biological system being studied (Cheng et al., 2016).

We determined the contribution of changes in mRNA abundance to protein abundance using our biological system of perturbed RNA splicing. We observe 2,021 genes that are differentially expressed (DGE) after PRPF8 depletion and obtained fold change information for 3,057 peptides corresponding to 572 genes that display DGE (Table S3). We observed a Spearman’s correlation coefficient of 0.63 when comparing RNA and protein fold changes in expression (Figure 6B; Table S3) that increases to 0.79 when focusing on peptides with a significant fold change (adjusted p value < 0.1, t test, Holm method) (Figure 6B; Table S3). A correlation coefficient of 0.58 (increasing to 0.76 when considering peptides with significant fold change, adjusted p value <0.1, t test, Holm method) is observed when focusing on uniquely mapping peptides (Figure S6B). Importantly, when we focus on the genes that do not display DGE, we observe a correlation coefficient of 0.29 when comparing RNA and protein fold changes in expression (Figures S6C and S6D), suggesting that changes in gene expression are driving the changes in protein expression. Taken together, these results suggest that in a system with perturbed alternative splicing, a significant proportion of the variation in protein abundance can be chiefly attributed to changes in mRNA levels.

In summary, our results illustrate how RNA splicing links isoform expression in the human transcriptome with proteomic diversity. We further show that alternative splicing events causing intron retention are accompanied by decreased protein abundance, whereas alterations in differential transcript usage and gene expression alter protein abundance proportionally to transcript levels. The fraction of the whole proteome mass of a human cell represented by the number of proteins identified in our study is very high (>99.5%) (Beck et al., 2011), suggesting that the observed events are likely to be representative for the proteome.

Our integrative analysis using a perturbed system suggests that alternative splicing events significantly contribute to both proteomic composition and diversity in humans. While a recent study that used ribosome occupancy as an indicator of translation output and not protein levels, supports our conclusions (Weatheritt et al., 2016), the contribution of alternative splicing to proteomic complexity remains divisive (Blencowe, 2017, Tress et al., 2017a, Tress et al., 2017b). The increase in correlation coefficient between mRNA and protein levels that we observe from SWATH to SRM suggests that a significant proportion of the protein variance from our perturbed system can be explained by differences in isoform usage. Critically, this depends on both the sensitivity of the mass spectrometric method used and the identification of high confidence alternative splicing events at the transcript level.

The methods we have developed to integrate RNA-seq and quantitative SWATH and SRM mass spectrometry data to study splicing demonstrate that usable information can be obtained from peptides that map to more than one transcript in the same gene once information on transcript abundance is considered. They provide a foundation for future studies to examine the proteome-wide effects of altered RNA splicing associated with human diseases (Kurtovic-Kozaric et al., 2015, Quesada et al., 2011, Tanackovic et al., 2011, Yoshida et al., 2011).

## Experimental Procedures

### Analysis of RNA-Seq Data

The transcriptome of control siRNA-treated and PRPF8-depleted Cal51 cells was sequenced on an Illumina HiSeq2000 platform using 100 bp paired-end reads with poly(A)^+^RNA isolated from 3 and 4 independent experiments, respectively, as previously described (Wickramasinghe et al., 2015). Raw reads were directly mapped to the transcriptome with Bowtie v0.12.7 (Langmead et al., 2009), using Ensembl v66 as a reference (Flicek et al., 2012). Following the estimation of transcript expression levels with MMSEQ v1.0.7 (Turro et al., 2011), its companion tool MMDIFF (Turro et al., 2014) was used to identify both differentially expressed genes and differentially used transcripts as described in more detail in the Supplemental Experimental Procedures.

### Protein Extraction and In-Solution Digestion

The cell pellets from three independent depletion experiments (control siRNA and PRPF8-depleted) were lysed on ice by using a lysis buffer containing 8 M urea (EuroBio), 40 mM Tris-base (Sigma-Aldrich), 10 mM DTT (AppliChem), and complete protease inhibitor cocktail (Roche) as described in more detail in the Supplemental Experimental Procedures.

### Shotgun and SWATH-MS Measurement

The peptides digested from Cal51 lysate were all measured on an AB Sciex 5600 TripleTOF mass spectrometer operated in DDA mode. The same liquid chromatography-tandem mass spectrometry (LC-MS/MS) system used for DDA measurements was also used for SWATH analysis (Collins et al., 2013, Gillet et al., 2012, Liu et al., 2013) and is described in more detail in the Supplemental Experimental Procedures.

### Assignment of Peptides to Transcripts

An initial set of 16,779 peptides was detected across biological replicates for each condition (control siRNA and PRPF8-depleted samples) using SWATH-MS and mapped against all the protein coding transcripts annotated in Ensembl v66, including those with a nonsense-mediated decay biotype. Removal of peptides that mapped to more than one gene led to a set of 14,695 peptides (corresponding to 2,805 genes), which was used for downstream analysis. Peptides were assigned to specific transcripts as outlined in Figure 1. Peptides that map uniquely to each transcript represented a minority of events (2,974 peptides mapping to 859 genes). Peptides that map ubiquitously to several transcripts of the same gene were assigned based on knowledge from the RNA-seq experiments using the following criteria. Two alternative peptide assignment strategies were considered. One strategy incorporated information on transcript isoform abundance for each gene into our analysis, whereby only peptides that map to major transcripts were considered. Major transcripts are the dominant expressed isoform for each gene and those identified as major in either control siRNA-treated or PRPF8-depleted samples were used specifically for peptide assignment. Additionally, we considered an alternative assignment strategy where information about transcript expression levels was not considered. Specifically, if a peptide maps to multiple transcripts in the same gene, but the expression of only one of these transcripts was changed after PRPF8 depletion, then this peptide was assigned to that particular transcript regardless of its expression level. In contrast, peptides that map simultaneously to multiple differentially used transcripts were considered ambiguous and were not used for further analysis.

### Integration of Transcriptomic and Proteomic Data

To integrate transcriptomic and proteomic data, fold changes in transcript and peptide expression after PRPF8 depletion were obtained from RNA-seq and SWATH or SRM mass spectrometry experiments, respectively. RNA-seq fold changes were calculated from the transcript-level expression estimates obtained from MMSEQ as described above. For each transcript, the fold change represents the median transcript expression in PRPF8-depleted versus control siRNA-treated samples.

Raw peptide intensities were first quantile-normalized in order to enable comparison across samples. For each peptide, the observed intensities across the biological replicates in each condition were summarized by using the median, and a fold change was obtained by dividing the value obtained for PRPF8-depleted and control siRNA-treated samples. Peptide fold changes for each transcript were calculated by first adding up the intensities of all the peptides that mapped to that transcript in each given biological replicate and then dividing the median value of the summed peptide signals for PRPF8 depletion versus controls (hence resulting in one fold change per transcript). The same analysis was used for both SWATH and SRM datasets. Use of an alternative strategy to determine peptide fold changes for each transcript, whereby the fold change for PRPF8 depletion versus controls was determined individually for each peptide to obtain the median fold change of all peptides that mapped to that transcript, yielded similar results (see Table S2). The fold changes derived from these two technologies were integrated as described in Figure 1. Spearman correlation was used to evaluate the relationship between transcript and peptide fold changes, as previously suggested (Maier et al., 2009). We also used Pearson correlation as a comparison.

## Author Contributions

Y.L. performed all mass-spectrometry experiments and analyzed data with M.G.-P. and V.O.W. M.G.-P. developed informatics pipelines and analyzed data with S.S., Y.L., and V.O.W. J.C.M. contributed to the method development, wrote the paper, and supervised M.G.-P. with A.B. A.R.V. and R.A. supervised the study, analyzed data, and wrote the paper. V.O.W. conceived the study, performed, and analyzed experiments, and wrote the paper.

## Accession Numbers

The accession number for the raw data of mass spectrometry measurements (SWATH-MS and shotgun) together with the input spectral library and OpenSWATH results reported in this paper is ProteomeXchange Consortium (http://proteomecentral.proteomexchange.org): PXD003278. The accession number for the RNA sequencing data reported in this paper is ArrayExpress: E-MTAB-3021.

## Supplemental Information

Document S1. Supplemental Experimental Procedures, Figures S1–S6, and Tables S1–S3

Document S2. Article plus Supplemental Information
