# Supplementary material for: Impact of Alternative Splicing on the Human Proteome
Source: Cell Rep. 2017 Aug 1;20(5):1229–41. doi: 10.1016/j.celrep.2017.07.025 (PMC5554779; doi:10.1016/j.celrep.2017.07.025)
Supplement: Document S1. Supplemental Experimental Procedures, Figures S1–S6, and Tables S1–S3 [file mmc1.pdf]

Cell Reports, Volume *20*

Supplemental Information

Impact of Alternative Splicing

on the Human Proteome

Yansheng Liu, Mar Gonzàlez-Porta, Sergio Santos, Alvis Brazma, John C. Marioni, Ruedi Aebersold, Ashok R. Venkitaraman, and Vihandha O. Wickramasinghe

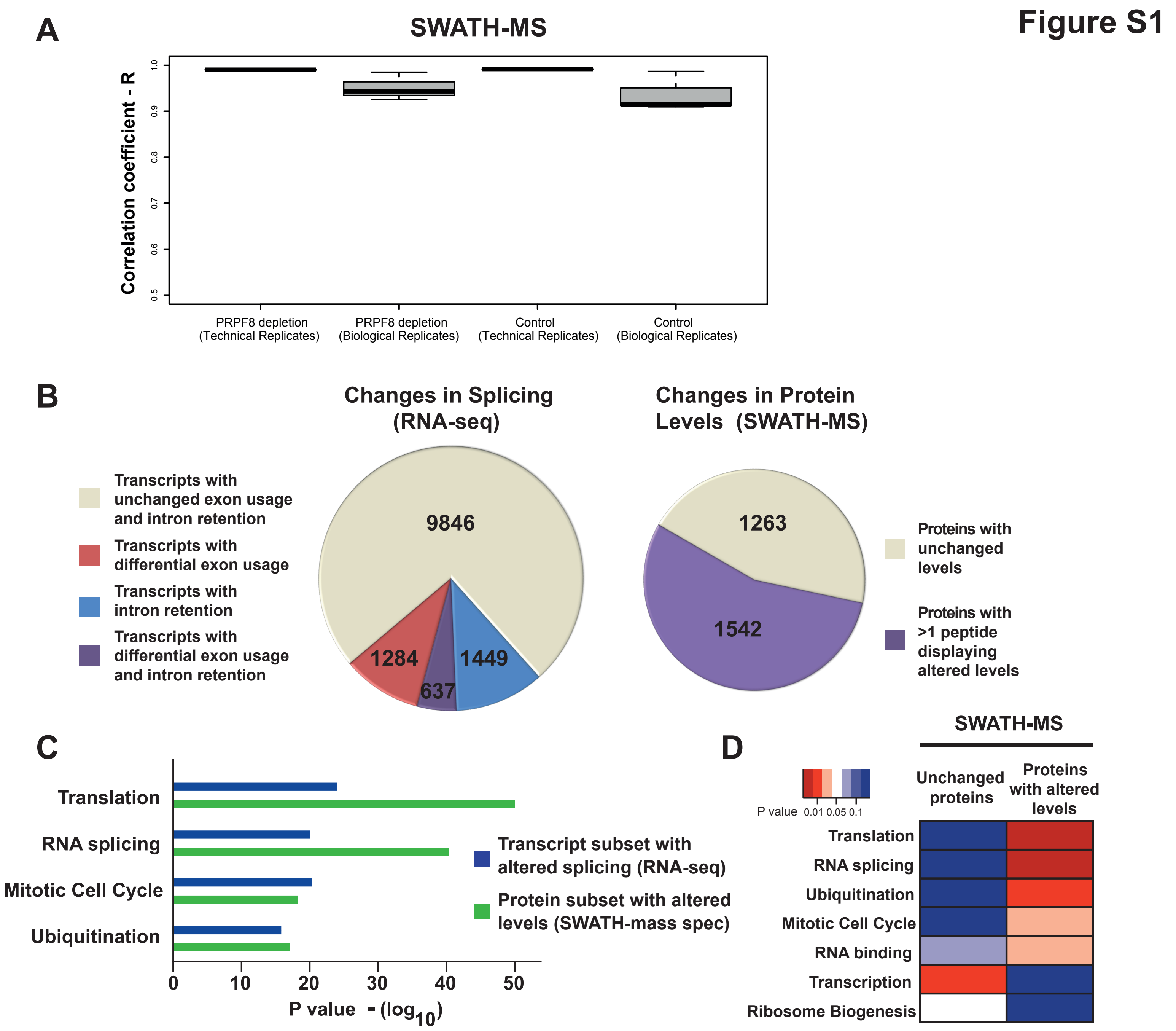

Figure S1, related to Figure 1: Transcripts with altered splicing patterns and proteins with altered levels are enriched in the same functional categories. **A**, Reproducibility of SWATH-MS data. The Pearson correlation coefficient between SWATH intensities identified and quantified from all the peptides from 3 technical replicates and 3 biological replicates for either Control or PRPF8 depleted samples analysed by SWATH-MS is indicated. **B**, Pie-chart representing proportion of transcripts with altered splicing patterns after PRPF8 depletion (differential exon usage, intron retention) as determined using DEX-seq is shown. Proportion of proteins detected by SWATH-MS with at least 1 peptide displaying altered levels after PRPF8 depletion is also indicated.
C, Functional enrichment analysis using DAVID shows that the transcript subset with altered splicing and the protein subset with altered levels are enriched for those that participate in translation, RNA splicing, mitotic cell cycle and ubiquitination. **D**, Functional enrichment analysis using DAVID with proteins detected by SWATH-MS as background shows that subset of proteins with unchanged levels after PRPF8 depletion are enriched for those involved in transcription and ribosome biogenesis. p-values are colour-coded.

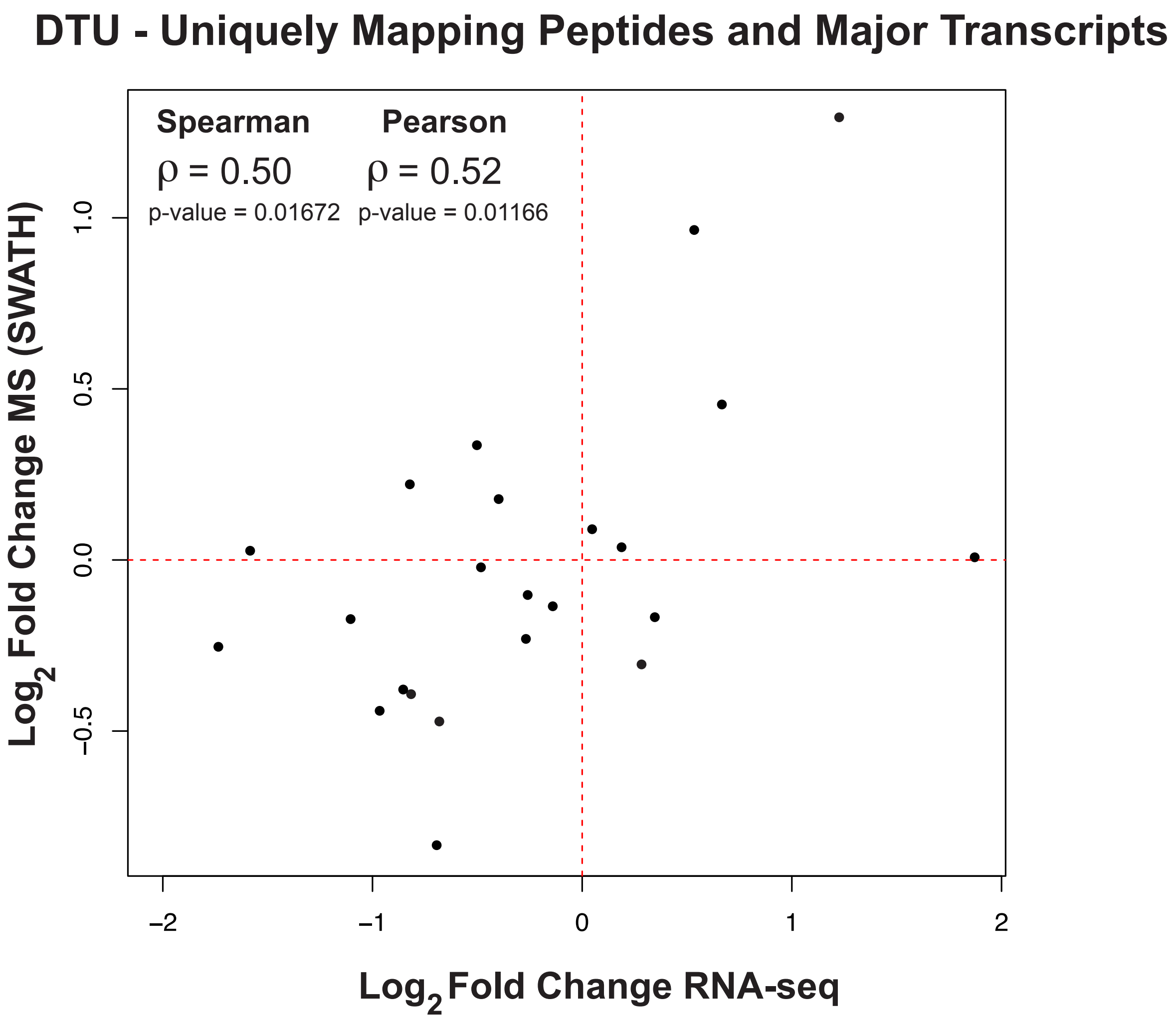

Figure S2, related to Figure 2: Correlation plot for major transcripts and uniquely mapping peptides using SWATH-MS. Scatterplot comparing changes in expression of differently used transcripts (DTU) whose most highly expressed isoform (major transcript) changes in expression (log2 fold change RNA-seq) to changes in expression of the peptides that uniquely map to them (log2 fold change SWATH-MS) after PRPF8 depletion. Spearman's correlation coefficient and p-value (correlation test) are shown in top left corner.

Figure S3

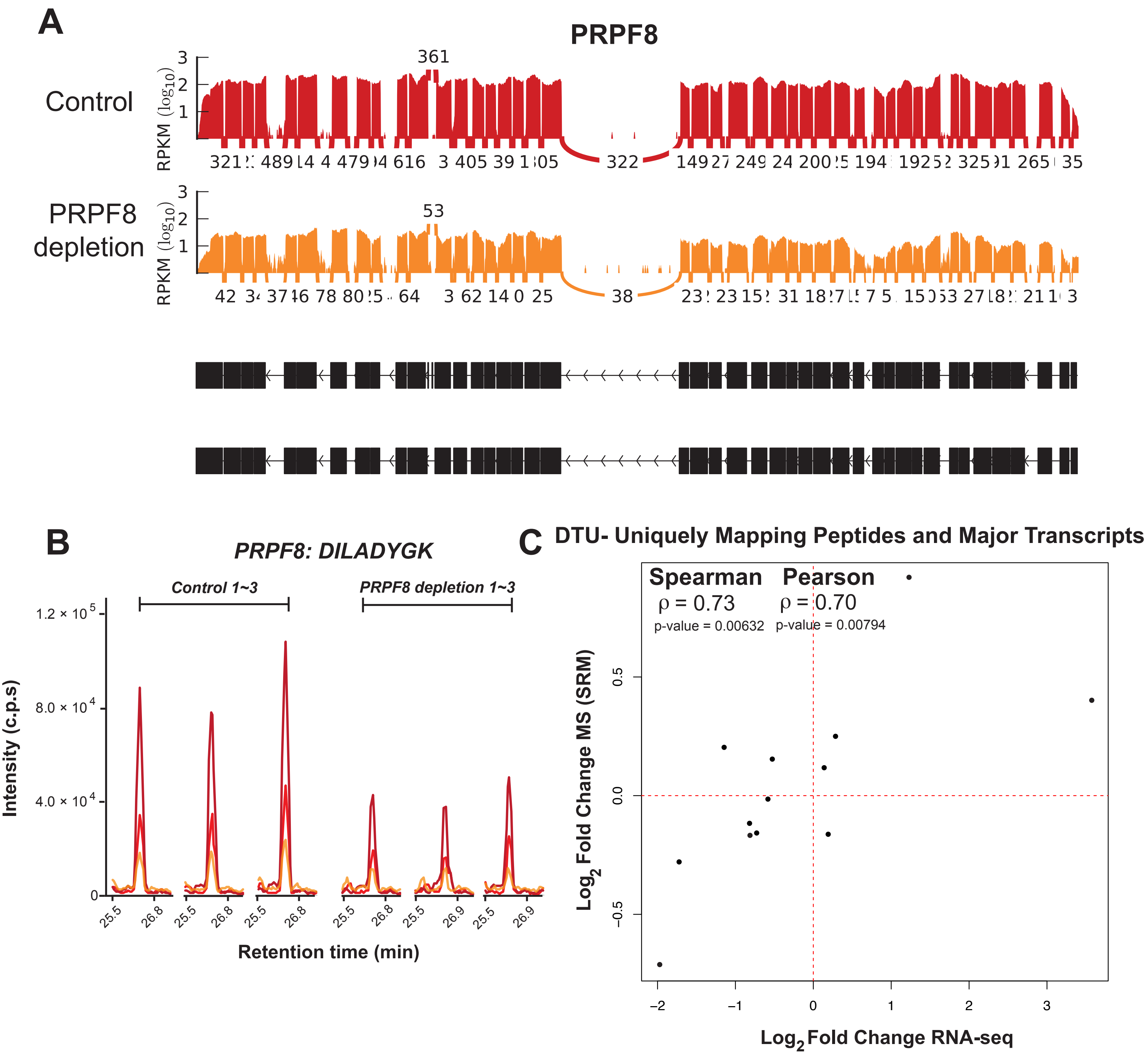

Figure S3, related to Figure 4: Validation using SRM mass spectrometry . **A**, Validation of PRPF8 depletion by RNA-sequencing. Coverage plot for the PRPF8 gene (control siRNA in red; PRPF8 siRNA in orange) obtained from RNA-sequencing data is shown. Note the reduction of reads across all exons after PRPF8 depletion. **B**, An example SRM plot for the PRPF8 peptide DILADYGK is shown for 3 biological replicates for Control and PRPF8 depleted samples. Intensity is represented on the Y-axis (c.p.s: counts per second). Efficiency of PRPF8 depletion was also verified by western blotting in Figure 5D. **C**, Correlation plot for major transcripts and uniquely mapping peptides using SRM. Scatterplot comparing changes in expression of differently used transcripts (DTU) whose most highly expressed isoform (major transcript) changes in expression (log2 fold change RNA-seq) to changes in expression of the peptides that uniquely map to them (log2 fold change SRM) after PRPF8 depletion. Spearman's correlation coefficient and p-value (correlation test) are shown in top left corner.

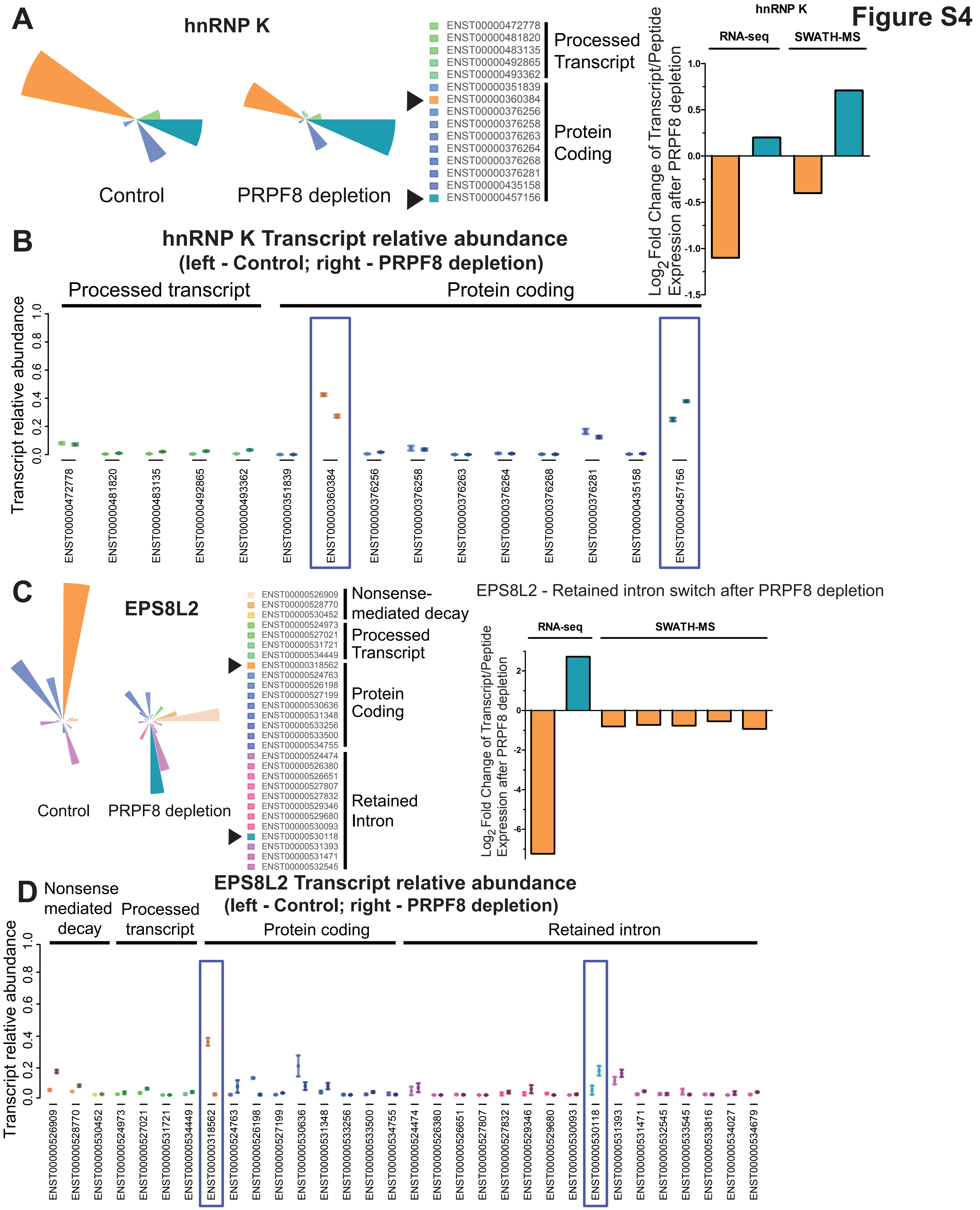

Figure S4, related to Figure 5 : Example of switch event that results in a change in protein isoform expression or a retained intron as determined by SWATH mass spectrometry. **A**, Starplot of transcript relative abundance for the hnRNPK gene is shown for control siRNA treated and PRPF8 depleted cells from one representative depletion experiment. The dominant transcript in Control cells is indicated in orange and in turquoise for PRPF8 depleted cells. Column plots show fold change in expression of these transcripts (left two columns) and their corresponding peptides (right two columns) after PRPF8 depletion as determined by SWATH-MS. **B**, For the hnRNPK gene, transcript relative abundance is also represented for each individual transcript (protein coding and processed transcripts). For each transcript, the transcript relative abundance in Control and PRPF8 depleted cells is on the left and right, respectively and represent the average from 3 independent depletion experiments. The major transcript in each condition is highlighted. **C,D** Starplot of transcript relative abundance for the EPS8L2 gene with corresponding fold change in peptide expression,

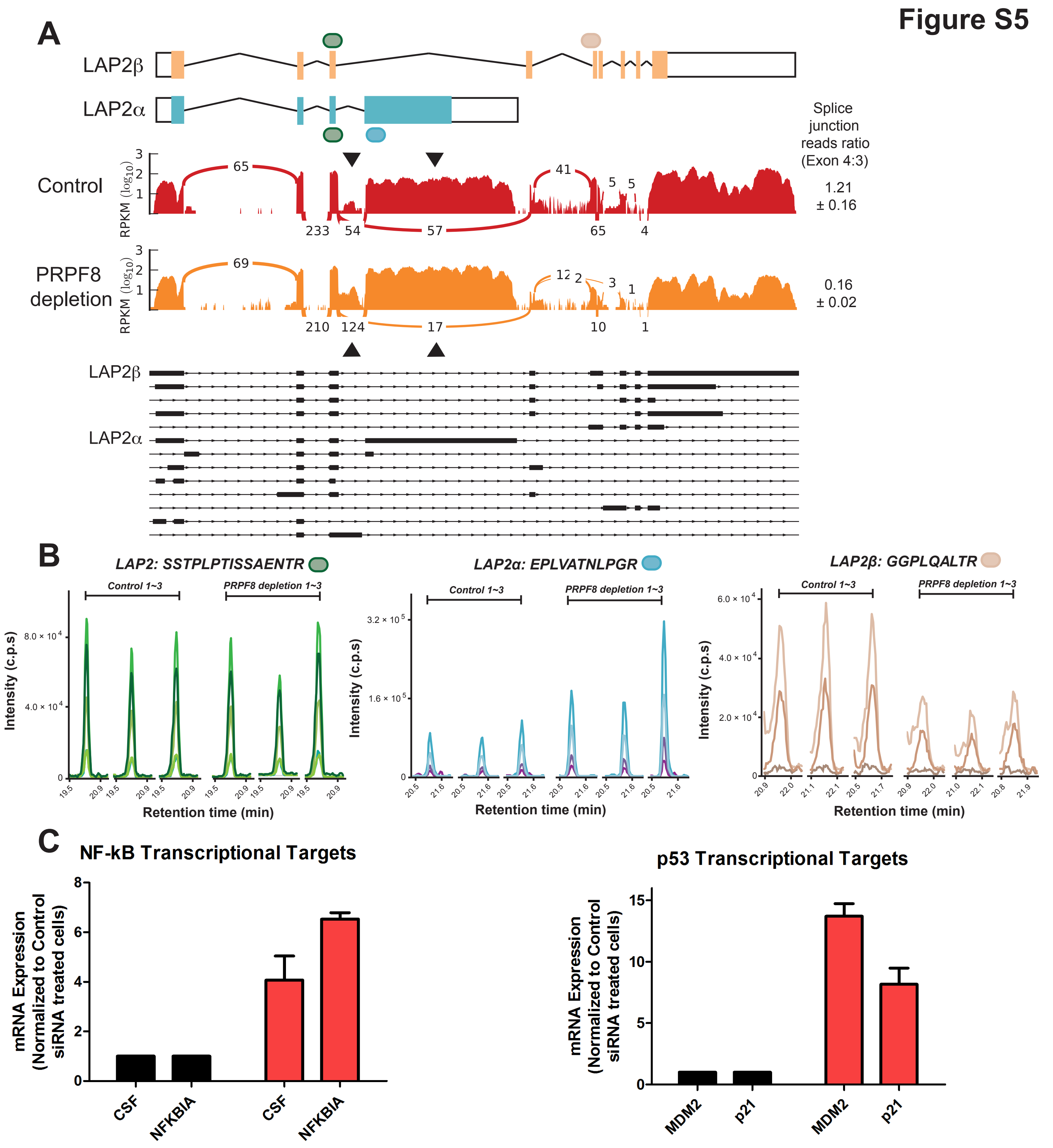

Figure S5, related to Figure 5: Validation of LAP2 switch event by SRM mass spectrometry. **A**, Coverage plot for LAP2 gene (control siRNA in red; PRPF8 siRNA in orange) obtained from RNA-sequencing data is shown. LAP2β and LAP2α isoform structures are indicated in orange and turquoise, respectively. The reduction of splice junction reads across exon 4 and 3 for the LAP2β isoform and corresponding increase for the LAP2α isoform after PRPF8 depletion is represented as a splice junction reads ratio. **B**, SRM plots for 3 peptides that map to LAP2β isoform (GGPLQALTR), LAP2α isoform (EPLVATNLPGR) and both isoforms (SSTPLPTISSAENTR) respectively are shown for 3 biological replicates for Control and PRPF8 depleted samples. Intensity is represented on the Y-axis (c.p.s: counts per second). Note that the peptide shared by both isoforms does not change in expression after PRPF8 depletion in contrast to those that map to the LAP2β and LAP2α isoforms. The quantification data was normalized by the intensity of the heavy peptide to remove run-to-run variation and the identity of the peptide was manually confirmed by the heavy isotopic peptide standard. **C**, Direct NF-kB and p53 transcriptional target genes are de-repressed after PRPF8 depletion. Consistent with a reduction in the levels of LAP2β, a known repressor of p53 and NF-kB target genes, we observe a de-repression of direct p53 and NF-kB transcriptional targets after PRPF8 depletion. For all qRT-PCR experiments in this figure, plots are relative to RNA levels in control siRNA-treated cells, assigned an arbitrary value of 1, and show the mean of triplicate readings from at least 3 independent depletion experiments, ± s.e.m. NF-kB and p53 transcriptional targets are represented in the left and right graphs, respectively.

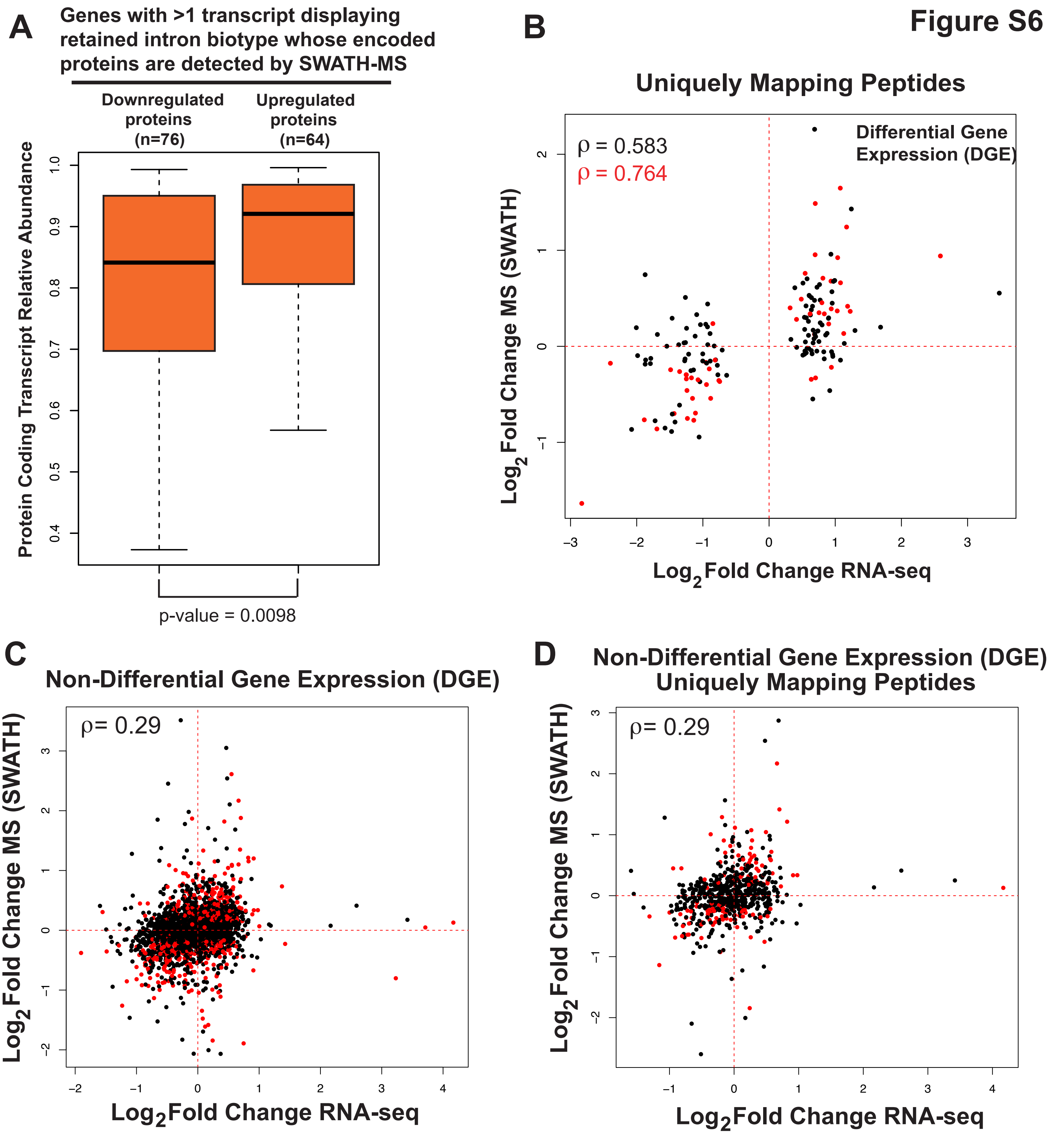

Figure S6, related to Figure 6: **A**, Relative abundance of protein-coding transcripts for each gene has a significant effect on regulating protein expression. Protein coding transcript relative abundance is shown for genes with >1 transcript displaying a retained intron biotype whose encoded proteins are detected by SWATH-MS. Boxplots representing downregulated and upregulated proteins after PRPF8 depletion are shown. Downregulated proteins have a higher relative abundance of transcripts that are not protein coding (i.e. display intron retention) in comparison to upregulated proteins and the corresponding p-value is indicated at the bottom of the boxplot (Wilcoxon test). **B**, Differential gene expression for uniquely mapping peptides. Scatterplot comparing changes in expression of differentially expressed genes (DGE) (log2 fold change RNA-seq) to changes in expression of the peptides that map uniquely to them (log2 fold change SWATH-MS) after PRPF8 depletion. Spearman's correlation coefficient is shown in top left corner. Differently expressed genes whose corresponding peptides change significantly in expression (adjusted p-value <0.1, t-test) are indicated in red and associated correlation coefficient is also shown in red. **C**, Non-differently expressed genes show a poor correlation when comparing RNA and protein fold-changes in expression after PRPF8 depletion. Scatterplot comparing changes in expression of non-differentially expressed genes (non-DGE) (log2 fold change RNA-seq) to changes in expression of the peptides that map to them (log2 fold change SWATH-MS) after PRPF8 depletion. Spearman's correlation coefficient is shown in top left corner. Differently expressed genes whose corresponding peptides change significantly in expression (adjusted p-value <0.1, t-test) are indicated in red. A similar plot for uniquely mapping peptides is shown in **D**.

DTU all transcripts + uniquely mapping peptides

transcript (tx) set DTU all #		peptide set uniquely mapping #			initial overlap	after assignment	Correlation coefficient (ρ)		agreement (%)		
				transcript	14	14	ρ	0.776	Y	11	78.57
tx	299	peptides	112	peptides	35	35	p-value	0.00174	N	3	21.43
genes	254	genes	51	genes	13	13					

DTU all transcripts + all peptides

transcript (tx) DTU all #		peptide set #			initial overlap	after assignment	Correlation coefficient (ρ)		agreement (%)		
				transcript	22	17	ρ	0.498	Y	12	70.59
tx	299	peptides	187	peptides	59	51	p-value	0.04418	N	5	29.41
genes	254	genes	70	genes	17	16					

DTU major transcripts + uniquely mapping peptides

transcript (tx) set DTU all #		peptide set uniquely mapping #			initial overlap	after assignment	Correlation coefficient (ρ)		agreement (%)		
				transcript	13	13	ρ	0.731	Y	10	76.92
tx	191	peptides	112	peptides	33	33	p-value	0.00632	N	3	23.08
genes	171	genes	51	genes	12	12					

DTU major transcripts + all peptides

transcript (tx) set DTU all #		peptide set #			initial overlap	after assignment	Correlation coefficient (ρ)		agreement (%)		
				transcript	19	16	ρ	0.624	Y	12	75.00
tx	191	peptides	187	peptides	56	53	p-value	0.01159	N	4	25.00
genes	171	genes	70	genes	16	15					

Table S1, related to Figure 4: Alternative integration strategies for differently used transcripts and peptides detected by SRM mass spectrometry

DTU all transcripts + uniquely mapping peptides			
**Correlation coefficient (SWATH)**		**Correlation coefficient (SRM)**	
ρ	0.301	ρ	0.723
**p-value**	0.110	**p-value**	0.005
**DTU all transcripts + all peptides**			
**Correlation coefficient (SWATH)**		**Correlation coefficient (SRM)**	
ρ	0.273	ρ	0.667
**p-value**	0.003	**p-value**	0.004
**DTU major transcripts + uniquely mapping peptides**			
**Correlation coefficient (SWATH)**		**Correlation coefficient (SRM)**	
ρ	0.258	ρ	0.665
**p-value**	0.211	**p-value**	0.016
**DTU major transcripts + all peptides**			
**Correlation coefficient (SWATH)**		**Correlation coefficient (SRM)**	
ρ	0.425	ρ	0.682
**p-value**	0.0002	**p-value**	0.0047

Table S2, related to Figures 2, 4 : Correlation coefficients for differently used transcripts and peptides detected by SWATH/SRM mass spectrometry using an alternative strategy to determine peptide fold-changes for each transcript

Differently Expressed Genes (DGE) using SWATH dataset												
**Uniquely mapping peptides**												
**transcript set DGE**		**peptide set uniquely mapping**	**#**			**initial overlap**	**after assignment**	**correlation**		**agreement (%)**		
	**#**	**peptides**	2974	**peptides**	594		594	**rho**	0.583	**Y**	141	76.63
**genes**	2021	**genes**	859	**genes**	184		184	**p-value**	0.0E+00	**N**	43	23.37
								**correlation** adjusted p-value <0.1		**agreement (%)**		
								**rho**	0.764	**Y**	54	93.10
								**p-value**	0	**N**	4	6.90
**All peptides**												
**transcript set DGE**		**peptide set**	**#**			**initial overlap**	**after assignment**	**correlation**		**agreement (%)**		
	**#**	**peptides**	14695	**peptides**	3057		3057	**rho**	0.626	**Y**	444	77.62
**genes**	2021	**genes**	2805	**genes**	572		572	**p-value**	0	**N**	128	22.38
								**correlation** adjusted p-value <0.1		**agreement (%)**		
								**rho**	0.794	**Y**	213	91.42
								**p-value**	0	**N**	20	8.58

Table S3, related to Figure 6: Alternative integration strategies for differently expressed genes and peptides detected by SWATH mass spectrometry

Supplemental Experimental Procedures

Cell Culture

Cal51 breast adenocarcinoma cells were a gift from Professor Paul Edwards, Department of Pathology, University of Cambridge. They were cultured in Dulbecco's Modified Eagle Medium (Invitrogen) with 10% fetal calf serum

(Invitrogen) and 1x penicillin-streptomycin (Invitrogen), and routinely tested for mycoplasma contamination.

Sample preparation

For siRNA-mediated depletion, Cal51 cells were reverse transfected with 25 nM siRNA to PRPF8 (Qiagen) using DharmaFECT1 (Dharmafect) transfection reagent, as previously described (Wickramasinghe et al., 2015). Transfected cells were harvested 54 hours later for RNA extraction and mass spectrometry from at least 3 independent depletion experiments.

Western blotting

Efficiency of depletion was monitored by western blotting with PRPF8 antibody (clone 2834C1a, ab51366, Abcam). For LAP2 isoform detection, antibodies were used that specifically recognised the α isoform (ab5162, Abcam), the β isoform (06-1002, Millipore), and both isoforms (Clone 6E10, Sigma).

RNA extraction, RT-PCR and qRT-PCR

RNA was isolated from siRNA-treated cells with an RNeasy kit (Qiagen) according to manufacturer's instructions. Isolated RNA was quantified with a NanoDrop 1000 (Thermo Scientific) and quality was determined by measuring the A_{260}/A_{280} ratio, which was always between 1.8 and 2.1, and stored at -80ºC. One μg of RNA was used for cDNA synthesis using the QuantiTect Reverse Transcription Kit (Qiagen) according to manufacturer's instructions. Synthesized cDNA was diluted following reverse transcriptase inactivation and stored at –20ºC. Primers for qPCR were designed to bridge exon-intron junctions. For RT-PCR experiments, PCR was conducted on a MJ Research thermal cycler using Accuprime Pfx DNA polymerase (Invitrogen), forward

and reverse primer and cDNA. qPCR was conducted on a Rotorgene RG-3000 (Corbett Research) machine using 2x SYBR-Green Master Mix (Roche), forward and reverse primer and cDNA. The cycling acquisition program was as follows: 50ºC 2 minutes, 95ºC 2 minutes, 50 cycles of 95ºC for 15 seconds and 60ºC for 30 seconds. The C_t values were calculated, referenced to standard curves for each primer set. All samples were then normalized to control siRNA treated samples.

Immunofluorescence

Immunofluorescence was performed as previously described (Wickramasinghe et al., 2010). Briefly, cells were fixed in 4% paraformaldehyde for 5 min at room temperature and permeabilised in PBS, 0.1% Triton X-100 (Sigma) and 0.02% SDS for 10 min at room temperature. After 30 minutes in blocking buffer (permeabilisation buffer + 1 % BSA), coverslips were incubated with the appropriate primary (α isoform - ab5162, Abcam; β isoform - 06-1002, Millipore; both isoforms - Clone 6E10, Sigma) and secondary antibodies (Molecular Probes) and examined using a Zeiss LSM510 Meta confocal microscope. Scanning analysis of cells was performed using ImageJ software (NIH). All images used for comparative analysis were acquired using identical microscope settings. A line width of 20 was used, and pairs of cells with nuclei of same scan width as indicated by DAPI staining were used for analysis. All analyses are representative of the cell population.

Analysis of RNA-sequencing data

The transcriptome of control siRNA-treated and PRPF8 depleted Cal51 cells was sequenced on an Illumina HiSeq2000 platform using 100 bp paired-end reads with poly(A)+RNA isolated from 3 and 4 independent experiments,

respectively, as previously described (Wickramasinghe et al., 2015). Raw reads were directly mapped to the transcriptome with Bowtie v0.12.7 (Langmead et al., 2009), using Ensembl v66 as a reference (Flicek et al., 2012). Following the estimation of transcript expression levels with MMSEQ v1.0.7 (Turro et al., 2011), its companion tool MMDIFF (Turro et al., 2014) was used to identify both differentially expressed genes and differentially used transcripts. MMDIFF uses Bayesian inference to evaluate the probability that two genes are differentially expressed / two transcripts are differentially used across conditions, which is termed 'posterior probability'. A posterior probability of 0.85 was used as the significance threshold for analysing the SWATH data and 0.9 for the SRM data. Switch events within the set of genes identified to undergo differential transcript usage were identified with SwitchSeq (Gonzalez-Porta and Brazma, 2015). Switch events that involved major transcripts with identical protein sequences were removed from the analyses.

Protein extraction and in-solution digestion.

The cell pellets from three independent depletion experiments (control siRNA and PRPF8 depleted) were lysed on ice by using a lysis buffer containing 8 M urea (EuroBio), 40 mM Tris-base (Sigma-Aldrich), 10 mM DTT (AppliChem) and complete protease inhibitor cocktail (Roche). The resulted mixture was sonicated at 4 °C for 5 mins using a VialTweeter device (Hielscher-Ultrasound Technology) and centrifuged at 21130 g, 4 °C for 1 hr to remove the insoluble material. The supernatant protein mixtures were transferred and protein amount was determined using a Bradford assay (Bio-Rad, Hercules, CA, USA). Aliquots of 1 mg protein mixtures were reduced by 5 mM tris(carboxyethyl)phosphine (Sigma-Aldrich) and alkylated by 30 mM

iodoacetamide (Sigma-Aldrich). Then 5 volumes of precooled precipitation solution containing 50% acetone, 50% ethanol, and 0.1% acetic acid was added to the protein mixture and kept at −20 °C overnight. The mixture was centrifuged at 20,400 *g* for 40 min. The pellets were washed with 100% acetone and 70% ethanol with centrifugation at 20,400 *g* for 40 min. The samples were then resolved by 100 mM NH_4HCO_3 and were digested with sequencing-grade porcine trypsin (Promega) at a protease/protein ratio of 1:50 overnight at 37 °C (Kim et al., 2006). Digests were purified with Vydac C18 Silica MicroSpin columns (The Nest Group Inc.). Peptide amount was determined using Nanodrop ND-1000 (Thermo Scientific) and about 0.7 μg peptide mixtures were analyzed in each LC-MS run. An aliquot of retention time calibration peptides from iRT-Kit (Biognosys) was spiked into each sample before all LC-MS analysis at a ratio of 1:30 (v/v) to correct relative retention times between runs (Escher et al., 2012).

Shotgun measurement.

The peptides digested from Cal51 lysate were all measured on an AB Sciex 5600 TripleTOF mass spectrometer operated in DDA mode. The mass spectrometer was interfaced with an Eksigent NanoLC Ultra 2D Plus HPLC system as previously described(Collins et al., 2013; Gillet et al., 2012; Liu et al., 2013). Peptides were directly injected onto a 20-cm PicoFrit emitter (New Objective, self-packed to 20 cm with Magic C18 AQ 3-μm 200-Å material), and then separated using a 120-min gradient from 2–35% (buffer A 0.1% (v/v) formic acid, 2% (v/v) acetonitrile, buffer B 0.1% (v/v) formic acid, 90% (v/v) acetonitrile) at a flow rate of 300 nL/min. MS1 spectra were collected in the range 360–1,460 m/z. The 20 most intense precursors with charge state 2–5 which exceeded 250 counts per second were selected for fragmentation, and

MS2 spectra were collected in the range 50–2,000 m/z for 100 ms. The precursor ions were dynamically excluded from reselection for 20 s.

Peptide identification and transcript mapping.

Profile-mode .wiff files from shotgun data of Cal51 cells, together with those of HEK293, LNCap, U2OS and HeLa cells included in the previously published SWATHatlas (34 runs in total, for the purpose of increasing the coverage of the transcript-centric spectral library used in this study)(Rosenberger et al., 2014) were all centroided and converted to mzML format using the Sciex Data Converter v.1.3 and converted to mzXML format using MSConvert v.3.04.238. The MS2 spectra were queried against the fasta file of Ensembl 66 appended with reversed sequence decoys (Elias and Gygi, 2007). Two types of search engines, xTandem (Falkner and Andrews, 2005) and Omssa (Geer et al., 2004), were used through iPortal interface for sophisticated proteomic workflows (Kunszt et al., 2015). The search parameters are: static modifications of 57.02146 Da for cysteines, variable modifications of 15.99491 Da for methionine oxidations. The parent mass tolerance was set to be 30 p.p.m and mono-isotopic fragment mass tolerance was 50 p.p.m. Fully-tryptic peptides and peptides with up to two missed cleavages were allowed. The identified peptides were processed and analyzed through Trans-Proteomic Pipeline 4.5.2 (TPP) (Keller et al., 2005) and were validated using the *PeptideProphet* score (Keller et al., 2002) . All the peptides were filtered at a false discovery rate (FDR) of 1%.

SWATH-MS measurement.

The same LC-MS/MS systems used for DDA measurements was also used for SWATH analysis (Collins et al., 2013; Gillet et al., 2012; Liu et al., 2013).

Specifically, in the present SWATH-MS mode, the SCIEX 5600 plus TripleTOF instrument was specifically tuned to optimize the quadrupole settings for the selection of 64 variable wide precursor ion selection windows. The 64-variable window schema was optimized based on a normal human cell lysate sample, covering the precursor mass range of 400–1,200 m/z. The effective isolation windows can be considered as being 399.5~408.2, 407.2~415.8, 414.8~422.7, 421.7~429.7, 428.7~437.3, 436.3~444.8, 443.8~451.7, 450.7~458.7, 457.7~466.7, 465.7~473.4, 472.4~478.3, 477.3~485.4, 484.4~491.2, 490.2~497.7, 496.7~504.3, 503.3~511.2, 510.2~518.2, 517.2~525.3, 524.3~533.3, 532.3~540.3, 539.3~546.8, 545.8~554.5, 553.5~561.8, 560.8~568.3, 567.3~575.7, 574.7~582.3, 581.3~588.8, 587.8~595.8, 594.8~601.8, 600.8~608.9, 607.9~616.9, 615.9~624.8, 623.8~632.2, 631.2~640.8, 639.8~647.9, 646.9~654.8, 653.8~661.5, 660.5~670.3, 669.3~678.8, 677.8~687.8, 686.8~696.9, 695.9~706.9, 705.9~715.9, 714.9~726.2, 725.2~737.4, 736.4~746.6, 745.6~757.5, 756.5~767.9, 766.9~779.5, 778.5~792.9, 791.9~807, 806~820, 819~834.2, 833.2~849.4, 848.4~866, 865~884.4, 883.4~899.9, 898.9~919, 918~942.1, 941.1~971.6, 970.6~1006, 1005~1053, 1052~1110.6, 1109.6~1200.5 (containing 1 m/z for the window overlap). SWATH MS2 spectra were collected from 50 to 2,000 m/z. The collision energy (CE) was optimized for each window according to the calculation for a charge 2+ ion centered upon the window with a spread of 15 eV. An accumulation time (dwell time) of 50 ms was used for all fragment-ion scans in high-sensitivity mode and for each SWATH-MS cycle a survey scan in high-resolution mode was also acquired for 250 ms, resulting in a duty cycle of ~3.45 s.

Spectral library generation and targeted data analysis.

The raw spectral libraries were generated from all valid peptide spectrum matches for the shotgun measurement of the light peptides, and then refined into the non redundant consensus libraries(Collins et al., 2013) using SpectraST (Lam et al., 2007). For each peptide, the retention time was mapped into the iRT space (Escher et al., 2012) with reference to a linear calibration constructed for each shotgun run, as previously described (Collins et al., 2013). The MS assays constructed from Top 5 most intense transitions with Q1 range from 400 to 1200 m/z excluding the precursor SWATH window were used for targeted data analysis of SWATH maps. The whole process of SWATH targeted data analysis was carried out using OpenSWATH (Rost et al., 2014). Based the spectral library generated above, OpenSWATH firstly identified the peak groups from all individual SWATH maps at a global peptide FDR=1% and then aligned them between SWATH maps (a total of 12 files including technical and biological replicates) based on the clustering behaviors of retention time in each run with a non-linear alignment algorithm (Weisser et al., 2013). Specifically, only those peptide peak groups identified in more than 75% samples (i.e., 9 files) were reported and considered for alignment with the max extension FDR of 0.05 (quality cutoff to still consider a feature for alignment) and/or the further constraint of less than 60 second RT difference in LC gradient after iRT normalization (Liu et al., 2015). The imputed data generated from the requantification option in OpenSWATH was not used.

Peptide selection and SRM measurement.

The peptide selection of SRM was directed mainly by shotgun identification results and also the prediction of MS peptide detectability using CONSeQuence software (Eyers et al., 2011) for those targeted transcripts without shotgun identification. Isotopically-labeled heavy forms (containing

either a C-terminal [$^{13}C6^{15}N4$] Arg or [$^{13}C6^{15}N2$] Lys residue) of selected peptides were synthesized by JPT Peptide Technologies. After synthesis, all peptides were resuspended in 20 % acetonitrile, 1 % formic acid and sonicated for 15 minutes. These heavy isotope-labeled peptides were then diluted into 2 % acetonitrile containing 0.1 % formic acid during the preparation of injections. Peptide samples were analyzed on a hybrid triple quadrupole/ion trap mass spectrometer (5500QTRAP, AB Sciex) equipped with a nanoelectrospray ion source. Chromatographic separation of peptides was performed by a nanoLC ultra 1Dplus system (Eksigent) coupled to a 15 cm fused silica emitter. Peptides were separated in a 35 minutes gradient of 5 – 35% acetonitrile in 0.1 % formic acid (v/v) at a flow rate of 300 nL/min (Huttenhain et al., 2012; Liu et al., 2013). Both Q1 and Q3 operated at unit resolution and a cycle time of 3s at scheduled mode (8 min window). To keep enough dwell time, the whole method was split into around 410 transitions per run. CEs were calculated according to previous studies (Lange et al., 2008; Liu et al., 2013). SRM data was manually inspected and analyzed using Skyline (MacLean et al., 2010) and normalized based on the heavy peptide standards. Finally 187 peptides were confidently quantified by SRM with reliable light/heavy pairs, of which 51 peptides mapped to 17 differentially used transcripts.

Assignment of peptides to transcripts

An initial set of 16,779 peptides was detected across biological replicates for each condition (control siRNA and PRPF8-depleted samples) using SWATH mass spectrometry and mapped against all the protein coding transcripts annotated in Ensembl v66, including those with a nonsense-mediated decay biotype. Removal of peptides that mapped to more than one gene led to a set of 14,695 peptides (corresponding to 2,805 genes), which was used for

downstream analysis. Peptides were assigned to specific transcripts as outlined in Figure 1. Peptides that map uniquely to each transcript represented a minority of events (2974 peptides mapping to 859 genes). Peptides that map ubiquitously to several transcripts of the same gene were assigned based on knowledge from the RNA-sequencing experiments using the following criteria. Two alternative peptide assignment strategies were considered. One strategy incorporated information on transcript isoform abundance for each gene into our analysis, whereby only peptides that map to major transcripts were considered. Major transcripts are the dominant expressed isoform for each gene and those identified as major in either control siRNA-treated or PRPF8 depleted samples were used specifically for peptide assignment. Additionally, we considered an alternative assignment strategy where information about transcript expression levels was not considered. Specifically, if a peptide maps to multiple transcripts in the same gene, but the expression of only one of these transcripts was changed after PRPF8 depletion, then this peptide was assigned to that particular transcript regardless of its expression level. In contrast, peptides that map simultaneously to multiple differentially used transcripts were considered ambiguous and were not used for further analysis.

Integration of transcriptomic and proteomic data

To integrate transcriptomic and proteomic data, fold-changes in transcript and peptide expression after PRPF8 depletion were obtained from RNA-sequencing and SWATH or SRM mass spectrometry experiments, respectively. RNA-sequencing fold-changes were calculated from the transcript-level expression estimates obtained from MMSEQ as described above. For each transcript, the fold-change represents the median transcript

expression in PRPF8 depleted vs. control siRNA treated samples.

Raw peptide intensities were first quantile-normalised in order to enable comparison across samples. For each peptide, the observed intensities across the biological replicates in each condition were summarised by using the median, and a fold-change was obtained by dividing the value obtained for PRPF8 depleted and control siRNA-treated samples. Peptide fold-changes for each transcript were calculated by first adding up the intensities of all the peptides that mapped to that transcript in each given biological replicate, and then dividing the median value of the summed peptide signals for PRPF8 depletion vs. controls (hence resulting in one fold-change per transcript). The same analysis was used for both SWATH and SRM datasets. Use of an alternative strategy to determine peptide fold-changes for each transcript, whereby the fold change for PRPF8 depletion vs. controls was determined individually for each peptide to obtain the median fold-change of all peptides that mapped to that transcript, yielded similar results (see Table 3). The fold-changes derived from these two technologies were integrated as described in Figure 1. Spearman correlation was used to evaluate the relationship between transcript and peptide fold-changes, as previously suggested (Maier et al., 2009). We also used Pearson correlation as a comparison.

For the retained intron analysis from Figure 6A, a list of genes previously identified to undergo intron retention events following PRPF8 depletion was used (n=2,086) (Wickramasinghe et al., 2015). Peptides were mapped to specific genes following the approach depicted in Figure 1, except a gene-centric approach was used, in contrast to the transcript-centric approach used for DTU analysis. Peptide fold-changes for each gene were then calculated by first adding up the intensities of all the peptides that mapped to that gene in each given replicate (using all available peptide data), and then dividing the median value of the summed peptide signals for PRPF8 depletion vs. controls

(hence resulting in one fold-change per gene/protein). Significance was evaluated using a t-test (adjusted p-value < 0.1). Peptides with significant fold changes in expression were used for analysis, resulting in a data set with 743 genes (out of 2805) for SWATH, of which 270 displayed retained introns, and 473 genes that do not display intron retention.

For differential gene expression analysis in Figure 6B, differentially expressed genes were obtained with MMDIFF, using a significance threshold of 0.85 for the posterior probability. Gene expression fold-changes were then calculated from MMSEQ output using the same strategy as that used for transcripts. Protein fold-changes were calculated as above from SWATH experiments and fold-change significance was assessed with a t-test, and a p-value of 0.1 was used as the significance threshold. Spearman correlation was also used to evaluate the relationship between gene and protein fold-changes.

For Figure S1, gene ontology analysis was performed using DAVID (Huang da et al., 2009). Proteins with altered expression levels were designated as such if at least one peptide per protein displayed a fold-change of greater than 1.25 fold or less than 0.75 fold after PRPF8 depletion. In the case of protein analysis, the set of proteins detected by SWATH-MS was used as a background (n = 2805).

Data availability

All the raw data of mass spectrometry measurements (SWATH-MS and shotgun), together with the input spectral library and OpenSWATH results can be freely downloaded from ProteomeXchange Consortium (http://proteomecentral.proteomexchange.org) via identifier PXD003278. The

RNA-sequencing data can be accessed from the ArrayExpress database with the accession number E-MTAB-3021.

Supplemental References

Collins, B.C., Gillet, L.C., Rosenberger, G., Rost, H.L., Vichalkovski, A., Gstaiger, M., and Aebersold, R. (2013). Quantifying protein interaction dynamics by SWATH mass spectrometry: application to the 14-3-3 system. Nature methods.

Elias, J.E., and Gygi, S.P. (2007). Target-decoy search strategy for increased confidence in large-scale protein identifications by mass spectrometry. Nature methods *4*, 207-214.

Escher, C., Reiter, L., MacLean, B., Ossola, R., Herzog, F., Chilton, J., MacCoss, M.J., and Rinner, O. (2012). Using iRT, a normalized retention time for more targeted measurement of peptides. Proteomics *12*, 1111-1121.

Eyers, C.E., Lawless, C., Wedge, D.C., Lau, K.W., Gaskell, S.J., and Hubbard, S.J. (2011). CONSeQuence: prediction of reference peptides for absolute quantitative proteomics using consensus machine learning approaches. Molecular & cellular proteomics : MCP *10*, M110 003384.

Falkner, J., and Andrews, P. (2005). Fast tandem mass spectra-based protein identification regardless of the number of spectra or potential modifications examined. Bioinformatics *21*, 2177-2184.

Flicek, P., Amode, M.R., Barrell, D., Beal, K., Brent, S., Carvalho-Silva, D., Clapham, P., Coates, G., Fairley, S., Fitzgerald, S.*, et al.* (2012). Ensembl 2012. Nucleic Acids Res *40*, D84-90.

Geer, L.Y., Markey, S.P., Kowalak, J.A., Wagner, L., Xu, M., Maynard, D.M., Yang, X., Shi, W., and Bryant, S.H. (2004). Open mass spectrometry search algorithm. Journal of proteome research *3*, 958-964.

Gillet, L.C., Navarro, P., Tate, S., Rost, H., Selevsek, N., Reiter, L., Bonner, R., and Aebersold, R. (2012). Targeted data extraction of the MS/MS spectra generated by data-independent acquisition: a new concept for consistent and accurate proteome analysis. Molecular & cellular proteomics : MCP *11*, O111 016717.

Gonzalez-Porta, M., and Brazma, A. (2015). Identification, annotation and visualisation of extreme changes in splicing from RNA-seq experiments with SwitchSeq. http://biorxiv.org/content/biorxiv/early/2014/2006/2006/005967.full.pdf.

Huang da, W., Sherman, B.T., and Lempicki, R.A. (2009). Systematic and integrative analysis of large gene lists using DAVID bioinformatics resources. Nat Protoc *4*, 44-57.

Huttenhain, R., Soste, M., Selevsek, N., Rost, H., Sethi, A., Carapito, C., Farrah, T., Deutsch, E.W., Kusebauch, U., Moritz, R.L.*, et al.* (2012). Reproducible quantification of cancer-associated proteins in body fluids using targeted proteomics. Science translational medicine *4*, 142ra194.

Keller, A., Eng, J., Zhang, N., Li, X.J., and Aebersold, R. (2005). A uniform proteomics MS/MS analysis platform utilizing open XML file formats. Mol Syst Biol *1*, 2005 0017.

Keller, A., Nesvizhskii, A.I., Kolker, E., and Aebersold, R. (2002). Empirical statistical model to estimate the accuracy of peptide identifications made by MS/MS and database search. Anal Chem *74*, 5383-5392.
Kim, S.C., Chen, Y., Mirza, S., Xu, Y., Lee, J., Liu, P., and Zhao, Y. (2006). A clean, more efficient method for in-solution digestion of protein mixtures without detergent or urea. Journal of proteome research *5*, 3446-3452.
Kunszt, P., Blum, L., Hullár, B., Schmid, E., Srebniak, A., Wolski, W., Rinn, B., Elmer, F., Ramakrishnan, C., Quandt, A.*, et al.* (2015). iPortal: the swiss grid proteomics portal: Requirements and new features based on experience and usability considerations. Concurrency and computation : practice & experience *27*, 433-445.
Lam, H., Deutsch, E.W., Eddes, J.S., Eng, J.K., King, N., Stein, S.E., and Aebersold, R. (2007). Development and validation of a spectral library searching method for peptide identification from MS/MS. Proteomics *7*, 655-667.
Lange, V., Picotti, P., Domon, B., and Aebersold, R. (2008). Selected reaction monitoring for quantitative proteomics: a tutorial. Molecular systems biology *4*, 222.
Langmead, B., Trapnell, C., Pop, M., and Salzberg, S.L. (2009). Ultrafast and memory-efficient alignment of short DNA sequences to the human genome. Genome biology *10*, R25.
Liu, Y., Buil, A., Collins, B.C., Gillet, L.C., Blum, L.C., Cheng, L.Y., Vitek, O., Mouritsen, J., Lachance, G., Spector, T.D.*, et al.* (2015). Quantitative variability of 342 plasma proteins in a human twin population. Mol Syst Biol *11*, 786.
Liu, Y., Huttenhain, R., Surinova, S., Gillet, L.C., Mouritsen, J., Brunner, R., Navarro, P., and Aebersold, R. (2013). Quantitative measurements of N-linked glycoproteins in human plasma by SWATH-MS. Proteomics *13*, 1247-1256.
MacLean, B., Tomazela, D.M., Shulman, N., Chambers, M., Finney, G.L., Frewen, B., Kern, R., Tabb, D.L., Liebler, D.C., and MacCoss, M.J. (2010). Skyline: an open source document editor for creating and analyzing targeted proteomics experiments. Bioinformatics *26*, 966-968.
Maier, T., Guell, M., and Serrano, L. (2009). Correlation of mRNA and protein in complex biological samples. FEBS Lett *583*, 3966-3973.
Rosenberger, G., Koh, C.C., Guo, T., Rost, H.L., Kouvonen, P., Collins, B.C., Heusel, M., Liu, Y., Caron, E., Vichalkovski, A.*, et al.* (2014). A repository of assays to quantify 10,000 human proteins by SWATH-MS. Scientific data *1*, 140031.
Rost, H.L., Rosenberger, G., Navarro, P., Gillet, L., Miladinovic, S.M., Schubert, O.T., Wolski, W., Collins, B.C., Malmstrom, J., Malmstrom, L.*, et al.* (2014). OpenSWATH enables automated, targeted analysis of data-independent acquisition MS data. Nature biotechnology *32*, 219-223.
Turro, E., Astle, W.J., and Tavare, S. (2014). Flexible analysis of RNA-seq data using mixed effects models. Bioinformatics *30*, 180-188.
Turro, E., Su, S.Y., Goncalves, A., Coin, L.J., Richardson, S., and Lewin, A. (2011). Haplotype and isoform specific expression estimation using multi-mapping RNA-seq reads. Genome Biol *12*, R13.
Weisser, H., Nahnsen, S., Grossmann, J., Nilse, L., Quandt, A., Brauer, H., Sturm, M., Kenar, E., Kohlbacher, O., Aebersold, R.*, et al.* (2013). An

Automated Pipeline for High-Throughput Label-Free Quantitative Proteomics. Journal of proteome research.
Wickramasinghe, V.O., Gonzalez-Porta, M., Perera, D., Bartolozzi, A.R., Sibley, C.R., Hallegger, M., Ule, J., Marioni, J.C., and Venkitaraman, A.R. (2015). Regulation of constitutive and alternative mRNA splicing across the human transcriptome by PRPF8 is determined by 5' splice site strength. Genome Biol *16*, 201.
Wickramasinghe, V.O., McMurtrie, P.I., Mills, A.D., Takei, Y., Penrhyn-Lowe, S., Amagase, Y., Main, S., Marr, J., Stewart, M., and Laskey, R.A. (2010). mRNA export from mammalian cell nuclei is dependent on GANP. Curr Biol *20*, 25-31.
