# Supplementary material for: Impact of Alternative Splicing on the Human Proteome
Source: Cell Rep. 2017 Aug 1;20(5):1229–41. doi: 10.1016/j.celrep.2017.07.025 (PMC5554779; doi:10.1016/j.celrep.2017.07.025)
Supplement: Document S2. Article plus Supplemental Information [file mmc2.pdf]

Cell Reports

Resource

Impact of Alternative Splicing on the Human Proteome

Graphical Abstract

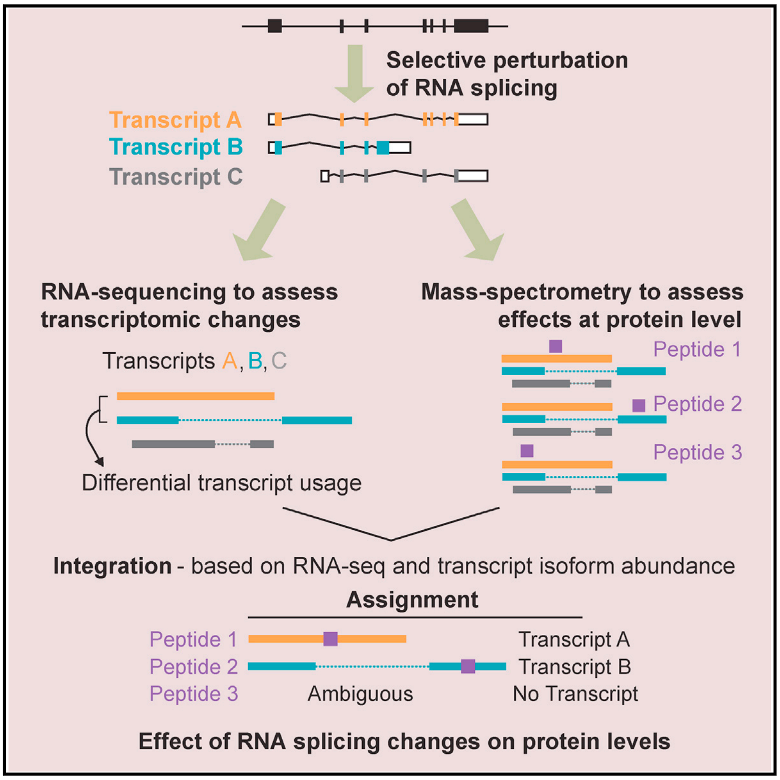

Authors

Yansheng Liu, Mar Gonzàlez-Porta, Sergio Santos, ..., Ruedi Aebersold, Ashok R. Venkitaraman, Vihandha O. Wickramasinghe

Correspondence

aebersold@imsb.biol.ethz.ch (R.A.), arv22@mrc-cu.cam.ac.uk (A.R.V.), vi.wickramasinghe@petermac.org (V.O.W.)

In Brief

Liu et al. have developed an integrative approach to ask whether perturbations in mRNA splicing patterns alter the composition of the proteome. Their findings illustrate how RNA splicing links isoform expression in the human transcriptome with proteomic diversity and provides a foundation for studying perturbations associated with human diseases.

Accession Numbers

PXD003278
E-MTAB-3021

Highlights

- Integrative approach to study contribution of alternative splicing to proteome
- Changes in isoform usage alter protein abundance proportionate to transcript levels
- Intron retention is accompanied by decreased protein abundance
- Differential gene expression functionally tunes the human proteome

Liu et al., 2017, Cell Reports *20*, 1229–1241
August 1, 2017 Crown Copyright © 2017
http://dx.doi.org/10.1016/j.celrep.2017.07.025

Cell Reports
Resource

OPEN ACCESS
CellPress

Impact of Alternative Splicing on the Human Proteome

Yansheng Liu,[1,5] Mar Gonzàlez-Porta,[2,5] Sergio Santos,[2] Alvis Brazma,[2] John C. Marioni,[2] Ruedi Aebersold,[1,*] Ashok R. Venkitaraman,[3,*] and Vihandha O. Wickramasinghe[3,4,6,*]
[1]Department of Biology, Institute of Molecular Systems Biology, ETH Zurich, Zurich, Switzerland
[2]European Molecular Biology Laboratory-European Bioinformatics Institute (EMBL-EBI), Hinxton, UK
[3]The Medical Research Council Cancer Unit, University of Cambridge, Cambridge CB2 0XZ, UK
[4]RNA Biology and Cancer Laboratory, Peter MacCallum Cancer Centre, Melbourne, VIC 3000, Australia
[5]These authors contributed equally
[6]Lead Contact
*Correspondence: aebersold@imsb.biol.ethz.ch (R.A.), arv22@mrc-cu.cam.ac.uk (A.R.V.), vi.wickramasinghe@petermac.org (V.O.W.)
http://dx.doi.org/10.1016/j.celrep.2017.07.025

SUMMARY

Alternative splicing is a critical determinant of genome complexity and, by implication, is assumed to engender proteomic diversity. This notion has not been experimentally tested in a targeted, quantitative manner. Here, we have developed an integrative approach to ask whether perturbations in mRNA splicing patterns alter the composition of the proteome. We integrate RNA sequencing (RNA-seq) (to comprehensively report intron retention, differential transcript usage, and gene expression) with a data-independent acquisition (DIA) method, SWATH-MS (sequential window acquisition of all theoretical spectra-mass spectrometry), to capture an unbiased, quantitative snapshot of the impact of constitutive and alternative splicing events on the proteome. Whereas intron retention is accompanied by decreased protein abundance, alterations in differential transcript usage and gene expression alter protein abundance proportionate to transcript levels. Our findings illustrate how RNA splicing links isoform expression in the human transcriptome with proteomic diversity and provides a foundation for studying perturbations associated with human diseases.

INTRODUCTION

Next-generation RNA sequencing (RNA-seq) has identified alternative splicing of RNA transcripts as a key mechanism that may underlie the diversification of proteins encoded in the human genome. Such diversification may be essential for biologic complexity, because the number of protein-coding human genes is lower than was widely predicted before the genome sequence was known (Kim et al., 2014; Lander et al., 2001). Transcripts from ~95% of multi-exon human genes are alternatively spliced (Pan et al., 2008; Wang et al., 2008). However, the extent to which this increased genomic complexity contributes to the generation of proteomic diversity is largely unknown. Initial efforts to assess the contribution of alternative splicing to proteomic composition and diversity have focused exclusively on the identification of proteins derived from alternatively spliced transcripts in a steady-state system (Blakeley et al., 2010; Brosch et al., 2011; Ezkurdia et al., 2012; Lander et al., 2001; Leoni et al., 2011; Tress et al., 2008; Xing et al., 2011; Zhou et al., 2010). More recent studies have incorporated expression data, such as evidence from RNA-seq experiments, in the interrogation of proteomic datasets to reduce mapping noise (Lopez-Casado et al., 2012; Ning and Nesvizhskii, 2010; Sheynkman et al., 2013; Tanner et al., 2007). However, none of these studies have attempted to quantify the contribution of alternative splicing to proteomic diversity in a systematic manner. Here, we seek to address this fundamental biological question by asking whether selective perturbations in RNA splicing patterns manifest as changes in the composition of the proteome. By using this system, we have established in a quantitative manner how changes in splicing of a subset of transcripts determine differential protein expression.

RESULTS AND DISCUSSION

Experimental Strategy to Study Alternative Splicing at the Proteomic Level

We selectively perturbed RNA splicing by depleting the core spliceosome U5 small nuclear ribonucleo protein (snRNP) component PRPF8 and assessed subsequent transcriptomic and proteomic changes by RNA-seq and SWATH-MS (sequential window acquisition of all theoretical spectra-mass spectrometry), respectively (Figure 1). This is a compelling system because a number of studies have demonstrated the regulatory potential of the core spliceosome machinery (Clark et al., 2002; Papasaikas et al., 2015; Park et al., 2004; Pleiss et al., 2007; Saltzman et al., 2011; Wickramasinghe et al., 2015). Furthermore, we have extensively experimentally validated this system for studying splicing at the mRNA level (Wickramasinghe et al., 2015). Thus, using DEXSeq (Anders et al., 2012), we previously identified 3,370 transcripts with altered splicing patterns after PRPF8 depletion (1,284 with differential exon usage, 1,449 with intron retention, 637 with both), which constitute only a subset of all expressed protein-coding genes (13,216 genes; expression threshold = 1 fragments per kilobase million

This is an open access article under the CC BY license (http://creativecommons.org/licenses/by/4.0/).

OPEN ACCESS
CellPress

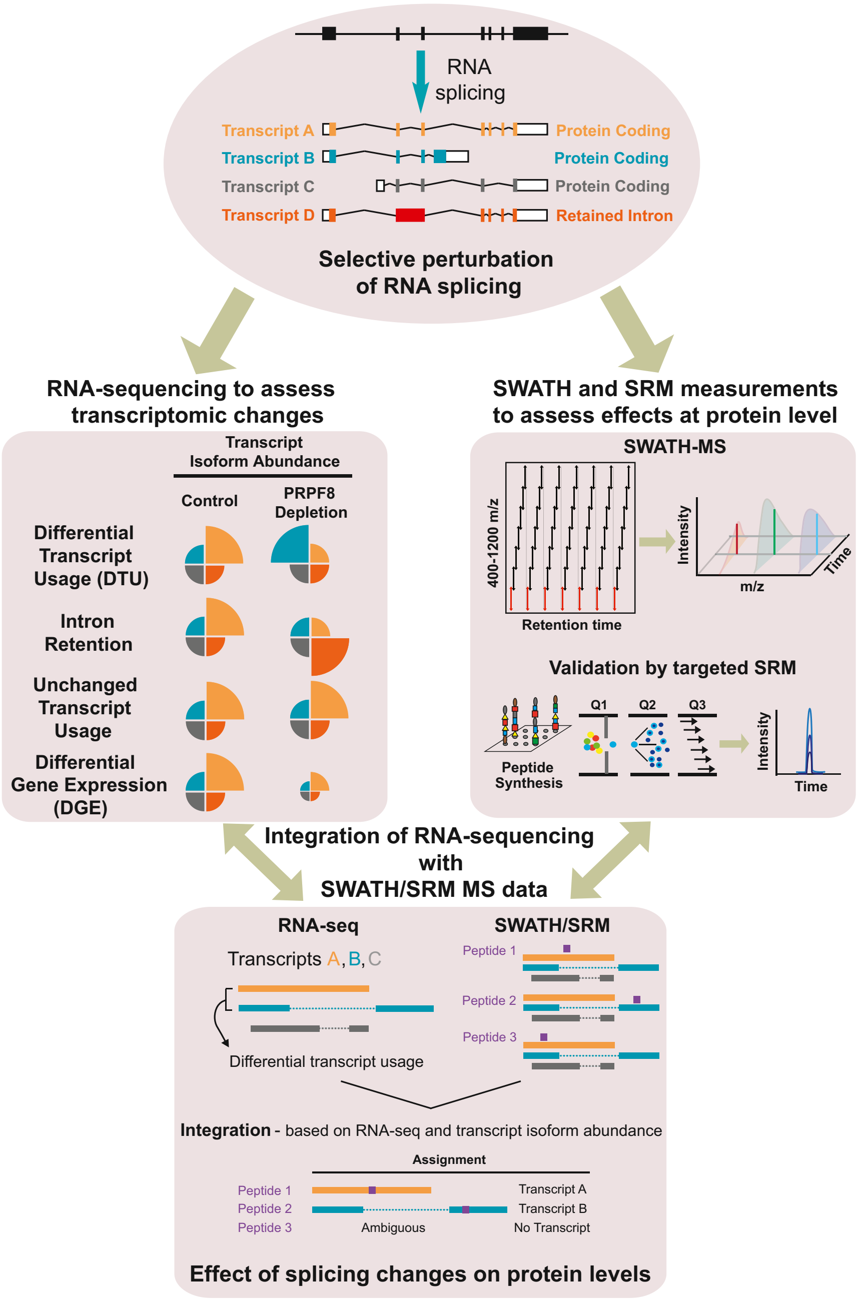

Figure 1. Framework to Study Contribution of Alternative Splicing to Proteomic Composition and Diversity

Experimental and analysis workflow. Top: RNA splicing can result in generation of multiple transcripts as indicated in this hypothetical example, including different protein coding transcripts (transcripts A–C), as well as transcripts with retained introns (transcript D). Protein coding exons are represented by solid color bars, 5′ and 3′ untranslated regions are represented by white boxes, introns are represented by black lines, and a retained intron is represented by a dark red bar. We selectively perturbed RNA splicing by depleting the core spliceosome factor PRPF8 and used RNA-seq to assess the transcriptomic changes (left) and mass spectrometry to assess the effects at the protein level (right). PRPF8 depletion can alter the relative transcript abundance of the 4 transcripts, resulting in differential transcript usage (DTU), intron retention, or unchanged transcript usage. We have defined DTU to include cases where there is a change in transcript relative abundance between conditions. Differential gene expression (DGE) may also result, where the relative transcript abundances are unchanged between conditions, but changes in expression at the gene level are observed. We used SWATH-MS (sequential window acquisition of all theoretical spectra) to assess the effects at the protein level, which were validated by targeted SRM (selective reaction monitoring). We integrated the complete proteomic dataset based on knowledge from our RNA-seq experiments in order to guide the peptide assignments (bottom panel). Because peptide 2 maps uniquely to transcript B, it is assigned to transcript B. Peptide 1 maps to multiple transcripts in the same gene (A and C), but after PRPF8 depletion, the expression of only one of these transcripts is changed. The change affects the dominant expressed isoform for this gene (known as a major transcript), hence, peptide 1 is assigned to transcript A. In contrast, peptide 3 maps simultaneously to multiple differentially used transcripts and is therefore considered ambiguous, precluding assignment to any transcript.

[FPKM]) (Wickramasinghe et al., 2015). To enable the quantification of a large fraction of the proteome with high accuracy, we used a recently developed data-independent acquisition (DIA) method, SWATH mass spectrometry (SWATH-MS), which combines the comprehensive proteome coverage of conventional shotgun proteomics with the high reproducibility and quantitative accuracy of targeted protein profiling based on SRM (selective reaction monitoring) (Gillet et al., 2012; Liu et al., 2013; Röst et al., 2014). Using SWATH-MS and the OpenSWATH software (Röst et al., 2014), we were able to identify and quantify 14,695 peptides (false discovery rate [FDR] 1%) across three biological replicates for each condition that uniquely map to 2,805 protein-encoding Ensembl genes. SWATH-MS yielded high reproducibility between technical (averaged Pearson correlation coefficient R = 0.99) and biological replicates (averaged R = 0.94) (Figure S1A). Collectively, 1,542 proteins display at least one peptide with altered protein expression levels after PRPF8 depletion (Figure S1B). Functional annotation revealed that transcripts with altered splicing patterns and proteins with altered levels are enriched in the same functional categories, namely translation, RNA splicing, mitotic cell cycle, and ubiquitination (Figure S1C). In contrast, proteins with unchanged levels after PRPF8 depletion are not enriched in these categories and are instead enriched for those involved in transcription and ribosome biogenesis (Figure S1D). Thus, significant alternative

Table 1. Alternative Integration Strategies for Differently Used Transcripts and Peptides Detected by SWATH Mass Spectrometry

Transcript Set (DTU All No.)	Peptide Set (No.)	Initial Overlap		After Assignment	Correlation Coefficient (ρ)	Agreement (%)
DTU All Transcripts and Uniquely Mapping Peptides						
		transcript	30	30	ρ 0.487	Y, 21 (70)
transcripts (452)	peptides (2,974)	peptides	65	65	p value 0.01688	N, 9 (30)
genes (388)	genes (859)	genes	30	30		
DTU All Transcripts and All Peptides						
		transcript	158	118	ρ 0.274	Y, 68 (57.63)
transcripts (452)	peptides (14,695)	peptides	700	530	p value 0.00378	N, 50 (42.37)
genes (388)	genes (2,805)	genes	128	116		
DTU Major Transcripts and Uniquely Mapping Peptides						
		transcript	27	27	ρ 0.498	Y, 20 (74.07)
transcripts (291)	peptides (2,974)	peptides	61	61	p value 0.01672	N, 7 (25.93)
genes (263)	genes (859)	genes	27	27		
DTU Major Transcripts and All Peptides						
		transcript	97	77	ρ 0.486	Y, 56 (72.73)
transcripts (291)	peptides (14,695)	peptides	481	419	p value 1.97E−05	N, 21 (27.27)
genes (263)	genes (2,805)	genes	84	75		

splicing events captured at the transcriptome level are functionally mirrored at the proteomic level.

Establishing Methods to Integrate RNA-Seq with SWATH/SRM Mass Spectrometry

To integrate the transcriptomic and proteomic datasets, we focused on identifying differential splicing events at the transcript level, which represents a major computational challenge (Kitchen et al., 2014; Low et al., 2013; Vogel and Marcotte, 2012). Previous analyses, including our own (Figure S1), have identified differential splicing events from an exon-centric perspective through mapping to the genome using DEXSeq (Anders et al., 2012). However, this approach is limited given that differential exon usage provides no information on transcript expression levels, which is expected to influence protein expression. Furthermore, differentially used exons may map to many transcripts from the same gene, making peptide assignment difficult. To overcome these limitations, we explored a transcript-centric approach, which considers transcripts as whole units, to facilitate the integration with the proteomic dataset. We first estimated transcript expression levels with MMSEQ (Turro et al., 2011) and then used its companion tool MMDIFF (Turro et al., 2014) to identify both differentially expressed genes and differentially used transcripts. Genes with differential transcript usage (DTU) are defined as cases where there is a change in the transcript relative abundances between conditions (see pie-charts in Figure 1, left panel). We identified, at high confidence, 388 genes that display DTU and 2,021 genes that display differential gene expression (DGE) following depletion of PRPF8 (Tables 1 and S3). Transcript levels of differently used transcripts were validated by RT-PCR (see Figure 5 and Wickramasinghe et al., 2015 for differently used mitotic transcripts) and genes with differential expression were validated by qRT-PCR (see Figure S5C).

We first considered the set of peptides that map uniquely to alternatively spliced transcripts involved in DTU events, defined from the mRNA data. In other words, peptide expression levels can be directly and exclusively associated with the transcripts of interest. Using this approach, we evaluated the impact at the protein level of the changes in splicing detected by RNA-seq experiments, based on the correlation between fold changes in transcript and peptide expression after PRPF8 depletion. RNA-seq fold changes were calculated from the transcript-level expression estimates obtained from MMSEQ. For each transcript, the fold change represents the median transcript expression in PRPF8-depleted versus control small interfering RNA (siRNA)-treated samples across 3 biological replicates. Peptide fold changes for each transcript were calculated by first adding up the intensities of all the peptides that mapped to that transcript in each given biological replicate and then dividing the median sum for PRPF8 depletion versus controls (hence resulting in one fold change per transcript). We observe a Spearman's correlation coefficient of 0.49 and a Pearson correlation coefficient of 0.51 when comparing fold changes in RNA and protein expression (65 peptides from 30 genes; p value = 0.0169, Spearman; p value = 0.0102, Pearson; correlation test) (Figure 2A; Table 1). Use of an alternative strategy to determine peptide fold changes for each transcript, whereby the fold change for PRPF8 depletion versus controls was determined individually for each peptide to obtain the median fold change of all peptides that mapped to that transcript, yielded similar results (see Table S2). However, uniquely mapping peptides represent a minority of cases (2,974 out of 14,665 peptides detected by SWATH-MS), because many peptides, due to their length yielded from using routine trypsin digestion and the detection range of biological mass spectrometry, are shared between transcript isoforms.

Integration of Complete SWATH Proteomic Dataset

To integrate the complete proteomic dataset, we devised a strategy that takes advantage not only of the information from peptides that map uniquely to one transcript isoform, but also from

OPEN ACCESS
CellPress

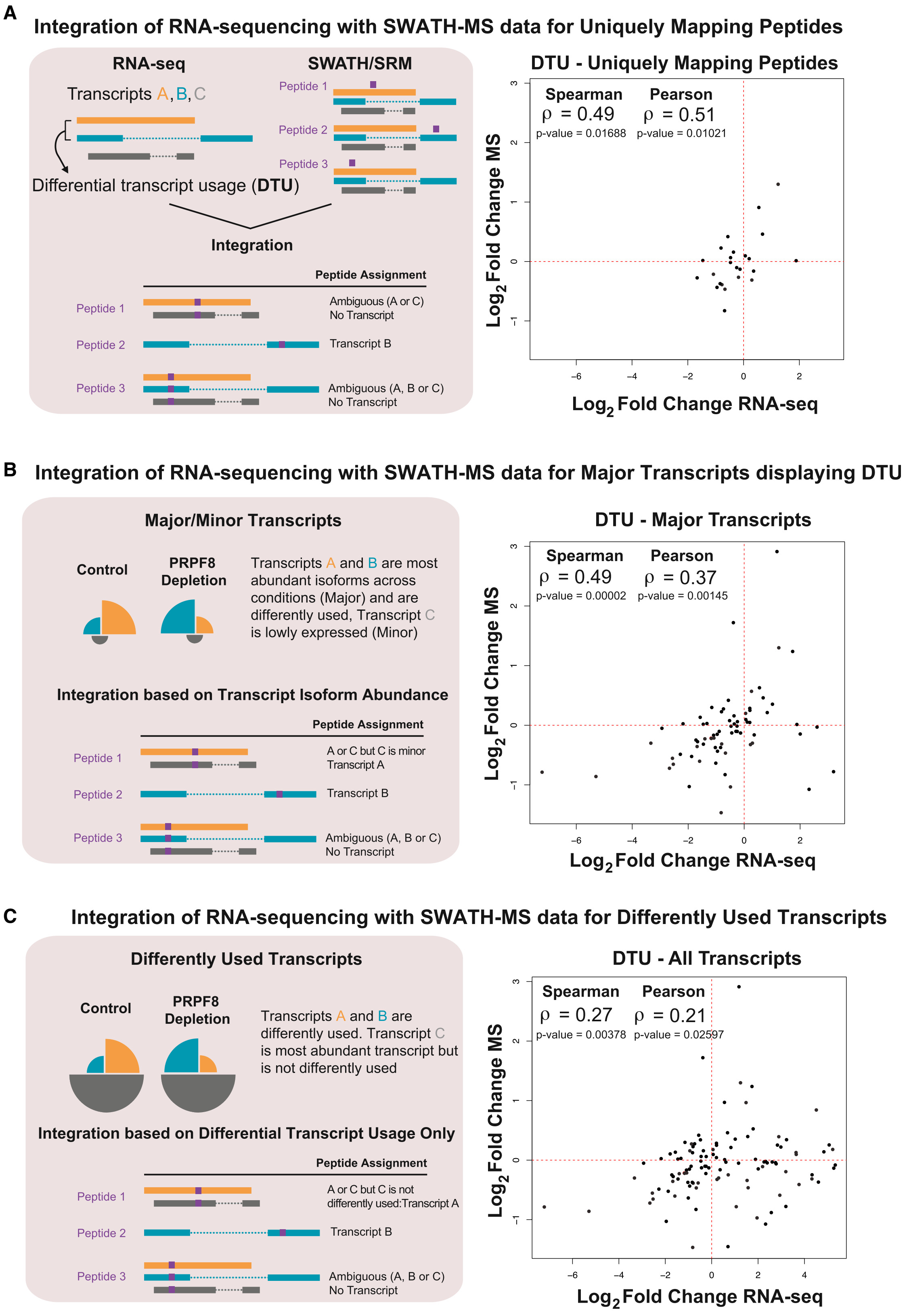

(legend on next page)

those that map to several transcripts of the same gene (Figure 2B). To do this, we used information from our RNA-seq experiments to guide the peptide assignments. More specifically, for genes with multiple isoforms, many are expressed at an extremely low level in comparison to the most abundant isoforms (Gonzàlez-Porta et al., 2013) (Figure 3A). Such a low level of mRNA expression is unlikely to manifest itself as expressed protein product within the dynamic range of the mass spectrometric method used that is ~4.4 orders of magnitude (Figure 3A). Consequently, for each gene, we considered only the most highly expressed transcript in each condition (major transcript) for peptide assignment, discarding cases where DTU did not arise in one of these. Using this criterion, we identified 263 genes whose major transcript displayed DTU (Table 1). In some cases, the identity of the major transcript differs between conditions (as discussed below), whereupon we determined separately for each major transcript whether there was evidence of differential usage following depletion of PRPF8. Subsequently, we used the regions that distinguished these two transcripts to uniquely allocate peptides (Figure 2B).

This approach yields peptide fold change information for 419 peptides corresponding to 75 genes that display DTU. Comparing mRNA fold changes with protein expression using this dataset yields a Spearman's correlation coefficient of 0.49 and a Pearson correlation coefficient of 0.37 (Figure 2B; p value = 1.97 × E−05, Spearman; p value = 0.0015, Pearson; correlation test) (Table 1). Importantly, this correlation coefficient is broadly similar to that obtained with uniquely mapping peptides across all isoforms, but using a significantly larger dataset (419 peptides from 75 genes versus 65 peptides from 30 genes). Nevertheless, most weakly expressed major transcripts displaying DTU are undetectable as expressed protein product within the dynamic range of mass spectrometry (Figure 3B). When we focus on major transcripts and uniquely mapping peptides, we observe a Spearman's correlation coefficient of 0.50 and a Pearson correlation coefficient of 0.52 (61 peptides from 27 genes; p value = 0.0167, Spearman; p value = 0.0117, Pearson; correlation test) (Figure S2). In contrast, use of an alternative integration strategy that assigns peptides to all differently used transcripts regardless of their expression levels resulted in an increase in the dataset size (530 peptides corresponding to 116 genes that display DTU) but a sharp decrease in Spearman's correlation coefficient to 0.27 and to 0.21 for Pearson (p value = 0.0378, Spearman; p value = 0.0260, Pearson; correlation test) (Figure 2C; Table 1). This result suggests that the inclusion of minor transcripts with low expression levels increases noise at both the mRNA and protein level, making reliable peptide assignment difficult (Figure 3). Consequently, this indicates that usable information can be obtained from peptides that map to more than one transcript in the same gene only if information on transcript abundance is considered. Taken together, these results suggest that transcript expression levels play a dominant role in regulating protein abundance, which supports the idea that differential splicing events in minor transcripts correspond to subtle changes that do not have a strong impact on the overall proteome, whatever their functional outcome.

Validation Using Selective Reaction Monitoring Mass Spectrometry

To validate our findings using a more sensitive mass spectrometric approach, we performed selective reaction monitoring (SRM) on control siRNA-treated and PRPF8-depleted samples (Figures S3A and S3B). To increase the quantitative precision, we spiked heavy isotope-labeled peptide standards for SRM measurement into the sample. SRM has a higher sensitivity but a much lower analyte throughput than SWATH; hence, we were only able to determine peptide fold change information for 53 targeted peptides corresponding to 15 genes whose major transcripts display DTU. Comparing mRNA fold changes with protein expression using this dataset yields a Spearman's correlation coefficient of 0.62 and a Pearson correlation coefficient of 0.59 (p value = 0.0116, Spearman; p value = 0.01663, Pearson; correlation test) (Figure 4B; Table S1). When considering only peptides that map uniquely to transcripts involved in DTU events, we observe an increased correlation coefficient of 0.78 (0.71 for Pearson) (35 peptides from 13 genes; p value = 0.0017, Spearman; p value = 0.0043, Pearson; correlation test) (Figure 4A; Table S1) and 0.73 (0.70 for Pearson) when focusing on major transcripts and uniquely mapping peptides (33 peptides from 12 genes; p value = 0.0063, Spearman; p value = 0.00794, Pearson; correlation test) (Figure S3C; Table S1). Collectively, our findings demonstrate that changes in isoform usage across the human transcriptome manifest at the proteome level.

Biological Impact of Functional mRNA Isoforms through Proteome Diversity

Alternative splicing has the potential to vastly increase the diversity of proteins encoded by the human genome. To assess

Figure 2. Changes in Isoform Usage Manifest Themselves at the Proteome Level

(A) Uniquely mapping peptides and SWATH-MS. Schematic indicating peptide to transcript mapping for uniquely mapping peptides is shown on left. Scatterplot comparing changes in expression of differently used transcripts (DTU) ($\log_2$ fold change RNA-seq) to changes in expression of the peptides that uniquely map to them ($\log_2$ fold change SWATH-MS) after PRPF8 depletion is shown on right. Spearman and Pearson correlation coefficients and associated p values are shown in top left corner.

(B) Major transcripts and SWATH-MS. Schematic indicating peptide to transcript mapping for major transcripts is shown on left. Similar scatterplot is shown on right for transcripts whose most highly expressed isoform (major transcript) changes in expression after PRPF8 depletion with corresponding peptide evidence.

(C) Use of an alternative integration strategy for peptide assignment where information about transcript expression levels was not considered increases dataset size but reduces correlation coefficient. Specifically, if a peptide maps to multiple transcripts in the same gene, but the expression of only one of these transcripts was changed after PRPF8 depletion, then this peptide was assigned to that particular transcript regardless of its expression level. In contrast, peptides that map simultaneously to multiple differentially used transcripts were considered ambiguous and were not used for further analysis. A scatterplot comparing changes in expression of differently used transcripts (DTU) ($\log_2$ fold change RNA-seq) to changes in expression of their corresponding peptides ($\log_2$ fold change SWATH-MS) after PRPF8 depletion is shown. Spearman and Pearson correlations coefficient and associated p values are shown in top left corner.

See also Figure S2 and Table 1.

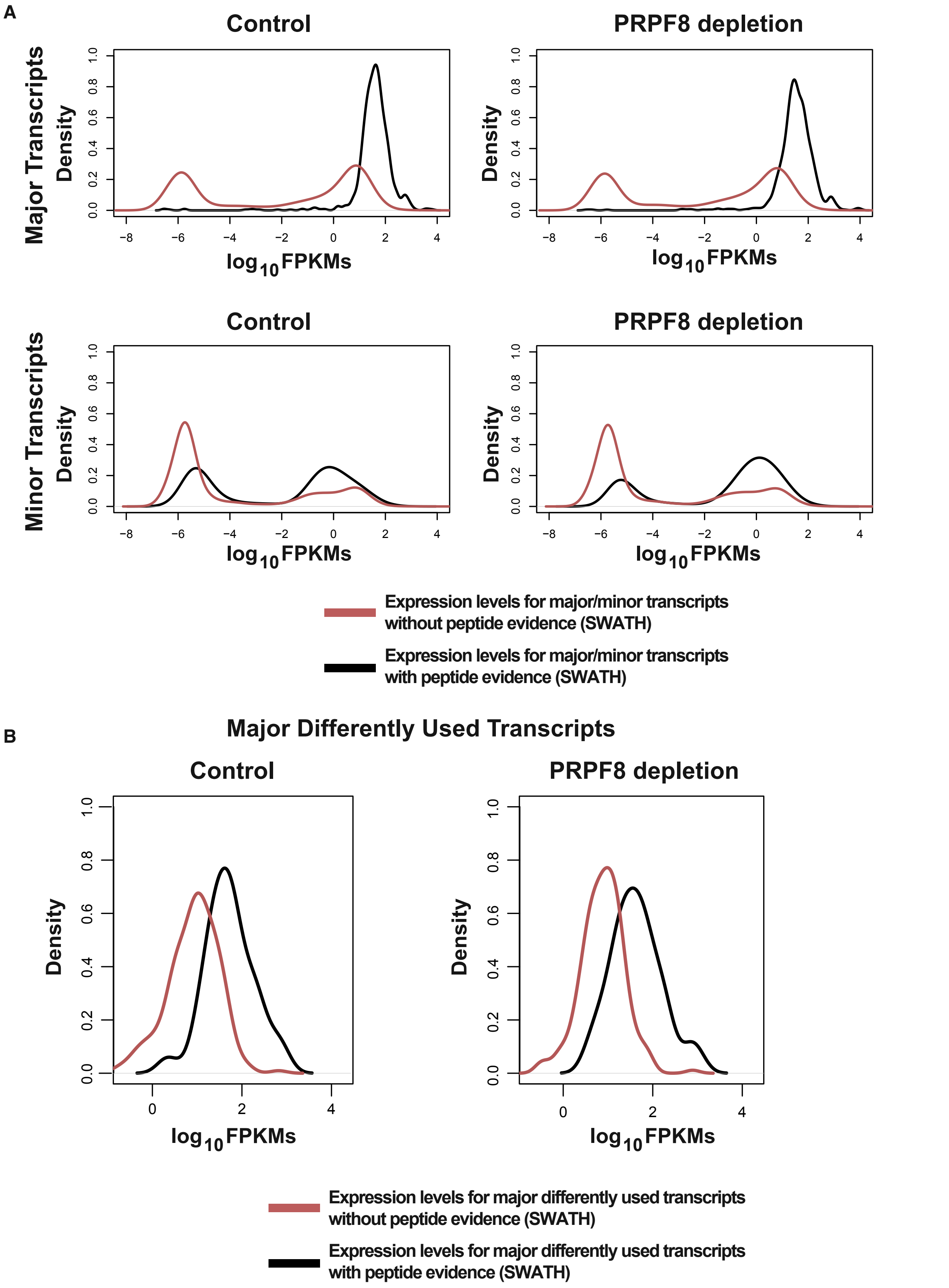

(legend on next page)

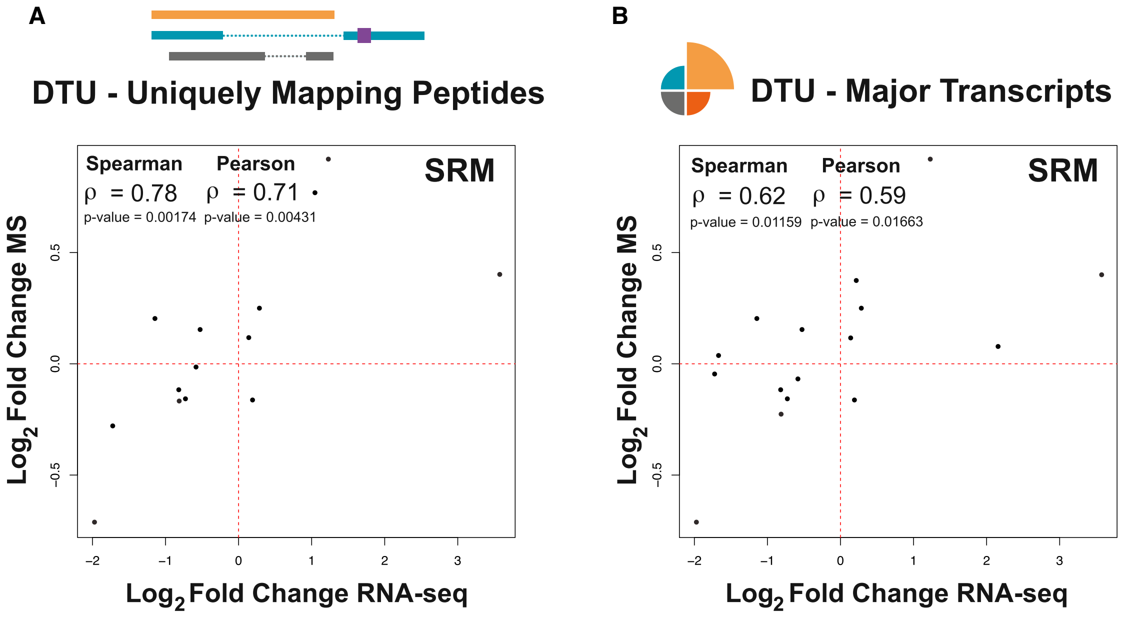

Figure 4. Validation Using Selective Reaction Monitoring Mass Spectrometry (SRM)

(A) Uniquely mapping peptides and SRM. Scatterplot comparing changes in expression of differently used transcripts (DTU) (log_2 fold change RNA-seq) to changes in expression of the peptides that uniquely map to them (log_2 fold change SRM-MS) after PRPF8 depletion. Peptide expression information was obtained using SRM.

(B) Major transcripts and SRM. Similar scatterplot is shown for transcripts whose most highly expressed isoform (major transcript) changes in expression after PRPF8 depletion with corresponding peptide evidence.

See also Figure S3C and Table S2.

whether the changes in alternative splicing that we observe at the protein level may have a functional impact on cellular biology, we focused on extreme examples of alternative splicing, where the identity of major transcripts changes across conditions. This is termed a switch event (Gonzàlez-Porta et al., 2013) and two examples that result in changes in protein isoform expression as determined by SWATH and SRM are shown in Figures 5 and S4 (LAP2 and hnRNPK). We focused on lamin-associated polypeptide (LAP2), also known as thymopoietin, because its various isoforms have been functionally characterized in detail (Dechat et al., 1998; Somech et al., 2005). LAP2 undergoes a switch event after PRPF8 depletion, whereby the dominant isoform changes from LAP2β to LAP2α, whose N-terminal region of 187 amino acids (encoded by exons 1–3) is shared with LAP2β (Figures 5A and S5A). Changes in protein expression of each isoform, consistent with the change observed at the mRNA level, were determined by SWATH-MS and validated by SRM. Thus, one peptide shared by both isoforms did not change in expression after PRPF8 depletion, whereas peptides uniquely mapping to LAP2β decreased and those to LAP2α increased respectively (Figures 5B and S5B). These changes were confirmed at the RNA (Figure 5C) and protein (Figure 5D) levels using probes and antibodies that recognize each specific isoform.

Both isoforms have different cellular locations and functions: LAP2β localizes to the nuclear lamina and represses transcription of p53 and nuclear factor κB (NF-κB) target genes (Dechat et al., 1998; Somech et al., 2005), while LAP2α is localized throughout the nuclear interior and is implicated in the structural organization of the nucleus (Dechat et al., 1998). In unperturbed cells, the majority of LAP2 protein localizes to the nuclear lamina, corresponding to the LAP2β isoform (Figure 5E). Following PRPF8 depletion, this staining pattern is reversed: less LAP2 protein is observed at the nuclear lamina, and more is observed in the nuclear interior, corresponding to increased levels of the LAP2α isoform (Figure 5E). Consistent with a reduction in LAP2β levels, we observe a de-repression of direct p53 and NF-κB transcriptional targets after PRPF8 depletion (Figure S5C),

Figure 3. Peptides Encoded by Major Transcripts Are More Frequently Detected by SWATH-MS

(A) Expression levels (log_{10} FPKMs [fragments per kilobase of transcript per million mapped reads]) of major and minor transcripts with or without peptide evidence are indicated for Control and PRPF8-depleted samples. Only uniquely mapping peptides were used for this analysis and one biological replicate for each condition is shown.

(B) Lowly expressed major transcripts displaying DTU are not detectable as expressed protein product within dynamic range of SWATH mass spectrometry. Expression levels (log_{10} FPKMs) for major transcripts displaying DTU with or without peptide evidence are indicated for Control and PRPF8-depleted samples. One biological replicate for each condition is shown.

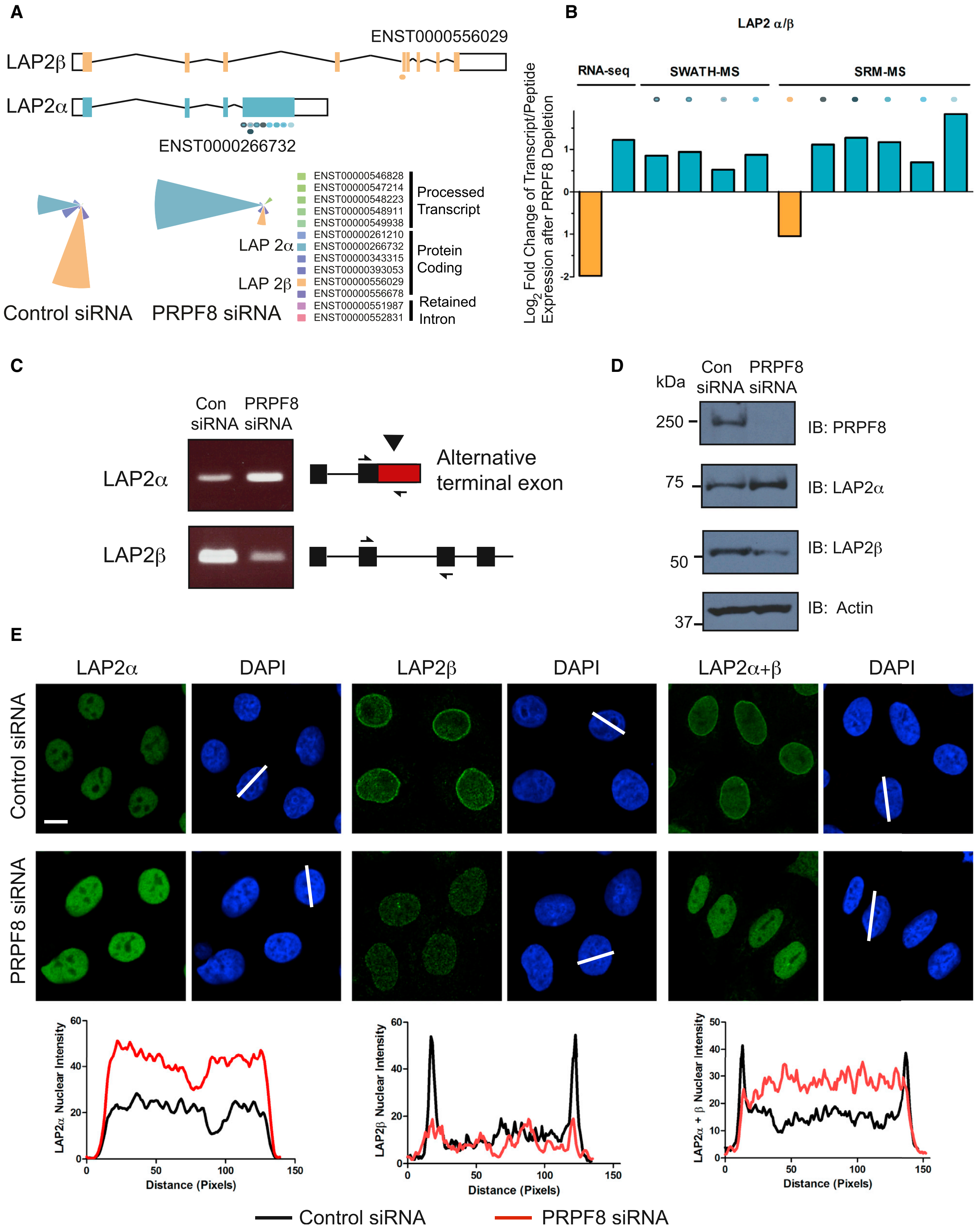

(legend on next page)

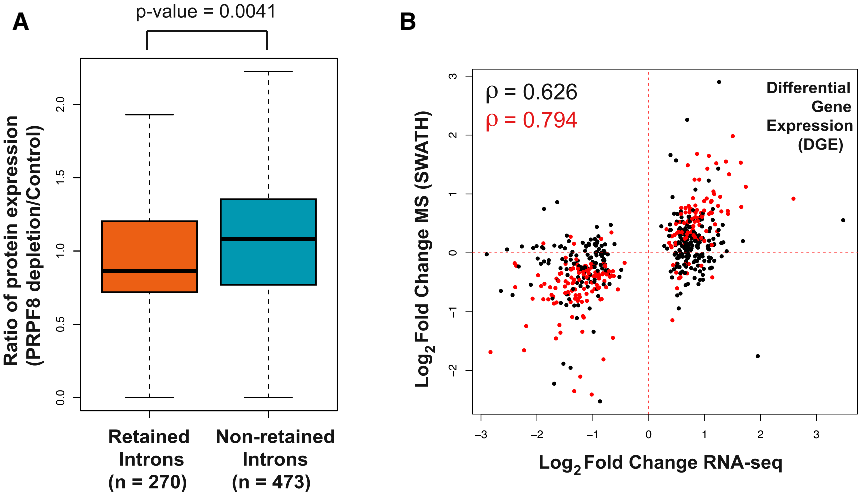

Figure 6. Intron Retention and Differential Gene Expression Functionally Tune the Human Proteome

(A) Intron retention reduces protein levels. Boxplot representing the ratio of protein expression (PRPF8 depletion/Control) is shown for retained introns (n = 270) and non-retained introns (n = 473) with peptide evidence. p value is indicated (Wilcoxon test).

(B) Alterations in gene expression alter protein abundance proportionally to transcript levels. Scatterplot comparing changes in expression of differentially expressed genes (DGE) ($\log_2$ fold change RNA-seq) to changes in expression of the peptides that map to them ($\log_2$ fold change SWATH-MS) after PRPF8 depletion. Spearman's correlation coefficient is shown in top left corner. Differently expressed genes whose corresponding peptides change significantly in expression (adjusted p value < 0.1, t test, Holm method) are indicated in red and associated correlation coefficient is also shown in red.

See also Figure S6 and Table S3.

highlighting the potential biological impact of functionally relevant mRNA isoforms by the quantitative modulation of their respective protein isoforms.

Intron Retention Reduces Protein Levels

Intron retention is a specific form of alternative splicing that is increasingly regarded as a regulatory event that can control gene expression (Kalyna et al., 2012; Wong et al., 2013; Yap et al., 2012). We therefore assessed the impact of intron retention on the composition of the proteome. Recent findings have suggested that intron retention affects transcripts from as many as 75% of multi-exon genes (Braunschweig et al., 2014). Transcripts with retained introns may not be translated because they are retained in the nucleus as they are not competent for export or may contain a premature termination codon (PTC) that results in their degradation by the nonsense-mediated decay (NMD) pathway. Consistent with this hypothesis, intron retention leading to NMD has a significant impact on transcript levels (Braunschweig et al., 2014). However, the effect of retained introns on protein expression has not been examined to date using a systematic approach covering the transcriptome. Following PRPF8 depletion, we see an increase in the expression levels of intronic reads throughout the genome (p value $< 2.2 \times 10^{-16}$) (Wickramasinghe et al., 2015). We obtained peptide evidence for 270 genes that display retained introns (identified using DEXSeq [Anders et al., 2012], see the Experimental Procedures) following PRPF8 depletion and asked whether protein expression is downregulated in these genes compared to those without intron retention. We find that the expression of their encoded proteins is reduced in comparison to those that do not display retained introns (n = 473, p value = 0.0041, Wilcoxon test) (Figure 6A). Furthermore, the proportion of downregulated proteins is higher in the group of genes with retained introns compared to those without intron retention (161/270 versus 231/473; p value = 0.0048, odds ratio: 1.547).

The relative abundance of protein-coding transcripts for each gene also has a significant effect on protein expression. Indeed, when considering genes with at least one transcript displaying a retained intron biotype, the encoded proteins that are downregulated after PRPF8 depletion have a higher relative abundance of transcripts that are not protein-coding (i.e., display intron retention) for each gene in comparison to those whose proteins are upregulated (p value = 0.0098) (Figure S6A). One example is shown in Figures S4C and S4D, where the dominant protein-coding isoform downregulated after PRPF8 depletion is replaced by an isoform with a retained intron, resulting in a decrease in protein expression (according to 5 peptides detected by SWATH-MS with average PRPF8 depletion/control

Figure 5. Biological Impact of Functional mRNA Isoforms

(A) Transcript representation of LAP2 isoforms and starplots of transcript relative abundance in control siRNA-treated and PRPF8-depleted cells. One biological replicate is shown.

(B) LAP2 switch event with corresponding peptide evidence. Column plots show fold change in expression of transcripts (left two columns) and peptides (right columns) after PRPF8 depletion. A negative fold change is represented in yellow (LAP2β), and a positive fold change in turquoise (LAP2α). Each peptide detected by SWATH or SRM is shown individually, and the region of the transcript to which it maps is represented in (A) by different colored ovals.

(C and D) Changes in LAP2 isoform expression are confirmed at the RNA (C) and protein (D) levels using probes and antibodies that recognize each specific isoform.

(E) LAP2 isoform localization is altered after PRPF8 depletion. Immunofluorescence of LAP2β and LAP2α isoforms is shown in control siRNA-treated and PRPF8-depleted Cal51 cells using antibodies that recognize each specific isoform and both isoforms respectively (scale bar, 5 μm). Scanning analysis of LAP2 isoform intensity is also shown with the scanning axes indicated by white lines. Pairs of nuclei of same scan width as determined by DAPI were used for scanning using ImageJ (NIH). Experiments in (C)–(E) were replicated independently 3 times and one representative experiment is shown.

See also Figures S4 and S5.

OPEN
ACCESS
CellPress

fold change of −0.575, p value = 0.018). Interestingly, for some genes with intron retention after PRPF8 depletion, their corresponding protein expression levels are unchanged or even increased. This suggests a complex model whereby compensation mechanisms may be at play. However, the mere detection of a transcript with a retained intron bio-type by RNA-seq may not affect the levels of the protein encoded by that gene, unless it is expressed at a robust level. Indeed, for those proteins that are upregulated after PRPF8 depletion, the median protein coding transcript relative abundance is >0.9 (Figure S6). In other words, <10% of the transcripts that make up that gene display intron retention, which may explain why these transcripts have no effect on protein level. Collectively, these results suggest that intron retention functionally tunes the human proteome as well as the transcriptome.

Alterations in Gene Expression Alter Protein Abundance Proportionally to Transcript Levels

Protein abundance is a direct determinant of cellular function and is heavily influenced by transcript levels. However, the quantitative contribution of mRNA abundance to protein abundance remains controversial (Cheng et al., 2016; Jovanovic et al., 2015; Kristensen et al., 2013; Li et al., 2014; Liu et al., 2016; Robles et al., 2014; Schwanhäusser et al., 2011; Vogel et al., 2010; Vogel and Marcotte, 2012). Given that steady-state mRNA and protein abundance are controlled by a number of post-transcriptional and translational regulatory processes (Vogel and Marcotte, 2012), establishing a correlation between the transcriptome and the proteome is not straightforward. A number of studies have used advances in next-generation sequencing and proteomics to examine the correlation between mRNA and protein abundances under steady-state conditions. Generally, the results have indicated that although there is a strong correlation between mRNA and protein abundance, a substantial proportion of the variation in protein abundance cannot be attributed to mRNA expression alone (Fu et al., 2009; Ghazalpour et al., 2011; Lundberg et al., 2010; Nagaraj et al., 2011; Schwanhäusser et al., 2011). This may be due to technical or experimental noise, along with limitations of the timescale in the experimental design and data modeling approaches (Liu et al., 2016). In contrast, more recent studies using advanced technical measurements in both steady-state and perturbed conditions have suggested that changes in mRNA abundance play a dominant role in determining the majority of dynamic changes in protein levels (Jovanovic et al., 2015; Robles et al., 2014), although this may depend on the respective contributions of mRNA and protein level regulation to the biological system being studied (Cheng et al., 2016).

We determined the contribution of changes in mRNA abundance to protein abundance using our biological system of perturbed RNA splicing. We observe 2,021 genes that are differentially expressed (DGE) after PRPF8 depletion and obtained fold change information for 3,057 peptides corresponding to 572 genes that display DGE (Table S3). We observed a Spearman's correlation coefficient of 0.63 when comparing RNA and protein fold changes in expression (Figure 6B; Table S3) that increases to 0.79 when focusing on peptides with a significant fold change (adjusted p value < 0.1, t test, Holm method) (Figure 6B; Table S3). A correlation coefficient of 0.58 (increasing to 0.76 when considering peptides with significant fold change, adjusted p value <0.1, t test, Holm method) is observed when focusing on uniquely mapping peptides (Figure S6B). Importantly, when we focus on the genes that do not display DGE, we observe a correlation coefficient of 0.29 when comparing RNA and protein fold changes in expression (Figures S6C and S6D), suggesting that changes in gene expression are driving the changes in protein expression. Taken together, these results suggest that in a system with perturbed alternative splicing, a significant proportion of the variation in protein abundance can be chiefly attributed to changes in mRNA levels.

In summary, our results illustrate how RNA splicing links isoform expression in the human transcriptome with proteomic diversity. We further show that alternative splicing events causing intron retention are accompanied by decreased protein abundance, whereas alterations in differential transcript usage and gene expression alter protein abundance proportionally to transcript levels. The fraction of the whole proteome mass of a human cell represented by the number of proteins identified in our study is very high (>99.5%) (Beck et al., 2011), suggesting that the observed events are likely to be representative for the proteome.

Our integrative analysis using a perturbed system suggests that alternative splicing events significantly contribute to both proteomic composition and diversity in humans. While a recent study that used ribosome occupancy as an indicator of translation output and not protein levels, supports our conclusions (Weatheritt et al., 2016), the contribution of alternative splicing to proteomic complexity remains divisive (Blencowe, 2017; Tress et al., 2017a, 2017b). The increase in correlation coefficient between mRNA and protein levels that we observe from SWATH to SRM suggests that a significant proportion of the protein variance from our perturbed system can be explained by differences in isoform usage. Critically, this depends on both the sensitivity of the mass spectrometric method used and the identification of high confidence alternative splicing events at the transcript level.

The methods we have developed to integrate RNA-seq and quantitative SWATH and SRM mass spectrometry data to study splicing demonstrate that usable information can be obtained from peptides that map to more than one transcript in the same gene once information on transcript abundance is considered. They provide a foundation for future studies to examine the proteome-wide effects of altered RNA splicing associated with human diseases (Kurtovic-Kozaric et al., 2015; Quesada et al., 2011; Tanackovic et al., 2011; Yoshida et al., 2011).

EXPERIMENTAL PROCEDURES

Analysis of RNA-Seq Data

The transcriptome of control siRNA-treated and PRPF8-depleted Cal51 cells was sequenced on an Illumina HiSeq2000 platform using 100 bp paired-end reads with poly(A)$^+$RNA isolated from 3 and 4 independent experiments, respectively, as previously described (Wickramasinghe et al., 2015). Raw reads were directly mapped to the transcriptome with Bowtie v0.12.7 (Langmead et al., 2009), using Ensembl v66 as a reference (Flicek et al., 2012). Following the estimation of transcript expression levels with MMSEQ v1.0.7 (Turro et al., 2011), its companion tool MMDIFF (Turro et al., 2014) was used

to identify both differentially expressed genes and differentially used transcripts as described in more detail in the Supplemental Experimental Procedures.

Protein Extraction and In-Solution Digestion

The cell pellets from three independent depletion experiments (control siRNA and PRPF8-depleted) were lysed on ice by using a lysis buffer containing 8 M urea (EuroBio), 40 mM Tris-base (Sigma-Aldrich), 10 mM DTT (AppliChem), and complete protease inhibitor cocktail (Roche) as described in more detail in the Supplemental Experimental Procedures.

Shotgun and SWATH-MS Measurement

The peptides digested from Cal51 lysate were all measured on an AB Sciex 5600 TripleTOF mass spectrometer operated in DDA mode. The same liquid chromatography-tandem mass spectrometry (LC-MS/MS) system used for DDA measurements was also used for SWATH analysis (Collins et al., 2013; Gillet et al., 2012; Liu et al., 2013) and is described in more detail in the Supplemental Experimental Procedures.

Assignment of Peptides to Transcripts

An initial set of 16,779 peptides was detected across biological replicates for each condition (control siRNA and PRPF8-depleted samples) using SWATH-MS and mapped against all the protein coding transcripts annotated in Ensembl v66, including those with a nonsense-mediated decay biotype. Removal of peptides that mapped to more than one gene led to a set of 14,695 peptides (corresponding to 2,805 genes), which was used for downstream analysis. Peptides were assigned to specific transcripts as outlined in Figure 1. Peptides that map uniquely to each transcript represented a minority of events (2,974 peptides mapping to 859 genes). Peptides that map ubiquitously to several transcripts of the same gene were assigned based on knowledge from the RNA-seq experiments using the following criteria. Two alternative peptide assignment strategies were considered. One strategy incorporated information on transcript isoform abundance for each gene into our analysis, whereby only peptides that map to major transcripts were considered. Major transcripts are the dominant expressed isoform for each gene and those identified as major in either control siRNA-treated or PRPF8-depleted samples were used specifically for peptide assignment. Additionally, we considered an alternative assignment strategy where information about transcript expression levels was not considered. Specifically, if a peptide maps to multiple transcripts in the same gene, but the expression of only one of these transcripts was changed after PRPF8 depletion, then this peptide was assigned to that particular transcript regardless of its expression level. In contrast, peptides that map simultaneously to multiple differentially used transcripts were considered ambiguous and were not used for further analysis.

Integration of Transcriptomic and Proteomic Data

To integrate transcriptomic and proteomic data, fold changes in transcript and peptide expression after PRPF8 depletion were obtained from RNA-seq and SWATH or SRM mass spectrometry experiments, respectively. RNA-seq fold changes were calculated from the transcript-level expression estimates obtained from MMSEQ as described above. For each transcript, the fold change represents the median transcript expression in PRPF8-depleted versus control siRNA-treated samples.

Raw peptide intensities were first quantile-normalized in order to enable comparison across samples. For each peptide, the observed intensities across the biological replicates in each condition were summarized by using the median, and a fold change was obtained by dividing the value obtained for PRPF8-depleted and control siRNA-treated samples. Peptide fold changes for each transcript were calculated by first adding up the intensities of all the peptides that mapped to that transcript in each given biological replicate and then dividing the median value of the summed peptide signals for PRPF8 depletion versus controls (hence resulting in one fold change per transcript). The same analysis was used for both SWATH and SRM datasets. Use of an alternative strategy to determine peptide fold changes for each transcript, whereby the fold change for PRPF8 depletion versus controls was determined individually for each peptide to obtain the median fold change of all peptides that mapped to that transcript, yielded similar results (see Table S2). The fold changes derived from these two technologies were integrated as described in Figure 1. Spearman correlation was used to evaluate the relationship between transcript and peptide fold changes, as previously suggested (Maier et al., 2009). We also used Pearson correlation as a comparison.

ACCESSION NUMBERS

The accession number for the raw data of mass spectrometry measurements (SWATH-MS and shotgun) together with the input spectral library and OpenSWATH results reported in this paper is ProteomeXchange Consortium (http://proteomecentral.proteomexchange.org): PXD003278. The accession number for the RNA sequencing data reported in this paper is ArrayExpress: E-MTAB-3021.

SUPPLEMENTAL INFORMATION

Supplemental Information includes Supplemental Experimental Procedures, six figures, and three tables and can be found with this article online at http://dx.doi.org/10.1016/j.celrep.2017.07.025.

AUTHOR CONTRIBUTIONS

Y.L. performed all mass-spectrometry experiments and analyzed data with M.G.-P. and V.O.W. M.G.-P. developed informatics pipelines and analyzed data with S.S., Y.L., and V.O.W. J.C.M. contributed to the method development, wrote the paper, and supervised M.G.-P. with A.B. A.R.V. and R.A. supervised the study, analyzed data, and wrote the paper. V.O.W. conceived the study, performed, and analyzed experiments, and wrote the paper.

ACKNOWLEDGMENTS

We thank James Hadfield and members of the sequencing facility (Cambridge Institute) for RNA sequencing. We gratefully acknowledge funding from the EMBL (to M.G.-P. and J.C.M.), the NIH (U01CA152813 to Y.S.L. and R.A.), the ERC (AdG-670821 [Proteomics 4D] to R.A.), the Swiss National Science Foundation (31003A_166435 to R.A.), SystemsX.ch through project PhosphonetX-PPM (to R.A.), the UK Medical Research Council (G1001521, G1001522, and 4050551988 to A.R.V.), and the NHMRC (1127745 to V.O.W.). V.O.W. is supported by an innovation fellowship from VESKI.

Received: April 21, 2017
Revised: June 2, 2017
Accepted: July 12, 2017
Published: August 1, 2017

REFERENCES

Anders, S., Reyes, A., and Huber, W. (2012). Detecting differential usage of exons from RNA-seq data. Genome Res. *22*, 2008–2017.

Beck, M., Schmidt, A., Malmstroem, J., Claassen, M., Ori, A., Szymborska, A., Herzog, F., Rinner, O., Ellenberg, J., and Aebersold, R. (2011). The quantitative proteome of a human cell line. Mol. Syst. Biol. *7*, 549.

Blakeley, P., Siepen, J.A., Lawless, C., and Hubbard, S.J. (2010). Investigating protein isoforms via proteomics: a feasibility study. Proteomics *10*, 1127–1140.

Blencowe, B.J. (2017). The relationship between alternative splicing and proteomic complexity. Trends Biochem. Sci. *42*, 407–408.

Braunschweig, U., Barbosa-Morais, N.L., Pan, Q., Nachman, E.N., Alipanahi, B., Gonatopoulos-Pournatzis, T., Frey, B., Irimia, M., and Blencowe, B.J. (2014). Widespread intron retention in mammals functionally tunes transcriptomes. Genome Res. *24*, 1774–1786.

Brosch, M., Saunders, G.I., Frankish, A., Collins, M.O., Yu, L., Wright, J., Verstraten, R., Adams, D.J., Harrow, J., Choudhary, J.S., and Hubbard, T. (2011). Shotgun proteomics aids discovery of novel protein-coding genes, alternative

splicing, and ‘‘resurrected’’ pseudogenes in the mouse genome. Genome Res. *21*, 756–767.

Cheng, Z., Teo, G., Krueger, S., Rock, T.M., Koh, H.W., Choi, H., and Vogel, C. (2016). Differential dynamics of the mammalian mRNA and protein expression response to misfolding stress. Mol. Syst. Biol. *12*, 855.

Clark, T.A., Sugnet, C.W., and Ares, M., Jr. (2002). Genomewide analysis of mRNA processing in yeast using splicing-specific microarrays. Science *296*, 907–910.

Collins, B.C., Gillet, L.C., Rosenberger, G., Röst, H.L., Vichalkovski, A., Gstaiger, M., and Aebersold, R. (2013). Quantifying protein interaction dynamics by SWATH mass spectrometry: application to the 14-3-3 system. Nat. Methods *10*, 1246–1253.

Dechat, T., Gotzmann, J., Stockinger, A., Harris, C.A., Talle, M.A., Siekierka, J.J., and Foisner, R. (1998). Detergent-salt resistance of LAP2alpha in interphase nuclei and phosphorylation-dependent association with chromosomes early in nuclear assembly implies functions in nuclear structure dynamics. EMBO J. *17*, 4887–4902.

Ezkurdia, I., del Pozo, A., Frankish, A., Rodriguez, J.M., Harrow, J., Ashman, K., Valencia, A., and Tress, M.L. (2012). Comparative proteomics reveals a significant bias toward alternative protein isoforms with conserved structure and function. Mol. Biol. Evol. *29*, 2265–2283.

Flicek, P., Amode, M.R., Barrell, D., Beal, K., Brent, S., Carvalho-Silva, D., Clapham, P., Coates, G., Fairley, S., Fitzgerald, S., et al. (2012). Ensembl 2012. Nucleic Acids Res. *40*, D84–D90.

Fu, X., Fu, N., Guo, S., Yan, Z., Xu, Y., Hu, H., Menzel, C., Chen, W., Li, Y., Zeng, R., and Khaitovich, P. (2009). Estimating accuracy of RNA-Seq and microarrays with proteomics. BMC Genomics *10*, 161.

Ghazalpour, A., Bennett, B., Petyuk, V.A., Orozco, L., Hagopian, R., Mungrue, I.N., Farber, C.R., Sinsheimer, J., Kang, H.M., Furlotte, N., et al. (2011). Comparative analysis of proteome and transcriptome variation in mouse. PLoS Genet. *7*, e1001393.

Gillet, L.C., Navarro, P., Tate, S., Rost, H., Selevsek, N., Reiter, L., Bonner, R., and Aebersold, R. (2012). Targeted data extraction of the MS/MS spectra generated by data-independent acquisition: a new concept for consistent and accurate proteome analysis. Mol. Cell. Proteomics *11*, O111.016717.

Gonzàlez-Porta, M., Frankish, A., Rung, J., Harrow, J., and Brazma, A. (2013). Transcriptome analysis of human tissues and cell lines reveals one dominant transcript per gene. Genome Biol. *14*, R70.

Jovanovic, M., Rooney, M.S., Mertins, P., Przybylski, D., Chevrier, N., Satija, R., Rodriguez, E.H., Fields, A.P., Schwartz, S., Raychowdhury, R., et al. (2015). Immunogenetics. Dynamic profiling of the protein life cycle in response to pathogens. Science *347*, 1259038.

Kalyna, M., Simpson, C.G., Syed, N.H., Lewandowska, D., Marquez, Y., Kusenda, B., Marshall, J., Fuller, J., Cardle, L., McNicol, J., et al. (2012). Alternative splicing and nonsense-mediated decay modulate expression of important regulatory genes in Arabidopsis. Nucleic Acids Res. *40*, 2454–2469.

Kim, M.S., Pinto, S.M., Getnet, D., Nirujogi, R.S., Manda, S.S., Chaerkady, R., Madugundu, A.K., Kelkar, D.S., Isserlin, R., Jain, S., et al. (2014). A draft map of the human proteome. Nature *509*, 575–581.

Kitchen, R.R., Rozowsky, J.S., Gerstein, M.B., and Nairn, A.C. (2014). Decoding neuroproteomics: integrating the genome, translatome and functional anatomy. Nat. Neurosci. *17*, 1491–1499.

Kristensen, A.R., Gsponer, J., and Foster, L.J. (2013). Protein synthesis rate is the predominant regulator of protein expression during differentiation. Mol. Syst. Biol. *9*, 689.

Kurtovic-Kozaric, A., Przychodzen, B., Singh, J., Konarska, M.M., Clemente, M.J., Otrock, Z.K., Nakashima, M., Hsi, E.D., Yoshida, K., Shiraishi, Y., et al. (2015). PRPF8 defects cause missplicing in myeloid malignancies. Leukemia *29*, 126–136.

Lander, E.S., Linton, L.M., Birren, B., Nusbaum, C., Zody, M.C., Baldwin, J., Devon, K., Dewar, K., Doyle, M., FitzHugh, W., et al.; International Human Genome Sequencing Consortium (2001). Initial sequencing and analysis of the human genome. Nature *409*, 860–921.

Langmead, B., Trapnell, C., Pop, M., and Salzberg, S.L. (2009). Ultrafast and memory-efficient alignment of short DNA sequences to the human genome. Genome Biol. *10*, R25.

Leoni, G., Le Pera, L., Ferrè, F., Raimondo, D., and Tramontano, A. (2011). Coding potential of the products of alternative splicing in human. Genome Biol. *12*, R9.

Li, J.J., Bickel, P.J., and Biggin, M.D. (2014). System wide analyses have underestimated protein abundances and the importance of transcription in mammals. PeerJ *2*, e270.

Liu, Y., Hüttenhain, R., Surinova, S., Gillet, L.C., Mouritsen, J., Brunner, R., Navarro, P., and Aebersold, R. (2013). Quantitative measurements of N-linked glycoproteins in human plasma by SWATH-MS. Proteomics *13*, 1247–1256.

Liu, Y., Beyer, A., and Aebersold, R. (2016). On the dependency of cellular protein levels on mRNA abundance. Cell *165*, 535–550.

Lopez-Casado, G., Covey, P.A., Bedinger, P.A., Mueller, L.A., Thannhauser, T.W., Zhang, S., Fei, Z., Giovannoni, J.J., and Rose, J.K. (2012). Enabling proteomic studies with RNA-seq: the proteome of tomato pollen as a test case. Proteomics *12*, 761–774.

Low, T.Y., van Heesch, S., van den Toorn, H., Giansanti, P., Cristobal, A., Toonen, P., Schafer, S., Hübner, N., van Breukelen, B., Mohammed, S., et al. (2013). Quantitative and qualitative proteome characteristics extracted from in-depth integrated genomics and proteomics analysis. Cell Rep. *5*, 1469–1478.

Lundberg, E., Fagerberg, L., Klevebring, D., Matic, I., Geiger, T., Cox, J., Algenäs, C., Lundeberg, J., Mann, M., and Uhlen, M. (2010). Defining the transcriptome and proteome in three functionally different human cell lines. Mol. Syst. Biol. *6*, 450.

Maier, T., Güell, M., and Serrano, L. (2009). Correlation of mRNA and protein in complex biological samples. FEBS Lett. *583*, 3966–3973.

Nagaraj, N., Wisniewski, J.R., Geiger, T., Cox, J., Kircher, M., Kelso, J., Pääbo, S., and Mann, M. (2011). Deep proteome and transcriptome mapping of a human cancer cell line. Mol. Syst. Biol. *7*, 548.

Ning, K., and Nesvizhskii, A.I. (2010). The utility of mass spectrometry-based proteomic data for validation of novel alternative splice forms reconstructed from RNA-seq data: a preliminary assessment. BMC Bioinformatics *11* (*Suppl 11*), S14.

Pan, Q., Shai, O., Lee, L.J., Frey, B.J., and Blencowe, B.J. (2008). Deep surveying of alternative splicing complexity in the human transcriptome by high-throughput sequencing. Nat. Genet. *40*, 1413–1415.

Papasaikas, P., Tejedor, J.R., Vigevani, L., and Valcárcel, J. (2015). Functional splicing network reveals extensive regulatory potential of the core spliceosomal machinery. Mol. Cell *57*, 7–22.

Park, J.W., Parisky, K., Celotto, A.M., Reenan, R.A., and Graveley, B.R. (2004). Identification of alternative splicing regulators by RNA interference in Drosophila. Proc. Natl. Acad. Sci. USA *101*, 15974–15979.

Pleiss, J.A., Whitworth, G.B., Bergkessel, M., and Guthrie, C. (2007). Transcript specificity in yeast pre-mRNA splicing revealed by mutations in core spliceosomal components. PLoS Biol. *5*, e90.

Quesada, V., Conde, L., Villamor, N., Ordóñez, G.R., Jares, P., Bassaganyas, L., Ramsay, A.J., Beà, S., Pinyol, M., Martínez-Trillos, A., et al. (2011). Exome sequencing identifies recurrent mutations of the splicing factor SF3B1 gene in chronic lymphocytic leukemia. Nat. Genet. *44*, 47–52.

Robles, M.S., Cox, J., and Mann, M. (2014). In-vivo quantitative proteomics reveals a key contribution of post-transcriptional mechanisms to the circadian regulation of liver metabolism. PLoS Genet. *10*, e1004047.

Röst, H.L., Rosenberger, G., Navarro, P., Gillet, L., Miladinović, S.M., Schubert, O.T., Wolski, W., Collins, B.C., Malmström, J., Malmström, L., and Aebersold, R. (2014). OpenSWATH enables automated, targeted analysis of data-independent acquisition MS data. Nat. Biotechnol. *32*, 219–223.

Saltzman, A.L., Pan, Q., and Blencowe, B.J. (2011). Regulation of alternative splicing by the core spliceosomal machinery. Genes Dev. *25*, 373–384.

Schwanhäusser, B., Busse, D., Li, N., Dittmar, G., Schuchhardt, J., Wolf, J., Chen, W., and Selbach, M. (2011). Global quantification of mammalian gene expression control. Nature *473*, 337–342.

Sheynkman, G.M., Shortreed, M.R., Frey, B.L., and Smith, L.M. (2013). Discovery and mass spectrometric analysis of novel splice-junction peptides using RNA-seq. Mol. Cell. Proteomics *12*, 2341–2353.

Somech, R., Shaklai, S., Geller, O., Amariglio, N., Simon, A.J., Rechavi, G., and Gal-Yam, E.N. (2005). The nuclear-envelope protein and transcriptional repressor LAP2beta interacts with HDAC3 at the nuclear periphery, and induces histone H4 deacetylation. J. Cell Sci. *118*, 4017–4025.

Tanackovic, G., Ransijn, A., Thibault, P., Abou Elela, S., Klinck, R., Berson, E.L., Chabot, B., and Rivolta, C. (2011). PRPF mutations are associated with generalized defects in spliceosome formation and pre-mRNA splicing in patients with retinitis pigmentosa. Hum. Mol. Genet. *20*, 2116–2130.

Tanner, S., Shen, Z., Ng, J., Florea, L., Guigó, R., Briggs, S.P., and Bafna, V. (2007). Improving gene annotation using peptide mass spectrometry. Genome Res. *17*, 231–239.

Tress, M.L., Bodenmiller, B., Aebersold, R., and Valencia, A. (2008). Proteomics studies confirm the presence of alternative protein isoforms on a large scale. Genome Biol. *9*, R162.

Tress, M.L., Abascal, F., and Valencia, A. (2017a). Alternative splicing may not be the key to proteome complexity. Trends Biochem. Sci. *42*, 98–110.

Tress, M.L., Abascal, F., and Valencia, A. (2017b). Most alternative isoforms are not functionally important. Trends Biochem. Sci. *42*, 408–410.

Turro, E., Su, S.Y., Gonçalves, Â., Coin, L.J., Richardson, S., and Lewin, A. (2011). Haplotype and isoform specific expression estimation using multi-mapping RNA-seq reads. Genome Biol. *12*, R13.

Turro, E., Astle, W.J., and Tavaré, S. (2014). Flexible analysis of RNA-seq data using mixed effects models. Bioinformatics *30*, 180–188.

Vogel, C., and Marcotte, E.M. (2012). Insights into the regulation of protein abundance from proteomic and transcriptomic analyses. Nat. Rev. Genet. *13*, 227–232.

Vogel, C., Abreu, Rde.S., Ko, D., Le, S.Y., Shapiro, B.A., Burns, S.C., Sandhu, D., Boutz, D.R., Marcotte, E.M., and Penalva, L.O. (2010). Sequence signatures and mRNA concentration can explain two-thirds of protein abundance variation in a human cell line. Mol. Syst. Biol. *6*, 400.

Wang, E.T., Sandberg, R., Luo, S., Khrebtukova, I., Zhang, L., Mayr, C., Kingsmore, S.F., Schroth, G.P., and Burge, C.B. (2008). Alternative isoform regulation in human tissue transcriptomes. Nature *456*, 470–476.

Weatheritt, R.J., Sterne-Weiler, T., and Blencowe, B.J. (2016). The ribosome-engaged landscape of alternative splicing. Nat. Struct. Mol. Biol. *23*, 1117–1123.

Wickramasinghe, V.O., Gonzàlez-Porta, M., Perera, D., Bartolozzi, A.R., Sibley, C.R., Hallegger, M., Ule, J., Marioni, J.C., and Venkitaraman, A.R. (2015). Regulation of constitutive and alternative mRNA splicing across the human transcriptome by PRPF8 is determined by 5′ splice site strength. Genome Biol. *16*, 201.

Wong, J.J., Ritchie, W., Ebner, O.A., Selbach, M., Wong, J.W., Huang, Y., Gao, D., Pinello, N., Gonzalez, M., Baidya, K., et al. (2013). Orchestrated intron retention regulates normal granulocyte differentiation. Cell *154*, 583–595.

Xing, X.B., Li, Q.R., Sun, H., Fu, X., Zhan, F., Huang, X., Li, J., Chen, C.L., Shyr, Y., Zeng, R., et al. (2011). The discovery of novel protein-coding features in mouse genome based on mass spectrometry data. Genomics *98*, 343–351.

Yap, K., Lim, Z.Q., Khandelia, P., Friedman, B., and Makeyev, E.V. (2012). Coordinated regulation of neuronal mRNA steady-state levels through developmentally controlled intron retention. Genes Dev. *26*, 1209–1223.

Yoshida, K., Sanada, M., Shiraishi, Y., Nowak, D., Nagata, Y., Yamamoto, R., Sato, Y., Sato-Otsubo, A., Kon, A., Nagasaki, M., et al. (2011). Frequent pathway mutations of splicing machinery in myelodysplasia. Nature *478*, 64–69.

Zhou, A., Zhang, F., and Chen, J.Y. (2010). PEPPI: a peptidomic database of human protein isoforms for proteomics experiments. BMC Bioinformatics *11* (*Suppl 6*), S7.

Cell Reports, Volume *20*

Supplemental Information

Impact of Alternative Splicing
on the Human Proteome

Yansheng Liu, Mar Gonzàlez-Porta, Sergio Santos, Alvis Brazma, John C. Marioni, Ruedi Aebersold, Ashok R. Venkitaraman, and Vihandha O. Wickramasinghe

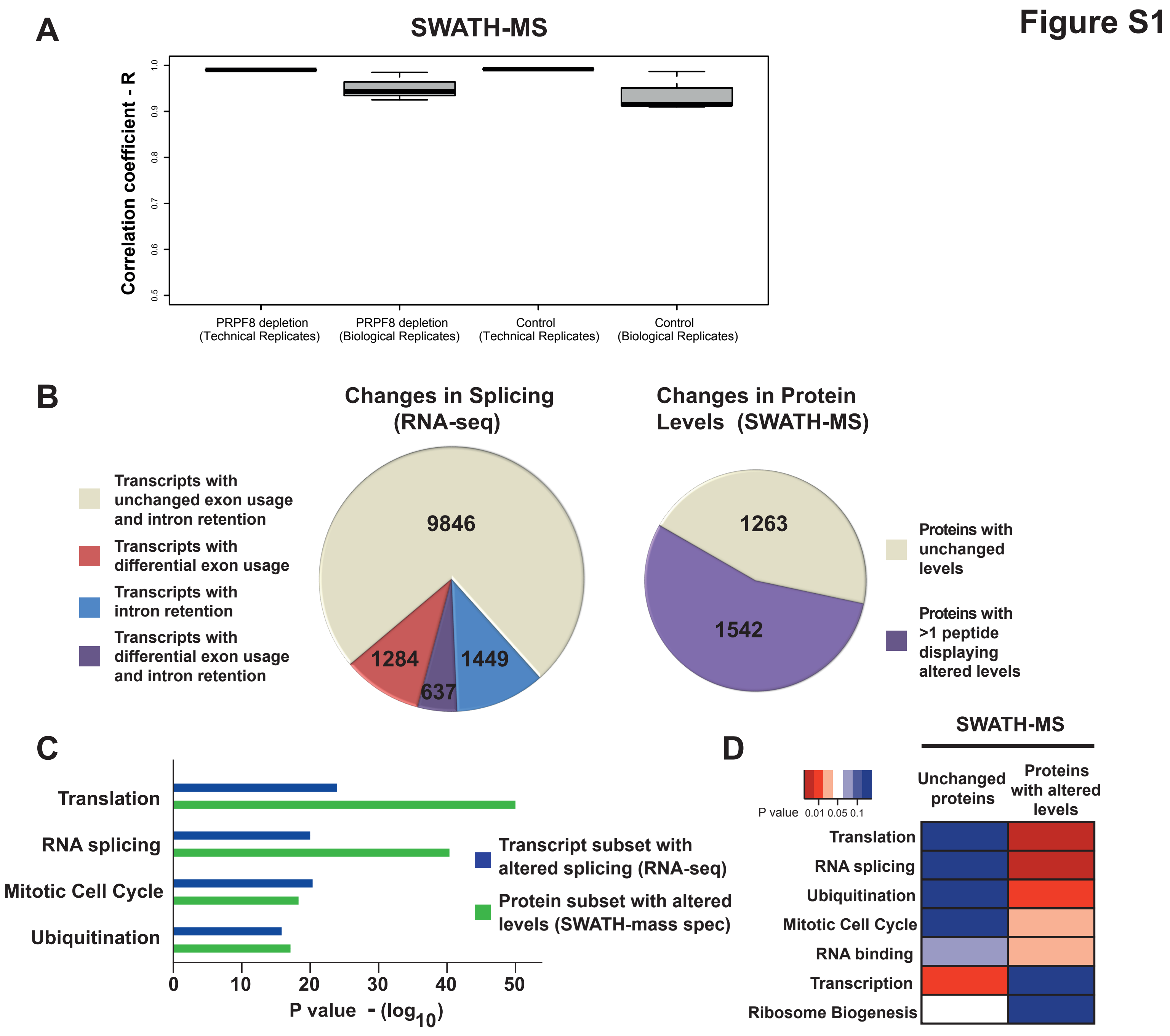

Figure S1, related to Figure 1: Transcripts with altered splicing patterns and proteins with altered levels are enriched in the same functional categories. **A**, Reproducibility of SWATH-MS data. The Pearson correlation coefficient between SWATH intensities identified and quantified from all the peptides from 3 technical replicates and 3 biological replicates for either Control or PRPF8 depleted samples analysed by SWATH-MS is indicated. **B**, Pie-chart representing proportion of transcripts with altered splicing patterns after PRPF8 depletion (differential exon usage, intron retention) as determined using DEX-seq is shown. Proportion of proteins detected by SWATH-MS with at least 1 peptide displaying altered levels after PRPF8 depletion is also indicated.
C, Functional enrichment analysis using DAVID shows that the transcript subset with altered splicing and the protein subset with altered levels are enriched for those that participate in translation, RNA splicing, mitotic cell cycle and ubiquitination. **D**, Functional enrichment analysis using DAVID with proteins detected by SWATH-MS as background shows that subset of proteins with unchanged levels after PRPF8 depletion are enriched for those involved in transcription and ribosome biogenesis. p-values are colour-coded.

Figure S2

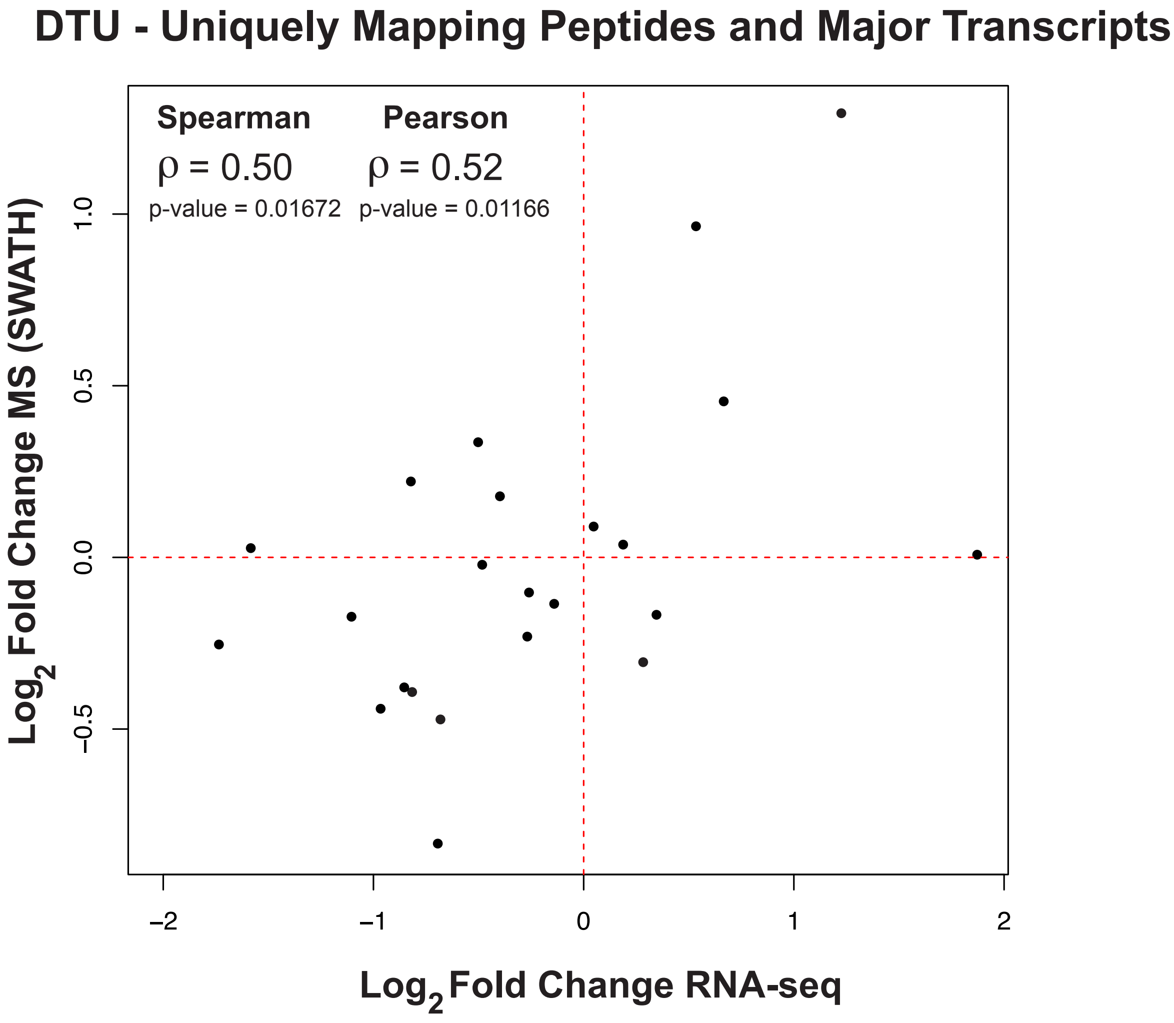

Figure S2, related to Figure 2: Correlation plot for major transcripts and uniquely mapping peptides using SWATH-MS. Scatterplot comparing changes in expression of differently used transcripts (DTU) whose most highly expressed isoform (major transcript) changes in expression (log2 fold change RNA-seq) to changes in expression of the peptides that uniquely map to them (log2 fold change SWATH-MS) after PRPF8 depletion. Spearman's correlation coefficient and p-value (correlation test) are shown in top left corner.

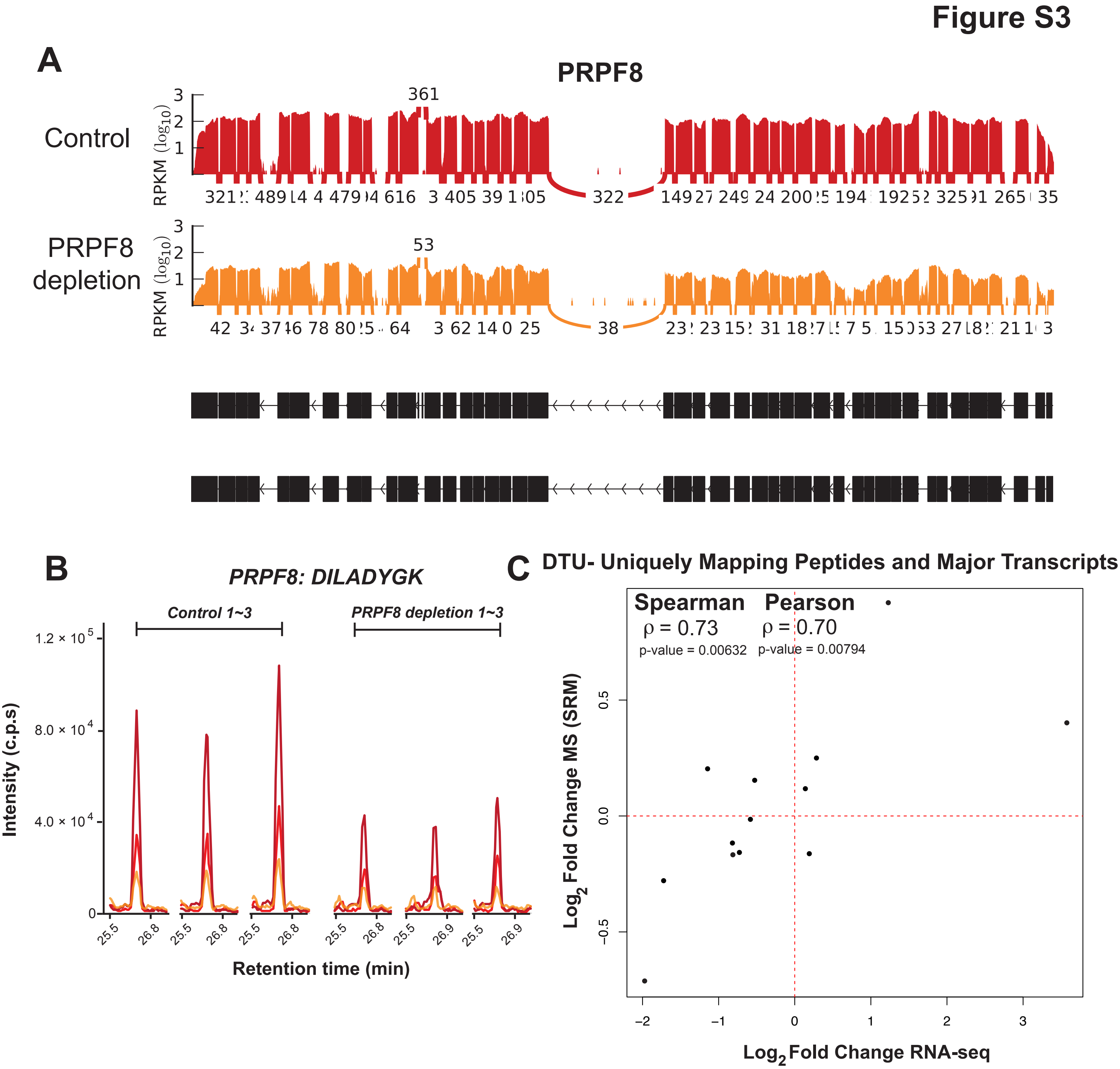

Figure S3, related to Figure 4: Validation using SRM mass spectrometry . **A**, Validation of PRPF8 depletion by RNA-sequencing. Coverage plot for the PRPF8 gene (control siRNA in red; PRPF8 siRNA in orange) obtained from RNA-sequencing data is shown. Note the reduction of reads across all exons after PRPF8 depletion. **B**, An example SRM plot for the PRPF8 peptide DILADYGK is shown for 3 biological replicates for Control and PRPF8 depleted samples. Intensity is represented on the Y-axis (c.p.s: counts per second). Efficiency of PRPF8 depletion was also verified by western blotting in Figure 5D. **C**, Correlation plot for major transcripts and uniquely mapping peptides using SRM. Scatterplot comparing changes in expression of differently used transcripts (DTU) whose most highly expressed isoform (major transcript) changes in expression (log2 fold change RNA-seq) to changes in expression of the peptides that uniquely map to them (log2 fold change SRM) after PRPF8 depletion. Spearman's correlation coefficient and p-value (correlation test) are shown in top left corner.

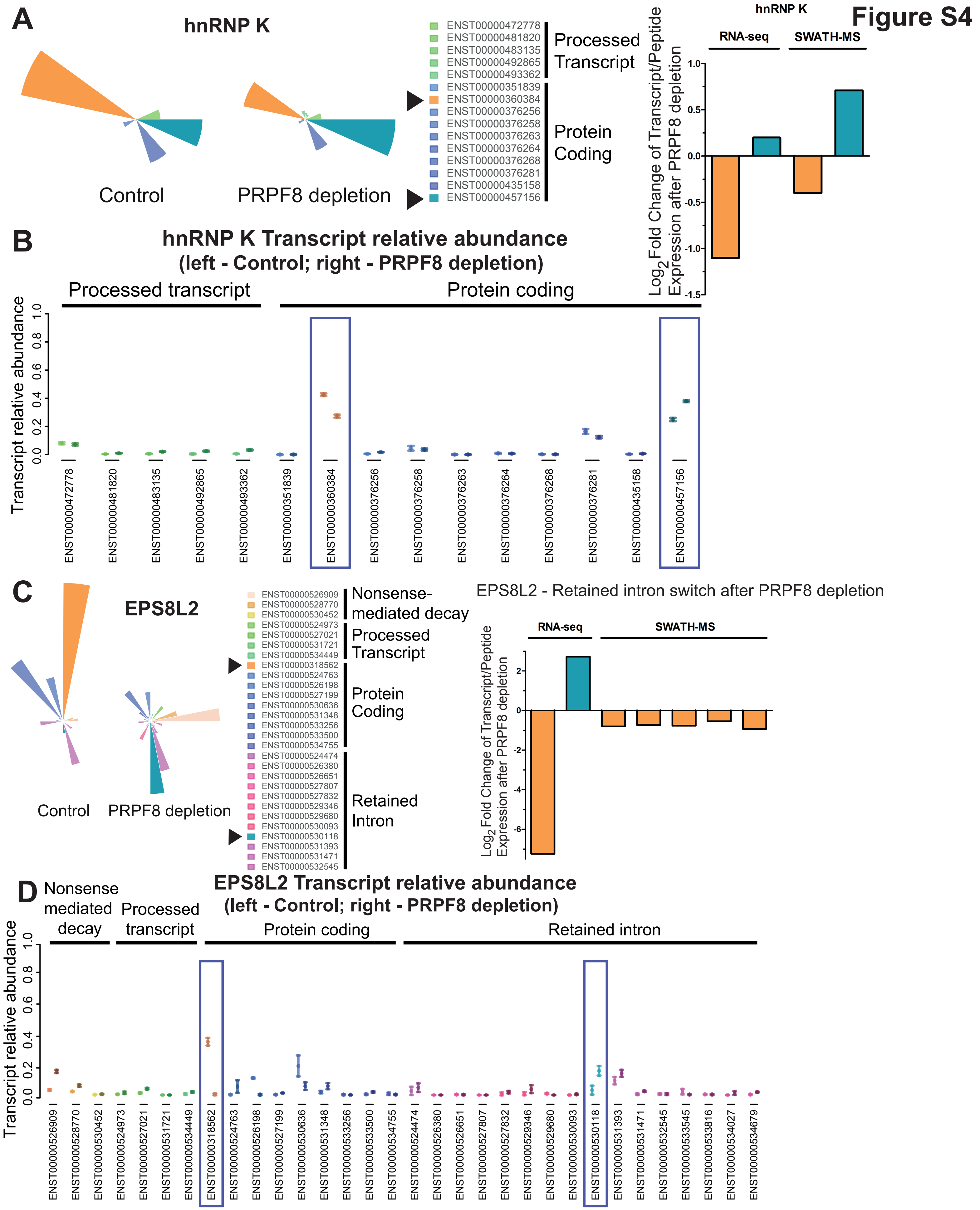

Figure S4, related to Figure 5 : Example of switch event that results in a change in protein isoform expression or a retained intron as determined by SWATH mass spectrometry. **A**, Starplot of transcript relative abundance for the hnRNPK gene is shown for control siRNA treated and PRPF8 depleted cells from one representative depletion experiment. The dominant transcript in Control cells is indicated in orange and in turquoise for PRPF8 depleted cells. Column plots show fold change in expression of these transcripts (left two columns) and their corresponding peptides (right two columns) after PRPF8 depletion as determined by SWATH-MS. **B**, For the hnRNPK gene, transcript relative abundance is also represented for each individual transcript (protein coding and processed transcripts). For each transcript, the transcript relative abundance in Control and PRPF8 depleted cells is on the left and right, respectively and represent the average from 3 independent depletion experiments. The major transcript in each condition is highlighted. **C,D** Starplot of transcript relative abundance for the EPS8L2 gene with corresponding fold change in peptide expression.

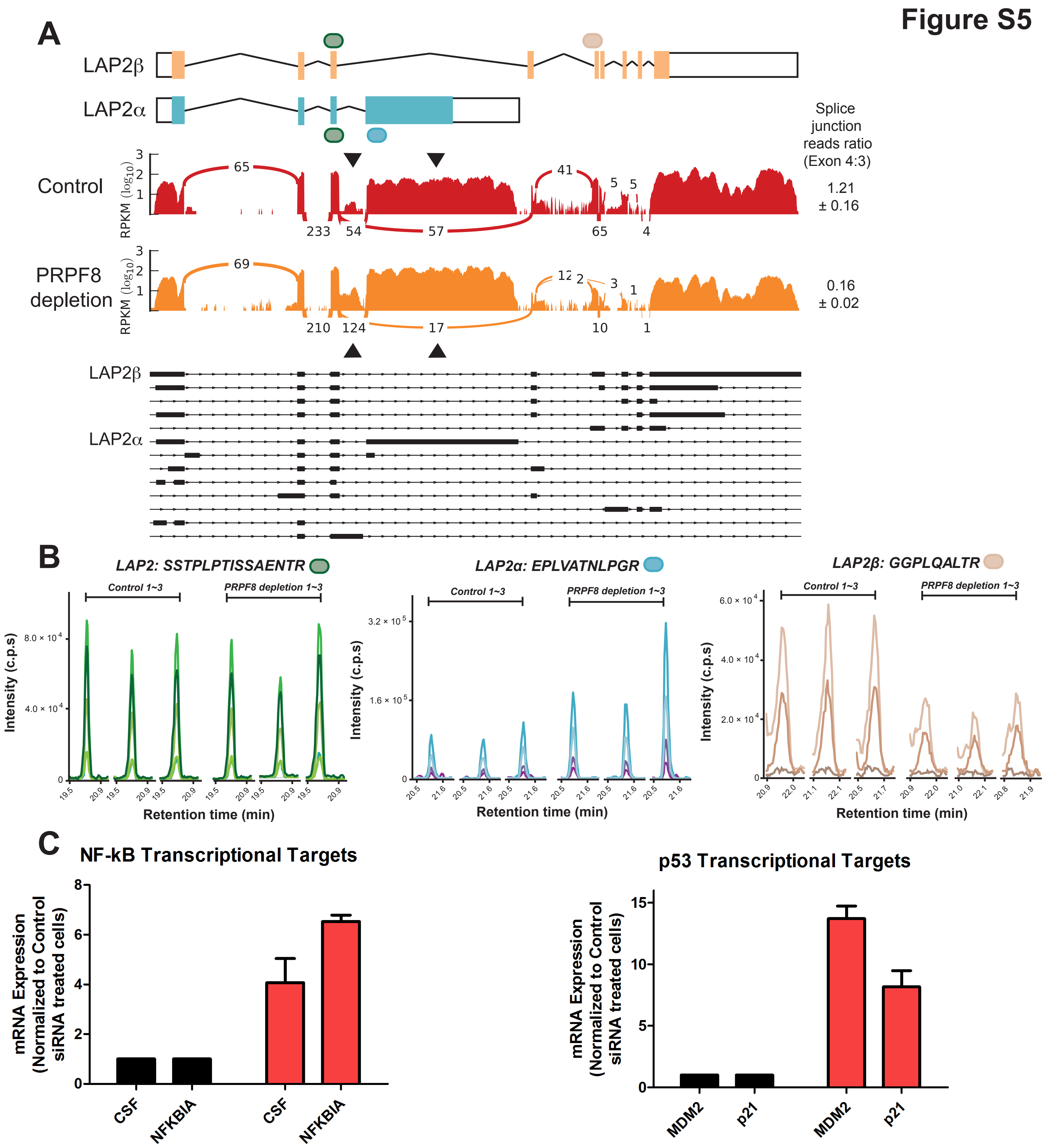

Figure S5, related to Figure 5: Validation of LAP2 switch event by SRM mass spectrometry. **A**, Coverage plot for LAP2 gene (control siRNA in red; PRPF8 siRNA in orange) obtained from RNA-sequencing data is shown. LAP2β and LAP2α isoform structures are indicated in orange and turquoise, respectively. The reduction of splice junction reads across exon 4 and 3 for the LAP2β isoform and corresponding increase for the LAP2α isoform after PRPF8 depletion is represented as a splice junction reads ratio. **B**, SRM plots for 3 peptides that map to LAP2β isoform (GGPLQALTR), LAP2α isoform (EPLVATNLPGR) and both isoforms (SSTPLPTISSAENTR) respectively are shown for 3 biological replicates for Control and PRPF8 depleted samples. Intensity is represented on the Y-axis (c.p.s: counts per second). Note that the peptide shared by both isoforms does not change in expression after PRPF8 depletion in contrast to those that map to the LAP2β and LAP2α isoforms. The quantification data was normalized by the intensity of the heavy peptide to remove run-to-run variation and the identity of the peptide was manually confirmed by the heavy isotopic peptide standard. **C**, Direct NF-kB and p53 transcriptional target genes are de-repressed after PRPF8 depletion. Consistent with a reduction in the levels of LAP2β, a known repressor of p53 and NF-kB target genes, we observe a de-repression of direct p53 and NF-kB transcriptional targets after PRPF8 depletion. For all qRT-PCR experiments in this figure, plots are relative to RNA levels in control siRNA-treated cells, assigned an arbitrary value of 1, and show the mean of triplicate readings from at least 3 independent depletion experiments, ± s.e.m. NF-kB and p53 transcriptional targets are represented in the left and right graphs, respectively.

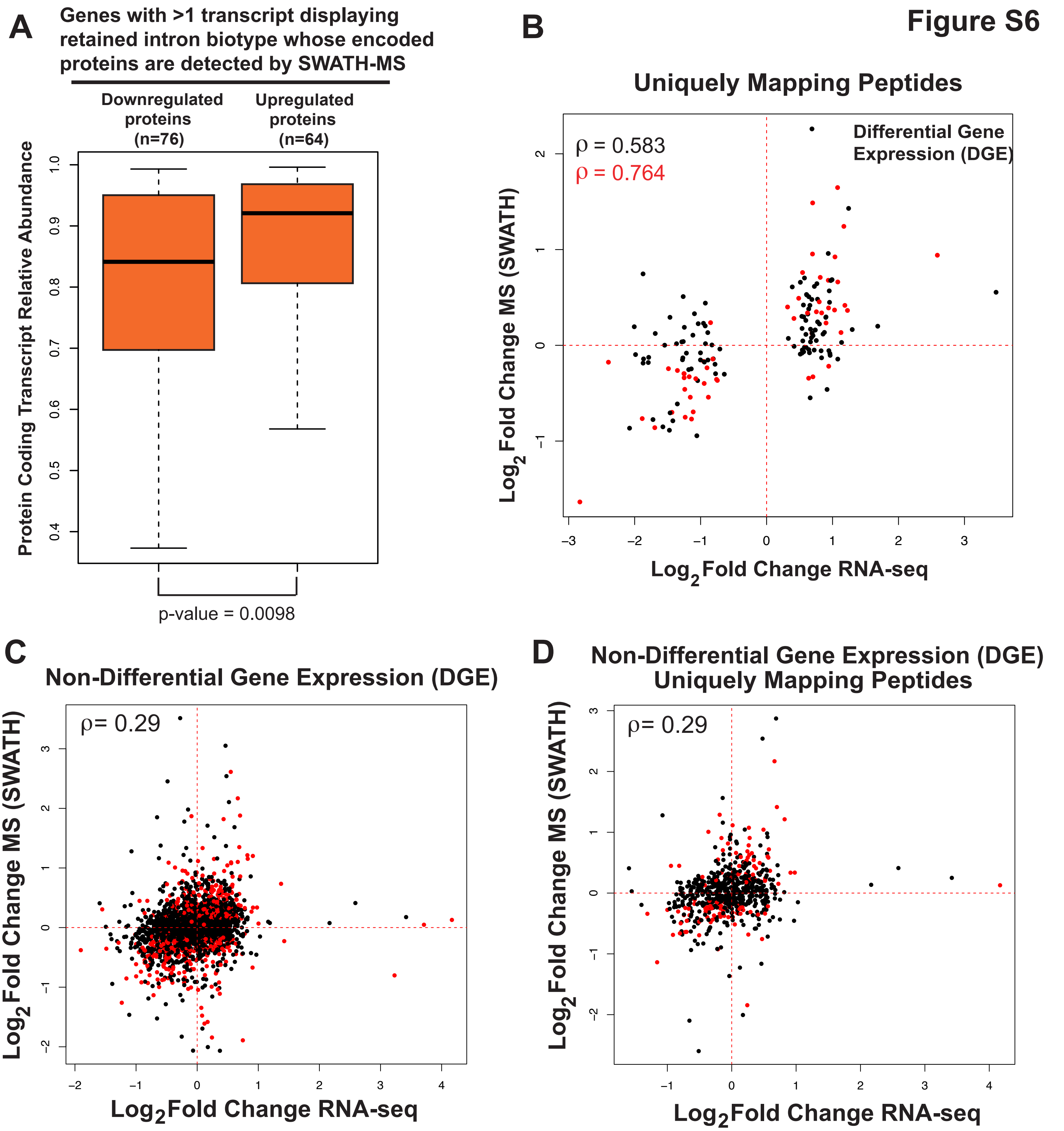

Figure S6, related to Figure 6: **A**, Relative abundance of protein-coding transcripts for each gene has a significant effect on regulating protein expression. Protein coding transcript relative abundance is shown for genes with >1 transcript displaying a retained intron biotype whose encoded proteins are detected by SWATH-MS. Boxplots representing downregulated and upregulated proteins after PRPF8 depletion are shown. Downregulated proteins have a higher relative abundance of transcripts that are not protein coding (i.e. display intron retention) in comparison to upregulated proteins and the corresponding p-value is indicated at the bottom of the boxplot (Wilcoxon test). **B**, Differential gene expression for uniquely mapping peptides. Scatterplot comparing changes in expression of differentially expressed genes (DGE) (log2 fold change RNA-seq) to changes in expression of the peptides that map uniquely to them (log2 fold change SWATH-MS) after PRPF8 depletion. Spearman's correlation coefficient is shown in top left corner. Differently expressed genes whose corresponding peptides change significantly in expression (adjusted p-value <0.1, t-test) are indicated in red and associated correlation coefficient is also shown in red. **C**, Non-differently expressed genes show a poor correlation when comparing RNA and protein fold-changes in expression after PRPF8 depletion. Scatterplot comparing changes in expression of non-differentially expressed genes (non-DGE) (log2 fold change RNA-seq) to changes in expression of the peptides that map to them (log2 fold change SWATH-MS) after PRPF8 depletion. Spearman's correlation coefficient is shown in top left corner. Differently expressed genes whose corresponding peptides change significantly in expression (adjusted p-value <0.1, t-test) are indicated in red. A similar plot for uniquely mapping peptides is shown in **D**.

DTU all transcripts + uniquely mapping peptides

transcript (tx) set DTU all #		peptide set uniquely mapping #			initial overlap	after assignment	Correlation coefficient (ρ)		agreement (%)		
				transcript	14	14	ρ	0.776	Y	11	78.57
tx	299	peptides	112	peptides	35	35	p-value	0.00174	N	3	21.43
genes	254	genes	51	genes	13	13					

DTU all transcripts + all peptides

transcript (tx) DTU all #		peptide set #			initial overlap	after assignment	Correlation coefficient (ρ)		agreement (%)		
				transcript	22	17	ρ	0.498	Y	12	70.59
tx	299	peptides	187	peptides	59	51	p-value	0.04418	N	5	29.41
genes	254	genes	70	genes	17	16					

DTU major transcripts + uniquely mapping peptides

transcript (tx) set DTU all #		peptide set uniquely mapping #			initial overlap	after assignment	Correlation coefficient (ρ)		agreement (%)		
				transcript	13	13	ρ	0.731	Y	10	76.92
tx	191	peptides	112	peptides	33	33	p-value	0.00632	N	3	23.08
genes	171	genes	51	genes	12	12					

DTU major transcripts + all peptides

transcript (tx) set DTU all #		peptide set #			initial overlap	after assignment	Correlation coefficient (ρ)		agreement (%)		
				transcript	19	16	ρ	0.624	Y	12	75.00
tx	191	peptides	187	peptides	56	53	p-value	0.01159	N	4	25.00
genes	171	genes	70	genes	16	15					

Table S1, related to Figure 4: Alternative integration strategies for differently used transcripts and peptides detected by SRM mass spectrometry

DTU all transcripts + uniquely mapping peptides			
**Correlation coefficient (SWATH)**		**Correlation coefficient (SRM)**	
ρ	0.301	ρ	0.723
**p-value**	0.110	**p-value**	0.005
**DTU all transcripts + all peptides**			
**Correlation coefficient (SWATH)**		**Correlation coefficient (SRM)**	
ρ	0.273	ρ	0.667
**p-value**	0.003	**p-value**	0.004
**DTU major transcripts + uniquely mapping peptides**			
**Correlation coefficient (SWATH)**		**Correlation coefficient (SRM)**	
ρ	0.258	ρ	0.665
**p-value**	0.211	**p-value**	0.016
**DTU major transcripts + all peptides**			
**Correlation coefficient (SWATH)**		**Correlation coefficient (SRM)**	
ρ	0.425	ρ	0.682
**p-value**	0.0002	**p-value**	0.0047

Table S2, related to Figures 2, 4 : Correlation coefficients for differently used transcripts and peptides detected by SWATH/SRM mass spectrometry using an alternative strategy to determine peptide fold-changes for each transcript

Differently Expressed Genes (DGE) using SWATH dataset											
**Uniquely mapping peptides**											
**transcript set DGE**		**peptide set uniquely mapping**	**#**		**initial overlap**	**after assignment**	**correlation**		**agreement (%)**		
	**#**	**peptides**	2974	**peptides**	594	594	**rho**	0.583	**Y**	141	76.63
**genes**	2021	**genes**	859	**genes**	184	184	**p-value**	0.0E+00	**N**	43	23.37
							**correlation** adjusted p-value <0.1		**agreement (%)**		
							**rho**	0.764	**Y**	54	93.10
							**p-value**	0	**N**	4	6.90
**All peptides**											
**transcript set DGE**		**peptide set**	**#**		**initial overlap**	**after assignment**	**correlation**		**agreement (%)**		
	**#**	**peptides**	14695	**peptides**	3057	3057	**rho**	0.626	**Y**	444	77.62
**genes**	2021	**genes**	2805	**genes**	572	572	**p-value**	0	**N**	128	22.38
							**correlation** adjusted p-value <0.1		**agreement (%)**		
							**rho**	0.794	**Y**	213	91.42
							**p-value**	0	**N**	20	8.58

Table S3, related to Figure 6: Alternative integration strategies for differently expressed genes and peptides detected by SWATH mass spectrometry

Supplemental Experimental Procedures

Cell Culture

Cal51 breast adenocarcinoma cells were a gift from Professor Paul Edwards, Department of Pathology, University of Cambridge. They were cultured in Dulbecco's Modified Eagle Medium (Invitrogen) with 10% fetal calf serum

(Invitrogen) and 1x penicillin-streptomycin (Invitrogen), and routinely tested for mycoplasma contamination.

Sample preparation

For siRNA-mediated depletion, Cal51 cells were reverse transfected with 25 nM siRNA to PRPF8 (Qiagen) using DharmaFECT1 (Dharmafect) transfection reagent, as previously described (Wickramasinghe et al., 2015). Transfected cells were harvested 54 hours later for RNA extraction and mass spectrometry from at least 3 independent depletion experiments.

Western blotting

Efficiency of depletion was monitored by western blotting with PRPF8 antibody (clone 2834C1a, ab51366, Abcam). For LAP2 isoform detection, antibodies were used that specifically recognised the α isoform (ab5162, Abcam), the β isoform (06-1002, Millipore), and both isoforms (Clone 6E10, Sigma).

RNA extraction, RT-PCR and qRT-PCR

RNA was isolated from siRNA-treated cells with an RNeasy kit (Qiagen) according to manufacturer's instructions. Isolated RNA was quantified with a NanoDrop 1000 (Thermo Scientific) and quality was determined by measuring the A_{260}/A_{280} ratio, which was always between 1.8 and 2.1, and stored at -80ºC. One μg of RNA was used for cDNA synthesis using the QuantiTect Reverse Transcription Kit (Qiagen) according to manufacturer's instructions. Synthesized cDNA was diluted following reverse transcriptase inactivation and stored at –20ºC. Primers for qPCR were designed to bridge exon-intron junctions. For RT-PCR experiments, PCR was conducted on a MJ Research thermal cycler using Accuprime Pfx DNA polymerase (Invitrogen), forward

and reverse primer and cDNA. qPCR was conducted on a Rotorgene RG-3000 (Corbett Research) machine using 2x SYBR-Green Master Mix (Roche), forward and reverse primer and cDNA. The cycling acquisition program was as follows: 50ºC 2 minutes, 95ºC 2 minutes, 50 cycles of 95ºC for 15 seconds and 60ºC for 30 seconds. The C_t values were calculated, referenced to standard curves for each primer set. All samples were then normalized to control siRNA treated samples.

Immunofluorescence

Immunofluorescence was performed as previously described (Wickramasinghe et al., 2010). Briefly, cells were fixed in 4% paraformaldehyde for 5 min at room temperature and permeabilised in PBS, 0.1% Triton X-100 (Sigma) and 0.02% SDS for 10 min at room temperature. After 30 minutes in blocking buffer (permeabilisation buffer + 1 % BSA), coverslips were incubated with the appropriate primary (α isoform - ab5162, Abcam; β isoform - 06-1002, Millipore; both isoforms - Clone 6E10, Sigma) and secondary antibodies (Molecular Probes) and examined using a Zeiss LSM510 Meta confocal microscope. Scanning analysis of cells was performed using ImageJ software (NIH). All images used for comparative analysis were acquired using identical microscope settings. A line width of 20 was used, and pairs of cells with nuclei of same scan width as indicated by DAPI staining were used for analysis. All analyses are representative of the cell population.

Analysis of RNA-sequencing data

The transcriptome of control siRNA-treated and PRPF8 depleted Cal51 cells was sequenced on an Illumina HiSeq2000 platform using 100 bp paired-end reads with poly(A)+RNA isolated from 3 and 4 independent experiments,

respectively, as previously described (Wickramasinghe et al., 2015). Raw reads were directly mapped to the transcriptome with Bowtie v0.12.7 (Langmead et al., 2009), using Ensembl v66 as a reference (Flicek et al., 2012). Following the estimation of transcript expression levels with MMSEQ v1.0.7 (Turro et al., 2011), its companion tool MMDIFF (Turro et al., 2014) was used to identify both differentially expressed genes and differentially used transcripts. MMDIFF uses Bayesian inference to evaluate the probability that two genes are differentially expressed / two transcripts are differentially used across conditions, which is termed 'posterior probability'. A posterior probability of 0.85 was used as the significance threshold for analysing the SWATH data and 0.9 for the SRM data. Switch events within the set of genes identified to undergo differential transcript usage were identified with SwitchSeq (Gonzalez-Porta and Brazma, 2015). Switch events that involved major transcripts with identical protein sequences were removed from the analyses.

Protein extraction and in-solution digestion.

The cell pellets from three independent depletion experiments (control siRNA and PRPF8 depleted) were lysed on ice by using a lysis buffer containing 8 M urea (EuroBio), 40 mM Tris-base (Sigma-Aldrich), 10 mM DTT (AppliChem) and complete protease inhibitor cocktail (Roche). The resulted mixture was sonicated at 4 °C for 5 mins using a VialTweeter device (Hielscher-Ultrasound Technology) and centrifuged at 21130 g, 4 °C for 1 hr to remove the insoluble material. The supernatant protein mixtures were transferred and protein amount was determined using a Bradford assay (Bio-Rad, Hercules, CA, USA). Aliquots of 1 mg protein mixtures were reduced by 5 mM tris(carboxyethyl)phosphine (Sigma-Aldrich) and alkylated by 30 mM

iodoacetamide (Sigma-Aldrich). Then 5 volumes of precooled precipitation solution containing 50% acetone, 50% ethanol, and 0.1% acetic acid was added to the protein mixture and kept at −20 °C overnight. The mixture was centrifuged at 20,400 *g* for 40 min. The pellets were washed with 100% acetone and 70% ethanol with centrifugation at 20,400 *g* for 40 min. The samples were then resolved by 100 mM NH_4HCO_3 and were digested with sequencing-grade porcine trypsin (Promega) at a protease/protein ratio of 1:50 overnight at 37 °C (Kim et al., 2006). Digests were purified with Vydac C18 Silica MicroSpin columns (The Nest Group Inc.). Peptide amount was determined using Nanodrop ND-1000 (Thermo Scientific) and about 0.7 µg peptide mixtures were analyzed in each LC-MS run. An aliquot of retention time calibration peptides from iRT-Kit (Biognosys) was spiked into each sample before all LC-MS analysis at a ratio of 1:30 (v/v) to correct relative retention times between runs (Escher et al., 2012).

Shotgun measurement.

The peptides digested from Cal51 lysate were all measured on an AB Sciex 5600 TripleTOF mass spectrometer operated in DDA mode. The mass spectrometer was interfaced with an Eksigent NanoLC Ultra 2D Plus HPLC system as previously described(Collins et al., 2013; Gillet et al., 2012; Liu et al., 2013). Peptides were directly injected onto a 20-cm PicoFrit emitter (New Objective, self-packed to 20 cm with Magic C18 AQ 3-µm 200-Å material), and then separated using a 120-min gradient from 2–35% (buffer A 0.1% (v/v) formic acid, 2% (v/v) acetonitrile, buffer B 0.1% (v/v) formic acid, 90% (v/v) acetonitrile) at a flow rate of 300 nL/min. MS1 spectra were collected in the range 360–1,460 m/z. The 20 most intense precursors with charge state 2–5 which exceeded 250 counts per second were selected for fragmentation, and

MS2 spectra were collected in the range 50–2,000 m/z for 100 ms. The precursor ions were dynamically excluded from reselection for 20 s.

Peptide identification and transcript mapping.

Profile-mode .wiff files from shotgun data of Cal51 cells, together with those of HEK293, LNCap, U2OS and HeLa cells included in the previously published SWATHatlas (34 runs in total, for the purpose of increasing the coverage of the transcript-centric spectral library used in this study)(Rosenberger et al., 2014) were all centroided and converted to mzML format using the Sciex Data Converter v.1.3 and converted to mzXML format using MSConvert v.3.04.238. The MS2 spectra were queried against the fasta file of Ensembl 66 appended with reversed sequence decoys (Elias and Gygi, 2007). Two types of search engines, xTandem (Falkner and Andrews, 2005) and Omssa (Geer et al., 2004), were used through iPortal interface for sophisticated proteomic workflows (Kunszt et al., 2015). The search parameters are: static modifications of 57.02146 Da for cysteines, variable modifications of 15.99491 Da for methionine oxidations. The parent mass tolerance was set to be 30 p.p.m and mono-isotopic fragment mass tolerance was 50 p.p.m. Fully-tryptic peptides and peptides with up to two missed cleavages were allowed. The identified peptides were processed and analyzed through Trans-Proteomic Pipeline 4.5.2 (TPP) (Keller et al., 2005) and were validated using the *PeptideProphet* score (Keller et al., 2002) . All the peptides were filtered at a false discovery rate (FDR) of 1%.

SWATH-MS measurement.

The same LC-MS/MS systems used for DDA measurements was also used for SWATH analysis (Collins et al., 2013; Gillet et al., 2012; Liu et al., 2013).

Specifically, in the present SWATH-MS mode, the SCIEX 5600 plus TripleTOF instrument was specifically tuned to optimize the quadrupole settings for the selection of 64 variable wide precursor ion selection windows. The 64-variable window schema was optimized based on a normal human cell lysate sample, covering the precursor mass range of 400–1,200 m/z. The effective isolation windows can be considered as being 399.5~408.2, 407.2~415.8, 414.8~422.7, 421.7~429.7, 428.7~437.3, 436.3~444.8, 443.8~451.7, 450.7~458.7, 457.7~466.7, 465.7~473.4, 472.4~478.3, 477.3~485.4, 484.4~491.2, 490.2~497.7, 496.7~504.3, 503.3~511.2, 510.2~518.2, 517.2~525.3, 524.3~533.3, 532.3~540.3, 539.3~546.8, 545.8~554.5, 553.5~561.8, 560.8~568.3, 567.3~575.7, 574.7~582.3, 581.3~588.8, 587.8~595.8, 594.8~601.8, 600.8~608.9, 607.9~616.9, 615.9~624.8, 623.8~632.2, 631.2~640.8, 639.8~647.9, 646.9~654.8, 653.8~661.5, 660.5~670.3, 669.3~678.8, 677.8~687.8, 686.8~696.9, 695.9~706.9, 705.9~715.9, 714.9~726.2, 725.2~737.4, 736.4~746.6, 745.6~757.5, 756.5~767.9, 766.9~779.5, 778.5~792.9, 791.9~807, 806~820, 819~834.2, 833.2~849.4, 848.4~866, 865~884.4, 883.4~899.9, 898.9~919, 918~942.1, 941.1~971.6, 970.6~1006, 1005~1053, 1052~1110.6, 1109.6~1200.5 (containing 1 m/z for the window overlap). SWATH MS2 spectra were collected from 50 to 2,000 m/z. The collision energy (CE) was optimized for each window according to the calculation for a charge 2+ ion centered upon the window with a spread of 15 eV. An accumulation time (dwell time) of 50 ms was used for all fragment-ion scans in high-sensitivity mode and for each SWATH-MS cycle a survey scan in high-resolution mode was also acquired for 250 ms, resulting in a duty cycle of ~3.45 s.

Spectral library generation and targeted data analysis.

The raw spectral libraries were generated from all valid peptide spectrum matches for the shotgun measurement of the light peptides, and then refined into the non redundant consensus libraries(Collins et al., 2013) using SpectraST (Lam et al., 2007). For each peptide, the retention time was mapped into the iRT space (Escher et al., 2012) with reference to a linear calibration constructed for each shotgun run, as previously described (Collins et al., 2013). The MS assays constructed from Top 5 most intense transitions with Q1 range from 400 to 1200 m/z excluding the precursor SWATH window were used for targeted data analysis of SWATH maps. The whole process of SWATH targeted data analysis was carried out using OpenSWATH (Rost et al., 2014). Based the spectral library generated above, OpenSWATH firstly identified the peak groups from all individual SWATH maps at a global peptide FDR=1% and then aligned them between SWATH maps (a total of 12 files including technical and biological replicates) based on the clustering behaviors of retention time in each run with a non-linear alignment algorithm (Weisser et al., 2013). Specifically, only those peptide peak groups identified in more than 75% samples (i.e., 9 files) were reported and considered for alignment with the max extension FDR of 0.05 (quality cutoff to still consider a feature for alignment) and/or the further constraint of less than 60 second RT difference in LC gradient after iRT normalization (Liu et al., 2015). The imputed data generated from the requantification option in OpenSWATH was not used.

Peptide selection and SRM measurement.

The peptide selection of SRM was directed mainly by shotgun identification results and also the prediction of MS peptide detectability using CONSeQuence software (Eyers et al., 2011) for those targeted transcripts without shotgun identification. Isotopically-labeled heavy forms (containing

either a C-terminal [$^{13}C6^{15}N4$] Arg or [$^{13}C6^{15}N2$] Lys residue) of selected peptides were synthesized by JPT Peptide Technologies. After synthesis, all peptides were resuspended in 20 % acetonitrile, 1 % formic acid and sonicated for 15 minutes. These heavy isotope-labeled peptides were then diluted into 2 % acetonitrile containing 0.1 % formic acid during the preparation of injections. Peptide samples were analyzed on a hybrid triple quadrupole/ion trap mass spectrometer (5500QTRAP, AB Sciex) equipped with a nanoelectrospray ion source. Chromatographic separation of peptides was performed by a nanoLC ultra 1Dplus system (Eksigent) coupled to a 15 cm fused silica emitter. Peptides were separated in a 35 minutes gradient of 5 – 35% acetonitrile in 0.1 % formic acid (v/v) at a flow rate of 300 nL/min (Huttenhain et al., 2012; Liu et al., 2013). Both Q1 and Q3 operated at unit resolution and a cycle time of 3s at scheduled mode (8 min window). To keep enough dwell time, the whole method was split into around 410 transitions per run. CEs were calculated according to previous studies (Lange et al., 2008; Liu et al., 2013). SRM data was manually inspected and analyzed using Skyline (MacLean et al., 2010) and normalized based on the heavy peptide standards. Finally 187 peptides were confidently quantified by SRM with reliable light/heavy pairs, of which 51 peptides mapped to 17 differentially used transcripts.

Assignment of peptides to transcripts

An initial set of 16,779 peptides was detected across biological replicates for each condition (control siRNA and PRPF8-depleted samples) using SWATH mass spectrometry and mapped against all the protein coding transcripts annotated in Ensembl v66, including those with a nonsense-mediated decay biotype. Removal of peptides that mapped to more than one gene led to a set of 14,695 peptides (corresponding to 2,805 genes), which was used for

downstream analysis. Peptides were assigned to specific transcripts as outlined in Figure 1. Peptides that map uniquely to each transcript represented a minority of events (2974 peptides mapping to 859 genes). Peptides that map ubiquitously to several transcripts of the same gene were assigned based on knowledge from the RNA-sequencing experiments using the following criteria. Two alternative peptide assignment strategies were considered. One strategy incorporated information on transcript isoform abundance for each gene into our analysis, whereby only peptides that map to major transcripts were considered. Major transcripts are the dominant expressed isoform for each gene and those identified as major in either control siRNA-treated or PRPF8 depleted samples were used specifically for peptide assignment. Additionally, we considered an alternative assignment strategy where information about transcript expression levels was not considered. Specifically, if a peptide maps to multiple transcripts in the same gene, but the expression of only one of these transcripts was changed after PRPF8 depletion, then this peptide was assigned to that particular transcript regardless of its expression level. In contrast, peptides that map simultaneously to multiple differentially used transcripts were considered ambiguous and were not used for further analysis.

Integration of transcriptomic and proteomic data

To integrate transcriptomic and proteomic data, fold-changes in transcript and peptide expression after PRPF8 depletion were obtained from RNA-sequencing and SWATH or SRM mass spectrometry experiments, respectively. RNA-sequencing fold-changes were calculated from the transcript-level expression estimates obtained from MMSEQ as described above. For each transcript, the fold-change represents the median transcript

expression in PRPF8 depleted vs. control siRNA treated samples.

Raw peptide intensities were first quantile-normalised in order to enable comparison across samples. For each peptide, the observed intensities across the biological replicates in each condition were summarised by using the median, and a fold-change was obtained by dividing the value obtained for PRPF8 depleted and control siRNA-treated samples. Peptide fold-changes for each transcript were calculated by first adding up the intensities of all the peptides that mapped to that transcript in each given biological replicate, and then dividing the median value of the summed peptide signals for PRPF8 depletion vs. controls (hence resulting in one fold-change per transcript). The same analysis was used for both SWATH and SRM datasets. Use of an alternative strategy to determine peptide fold-changes for each transcript, whereby the fold change for PRPF8 depletion vs. controls was determined individually for each peptide to obtain the median fold-change of all peptides that mapped to that transcript, yielded similar results (see Table 3). The fold-changes derived from these two technologies were integrated as described in Figure 1. Spearman correlation was used to evaluate the relationship between transcript and peptide fold-changes, as previously suggested (Maier et al., 2009). We also used Pearson correlation as a comparison.

For the retained intron analysis from Figure 6A, a list of genes previously identified to undergo intron retention events following PRPF8 depletion was used (n=2,086) (Wickramasinghe et al., 2015). Peptides were mapped to specific genes following the approach depicted in Figure 1, except a gene-centric approach was used, in contrast to the transcript-centric approach used for DTU analysis. Peptide fold-changes for each gene were then calculated by first adding up the intensities of all the peptides that mapped to that gene in each given replicate (using all available peptide data), and then dividing the median value of the summed peptide signals for PRPF8 depletion vs. controls

(hence resulting in one fold-change per gene/protein). Significance was evaluated using a t-test (adjusted p-value < 0.1). Peptides with significant fold changes in expression were used for analysis, resulting in a data set with 743 genes (out of 2805) for SWATH, of which 270 displayed retained introns, and 473 genes that do not display intron retention.

For differential gene expression analysis in Figure 6B, differentially expressed genes were obtained with MMDIFF, using a significance threshold of 0.85 for the posterior probability. Gene expression fold-changes were then calculated from MMSEQ output using the same strategy as that used for transcripts. Protein fold-changes were calculated as above from SWATH experiments and fold-change significance was assessed with a t-test, and a p-value of 0.1 was used as the significance threshold. Spearman correlation was also used to evaluate the relationship between gene and protein fold-changes.

For Figure S1, gene ontology analysis was performed using DAVID (Huang da et al., 2009). Proteins with altered expression levels were designated as such if at least one peptide per protein displayed a fold-change of greater than 1.25 fold or less than 0.75 fold after PRPF8 depletion. In the case of protein analysis, the set of proteins detected by SWATH-MS was used as a background (n = 2805).

Data availability

All the raw data of mass spectrometry measurements (SWATH-MS and shotgun), together with the input spectral library and OpenSWATH results can be freely downloaded from ProteomeXchange Consortium (http://proteomecentral.proteomexchange.org) via identifier PXD003278. The

RNA-sequencing data can be accessed from the ArrayExpress database with the accession number E-MTAB-3021.

Supplemental References

Collins, B.C., Gillet, L.C., Rosenberger, G., Rost, H.L., Vichalkovski, A., Gstaiger, M., and Aebersold, R. (2013). Quantifying protein interaction dynamics by SWATH mass spectrometry: application to the 14-3-3 system. Nature methods.

Elias, J.E., and Gygi, S.P. (2007). Target-decoy search strategy for increased confidence in large-scale protein identifications by mass spectrometry. Nature methods *4*, 207-214.

Escher, C., Reiter, L., MacLean, B., Ossola, R., Herzog, F., Chilton, J., MacCoss, M.J., and Rinner, O. (2012). Using iRT, a normalized retention time for more targeted measurement of peptides. Proteomics *12*, 1111-1121.

Eyers, C.E., Lawless, C., Wedge, D.C., Lau, K.W., Gaskell, S.J., and Hubbard, S.J. (2011). CONSeQuence: prediction of reference peptides for absolute quantitative proteomics using consensus machine learning approaches. Molecular & cellular proteomics : MCP *10*, M110 003384.

Falkner, J., and Andrews, P. (2005). Fast tandem mass spectra-based protein identification regardless of the number of spectra or potential modifications examined. Bioinformatics *21*, 2177-2184.

Flicek, P., Amode, M.R., Barrell, D., Beal, K., Brent, S., Carvalho-Silva, D., Clapham, P., Coates, G., Fairley, S., Fitzgerald, S.*, et al.* (2012). Ensembl 2012. Nucleic Acids Res *40*, D84-90.

Geer, L.Y., Markey, S.P., Kowalak, J.A., Wagner, L., Xu, M., Maynard, D.M., Yang, X., Shi, W., and Bryant, S.H. (2004). Open mass spectrometry search algorithm. Journal of proteome research *3*, 958-964.

Gillet, L.C., Navarro, P., Tate, S., Rost, H., Selevsek, N., Reiter, L., Bonner, R., and Aebersold, R. (2012). Targeted data extraction of the MS/MS spectra generated by data-independent acquisition: a new concept for consistent and accurate proteome analysis. Molecular & cellular proteomics : MCP *11*, O111 016717.

Gonzalez-Porta, M., and Brazma, A. (2015). Identification, annotation and visualisation of extreme changes in splicing from RNA-seq experiments with SwitchSeq. http://biorxiv.org/content/biorxiv/early/2014/2006/2006/005967.full.pdf.

Huang da, W., Sherman, B.T., and Lempicki, R.A. (2009). Systematic and integrative analysis of large gene lists using DAVID bioinformatics resources. Nat Protoc *4*, 44-57.

Huttenhain, R., Soste, M., Selevsek, N., Rost, H., Sethi, A., Carapito, C., Farrah, T., Deutsch, E.W., Kusebauch, U., Moritz, R.L.*, et al.* (2012). Reproducible quantification of cancer-associated proteins in body fluids using targeted proteomics. Science translational medicine *4*, 142ra194.

Keller, A., Eng, J., Zhang, N., Li, X.J., and Aebersold, R. (2005). A uniform proteomics MS/MS analysis platform utilizing open XML file formats. Mol Syst Biol *1*, 2005 0017.

Keller, A., Nesvizhskii, A.I., Kolker, E., and Aebersold, R. (2002). Empirical statistical model to estimate the accuracy of peptide identifications made by MS/MS and database search. Anal Chem *74*, 5383-5392.
Kim, S.C., Chen, Y., Mirza, S., Xu, Y., Lee, J., Liu, P., and Zhao, Y. (2006). A clean, more efficient method for in-solution digestion of protein mixtures without detergent or urea. Journal of proteome research *5*, 3446-3452.
Kunszt, P., Blum, L., Hullár, B., Schmid, E., Srebniak, A., Wolski, W., Rinn, B., Elmer, F., Ramakrishnan, C., Quandt, A.*, et al.* (2015). iPortal: the swiss grid proteomics portal: Requirements and new features based on experience and usability considerations. Concurrency and computation : practice & experience *27*, 433-445.
Lam, H., Deutsch, E.W., Eddes, J.S., Eng, J.K., King, N., Stein, S.E., and Aebersold, R. (2007). Development and validation of a spectral library searching method for peptide identification from MS/MS. Proteomics *7*, 655-667.
Lange, V., Picotti, P., Domon, B., and Aebersold, R. (2008). Selected reaction monitoring for quantitative proteomics: a tutorial. Molecular systems biology *4*, 222.
Langmead, B., Trapnell, C., Pop, M., and Salzberg, S.L. (2009). Ultrafast and memory-efficient alignment of short DNA sequences to the human genome. Genome biology *10*, R25.
Liu, Y., Buil, A., Collins, B.C., Gillet, L.C., Blum, L.C., Cheng, L.Y., Vitek, O., Mouritsen, J., Lachance, G., Spector, T.D.*, et al.* (2015). Quantitative variability of 342 plasma proteins in a human twin population. Mol Syst Biol *11*, 786.
Liu, Y., Huttenhain, R., Surinova, S., Gillet, L.C., Mouritsen, J., Brunner, R., Navarro, P., and Aebersold, R. (2013). Quantitative measurements of N-linked glycoproteins in human plasma by SWATH-MS. Proteomics *13*, 1247-1256.
MacLean, B., Tomazela, D.M., Shulman, N., Chambers, M., Finney, G.L., Frewen, B., Kern, R., Tabb, D.L., Liebler, D.C., and MacCoss, M.J. (2010). Skyline: an open source document editor for creating and analyzing targeted proteomics experiments. Bioinformatics *26*, 966-968.
Maier, T., Guell, M., and Serrano, L. (2009). Correlation of mRNA and protein in complex biological samples. FEBS Lett *583*, 3966-3973.
Rosenberger, G., Koh, C.C., Guo, T., Rost, H.L., Kouvonen, P., Collins, B.C., Heusel, M., Liu, Y., Caron, E., Vichalkovski, A.*, et al.* (2014). A repository of assays to quantify 10,000 human proteins by SWATH-MS. Scientific data *1*, 140031.
Rost, H.L., Rosenberger, G., Navarro, P., Gillet, L., Miladinovic, S.M., Schubert, O.T., Wolski, W., Collins, B.C., Malmstrom, J., Malmstrom, L.*, et al.* (2014). OpenSWATH enables automated, targeted analysis of data-independent acquisition MS data. Nature biotechnology *32*, 219-223.
Turro, E., Astle, W.J., and Tavare, S. (2014). Flexible analysis of RNA-seq data using mixed effects models. Bioinformatics *30*, 180-188.
Turro, E., Su, S.Y., Goncalves, A., Coin, L.J., Richardson, S., and Lewin, A. (2011). Haplotype and isoform specific expression estimation using multi-mapping RNA-seq reads. Genome Biol *12*, R13.
Weisser, H., Nahnsen, S., Grossmann, J., Nilse, L., Quandt, A., Brauer, H., Sturm, M., Kenar, E., Kohlbacher, O., Aebersold, R.*, et al.* (2013). An

Automated Pipeline for High-Throughput Label-Free Quantitative Proteomics. Journal of proteome research.
Wickramasinghe, V.O., Gonzalez-Porta, M., Perera, D., Bartolozzi, A.R., Sibley, C.R., Hallegger, M., Ule, J., Marioni, J.C., and Venkitaraman, A.R. (2015). Regulation of constitutive and alternative mRNA splicing across the human transcriptome by PRPF8 is determined by 5' splice site strength. Genome Biol *16*, 201.
Wickramasinghe, V.O., McMurtrie, P.I., Mills, A.D., Takei, Y., Penrhyn-Lowe, S., Amagase, Y., Main, S., Marr, J., Stewart, M., and Laskey, R.A. (2010). mRNA export from mammalian cell nuclei is dependent on GANP. Curr Biol *20*, 25-31.
